# Design of Antibacterial Agents: Alkyl Dihydroxybenzoates against *Xanthomonas* *citri* subsp. *citri*

**DOI:** 10.3390/ijms19103050

**Published:** 2018-10-06

**Authors:** Ana Carolina Nazaré, Carlos Roberto Polaquini, Lúcia Bonci Cavalca, Daiane Bertholin Anselmo, Marilia de Freitas Calmon Saiki, Diego Alves Monteiro, Aleksandra Zielinska, Paula Rahal, Eleni Gomes, Dirk-Jan Scheffers, Henrique Ferreira, Luis Octavio Regasini

**Affiliations:** 1 Department of Chemistry and Environmental Sciences, Institute of Biosciences, Humanities and Exact Sciences, São Paulo State University, São José do Rio Preto, SP 15054-000, Brazil; carlos_polaquini@hotmail.com (C.R.P.); daiane_bertholin@hotmail.com (D.B.A.); 2 Department of Biochemistry and Microbiology, Biosciences Institute, São Paulo State University, Rio Claro, SP 13506-900, Brazil; l.bonci@hotmail.com (L.B.C.); henrique.ferreira@unesp.br (H.F.); 3 Department of Molecular Microbiology, Groningen Biomolecular Sciences and Biotechnology Institute, University of Groningen, 9747 AG Groningen, The Netherlands; a.u.zielinska@rug.nl (A.Z.); d.j.scheffers@rug.nl (D.-J.S.); 4 Department of Biology, Institute of Biosciences, Humanities and Exact Sciences, São Paulo State University, IBILCE, São José do Rio Preto, SP 15054-000, Brazil; macal131@gmail.com (M.d.F.C.S.); diego8monteiro@hotmail.com (D.A.M.); prahal@ibilce.unesp.br (P.R.); eleni@ibilce.unesp.br (E.G.)

**Keywords:** citrus canker, antimicrobial, plant disease, membrane disruption, phenolic acids

## Abstract

*Xanthomonas citri* subsp. *citri* (Xcc) causes citrus canker, affecting sweet orange-producing areas around the world. The current chemical treatment available for this disease is based on cupric compounds. For this reason, the objective of this study was to design antibacterial agents. In order to do this, we analyzed the anti-Xcc activity of 36 alkyl dihydroxybenzoates and we found 14 active compounds. Among them, three esters with the lowest minimum inhibitory concentration values were selected; compounds **4** (52 μM), **16** (80 μM) and **28** (88 μM). Our study demonstrated that alkyl dihydroxybenzoates cause a delay in the exponential phase. The permeability capacity of alkyl dihydroxybenzoates in a quarter of MIC was compared to nisin (positive control). Compound **28** was the most effective (93.8), compared to compound **16** (41.3) and compound **4** (13.9) by percentage values. Finally, all three compounds showed inhibition of FtsZ GTPase activity, and promoted changes in protofilaments, leading to depolymerization, which prevents bacterial cell division. In conclusion, heptyl dihydroxybenzoates (compounds **4**, **16** and **28**) are promising anti-Xcc agents which may serve as an alternative for the control of citrus canker.

## 1. Introduction

Citrus canker is caused by *Xanthomonas citri* subsp. *citri* (Xcc), which is responsible for a severe form of the disease (Asian citrus canker) [1,2,3]. Typical symptoms of this plant disease are brownish eruptive lesions on their aerial parts, which may be surrounded by chlorotic halos. Bacteria infect leaves, stems, and fruit, which leads to defoliation, twig dieback and early fruit drop. As a consequence, the disease brings significant economic losses to the citrus sector [3,4,5,6].

These phytopathogens enter the plant through stomata and injuries caused during crop management or by the citrus leaf miner (*Phyllocnistis citrella*). They colonize the intercellular space in the apoplast and break down the leaf epidermis as a result of cell hyperplasia [6]. Rainfall, wind and movement of infected seedlings moving are the causes for this bacterial dissemination [7]. Under current legislation, the state of São Paulo in Brazil was has been declared a Risk Mitigation System (RMS) area [8]. This is a control that aims to reduce citrus canker inoculum potential.

The chemical control of citrus canker is carried out with copper-based antibacterials [9,10,11]. Copper applications reduce the intensity of the disease and the number of inoculations into the plant, because bacterial survival is reduced [10,11,12,13,14,15,16]. Although copper sprays reduce crop loss and help to control citrus canker, they may also negatively affect the environment [17]. Copper may accumulate in the soil, and can cause adverse effects on root growth and on nutrient uptake by citrus trees [18,19,20,21,22]. In addition, the emergence of copper-resistant bacterial strains is a reality [23,24].

Phenolic compounds have been evaluated as an alternative to current citrus canker treatment. In our previous studies, potent antibacterial activities of a homologous series of alkyl gallates (3,4,5-trihydroxybenzoates) [25] were described. The antibacterial mechanism of these compounds involves action on the membrane and they promote cell death by a combination of mechanisms: FtsZ inhibition, membrane permeability, and possibly another activity [26]. In order to improve the anti-Xcc activity of alkyl gallates, four monoacetylated gallates were synthesized and tested against Xcc [27]. As expected, the insertion of the acetyl group on the alkyl gallates conferred an increase of lipophilicity on these compounds, and made them more potent than the pattern phenolic compounds [25,27]. Altogether, these data suggest that modifications in hydroxyl groups may result in increased anti-Xcc potency.

Therefore, our research group has investigated dihydroxybenzoic acids and their alkyl esters as antimicrobials against Gram-positive and Gram-negative bacteria and yeasts [28,29,30]. Besides their antimicrobial activity, we selected these compounds in order to investigate the relevance of the number and position of the hydroxyls to anti-Xcc activity. According to Nihei, Nihei and Kubo, in order to elicit activity as the hydrophilic ‘head’ portion, two hydroxyl groups are essential [29].

In our continuing search for anti-Xcc agents from phenolic natural products-based compounds, we synthesized three series of alkyl esters from β-resorcilic, gentisic and protocatechuic acids. The anti-Xcc activity of these esters was evaluated against X. *citri* subsp. *citri* strain 306 (IBSBF 1594) to derive preliminary structure-activity relationships. The three most active compounds (**4**, **16** and **28**) were investigated regarding their toxicity against eukaryotic cell lines and their effects on the growth of Xcc. In addition, their ability to act on membrane and cell division by inhibition of the GTPase activity of FtsZ were carried out.

## 2. Results

### 2.1. Synthesis, Purification and Identification of Alkyl Dihydroxybenzoates

The new antibacterial agents were synthesized in three different series with 12 alkyl dihydroxybenzoates each: series I (**1**–**12**), series II (**13**–**24**) and series III (**25**–**36**). Each series had carboxylic acids as precursors; they were the 2,4 dihydroxybenzoic (or β-resorcilic) acid (I), the 2,5 dihydroxybenzoic (or gentisic) acid (II) and the 3,4 dihydroxybenzoic (or protocatechuic) acid (III). Different purification methods were used in order to obtain the pure compounds; low temperature hexane washes, hexane and acetone crystallization techniques, and normal phase column chromatography. The purity degree analysis was carried out with high-performance liquid chromatography (HPLC), and was measured by integrating the peaks of the chromatograms. The values varied from 76.5% to 99.9% (Table 1), thus validating our methodologies for purifying and obtaining the compounds. The alkyl dihydroxybenzoates structures were determined by ^1^H and ^13^C NMR analyses. Spectra and chromatograms are presented as supplementary material.

### 2.2. Partition Coefficient Study

The partition coefficients were measured by HPLC method according to OECD 117 – Guidelines for the Testing of Chemicals [31] to all compounds. The log *P*_o/w_ values of the alkyl dihydroxybenzoates and carboxyl acids were ranged from 0.8 to 7.0 (Table 1).

### 2.3. Determination of MIC and MBC against Xanthomonas citri subsp. citri Assay

The antibacterial potential of carboxylic acids and their esters were compared to copper oxychloride (a commercial active often used to combat citrus canker). Minimal inhibitory concentration (MIC) values were determined by REMA assay, which measures cellular respiratory activity.

We assayed the carboxylic acids (2,4 dihydroxybenzoic acid, 2,5 dihydroxybenzoic acid and 3,4 dihydroxybenzoic acid) which demonstrated they were inactive since they did not inhibit Xcc growth (MIC > 100 μg mL^−1^). However, 14 esters showed potential anti-Xcc activity, with MIC values lower than 100 μg mL^−1^ (**2**–**4**, **11**, **15**, **16**, **22**–**24**, **27**–**30** and **36**).

The minimal bactericidal concentration (MBC) was determined by observing the bacterial growth in Petri dishes transferred from the REMA plates. We observed bactericidal action for compounds **11**, **22**–**24**, **31** and **36** at 100 μg mL^−1^, compounds **2**, **3**, **15**, **27**–**30** at 50 μg mL^−1^ and compounds **4** and **16** at 25 μg mL^−1^.

### 2.4. Cytotoxicity Assay

The cytotoxicity of the three most potent compounds (**4**, **16** and **28**) was evaluated using the MTT test. This test is based on the reduction of 3-(4,5-dimethyl-2-thiazolyl)-2,5-diphenyl-2-tetrazole bromide by viable cells, with formation of violet insoluble formazan crystals. Five cell lines were used in order to evaluate the cytotoxic effect of the compounds as a whole.

Thus, it can be seen in Table 2 that compounds **4**, **16** and **28** had low cytotoxic potential because they showed IC_50_ values above 100 μM for MRC-5 (human lung fibroblast cell line), VERO (monkey kidney epithelial cell line) and ACHN (human renal adenocarcinoma cell line). In contrast, HaCaT (human keratinocyte cell line) and Huh-7.5 (human hepatoma cell line) were more sensitive to selected compounds, with IC_50_ values in the range of 70 μM, with the exception of compound **16** (20.60 μM) for Huh-7.5. The kanamycin (control) and copper oxychloride showed IC_50_ values above 200 μM and 200 μg mL^−1^, respectively.

### 2.5. Growth Curve

**Figure 1:**
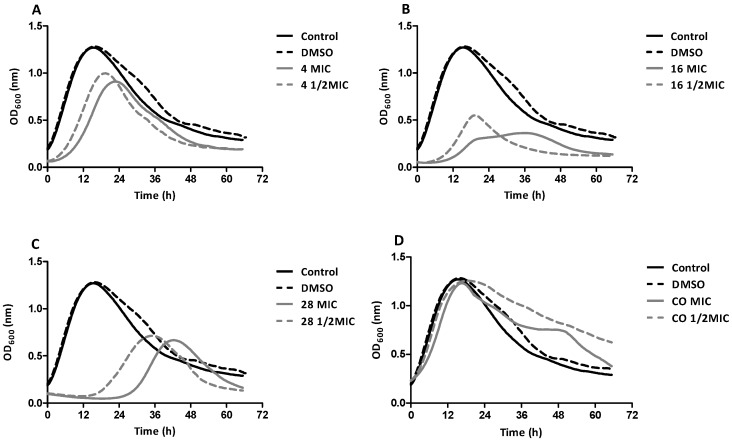
Growth curve profiles of *Xanthomonas citri* subsp. *citri* with treatments in MIC and ½ MIC values of alkyl dihydroxybenzoates for 72 h. **A** Compound **4**, **B** compound **16**, **C** compound **28**, and **D** copper oxychloride (CO) in comparison to control (untreated) and DMSO (vehicle control).

According to the growth curves of the positive control (PC) and vehicle control (DMSO), it was observed that Xcc reaches the maximum growth phase in a period of 15 h. The growth rates of Xcc under the effects of dihydroxybenzoic acids (DA) **4**, **16** and **28** were measured at concentrations equivalent to MIC and ½ MIC. The early growth inhibition (lag phase), which happens in the first hours of contact (period of the first cellular divisions) can be a dynamic process. The lag phase delay was observed in the three compounds tested, and it showed that the higher the concentration, the higher the death rate. This can be explained because the compounds have bacteriostatic action; that is, as the time passes, the cells become able to recover and restart doubling. Another important finding is that the lag phase consists in the cell adaptation to the new medium conditions and the bacterial population is smaller in relation to the exponential phase facilitates the action of the compounds in the first hours (Figure 1) [32]. Observing the exposure of Xcc to compounds **4**, **16** and **28** it can be seen that there was a delay in the exponential phase, prolonging the time taken for the bacteria to reach the stationary phase.

When we evaluated the concentration-response behavior of the compounds, we obtained a decrease in the curves with the concentration of the ½ MIC. Compared to the PC and DMSO, it was more evident in the MIC curve, which showed that for all three compounds, the higher the concentration, the greater the influence on Xcc.

### 2.6. Cellular Membrane Permeability of Xcc Using the Live/Dead Kit

**Figure 2:**
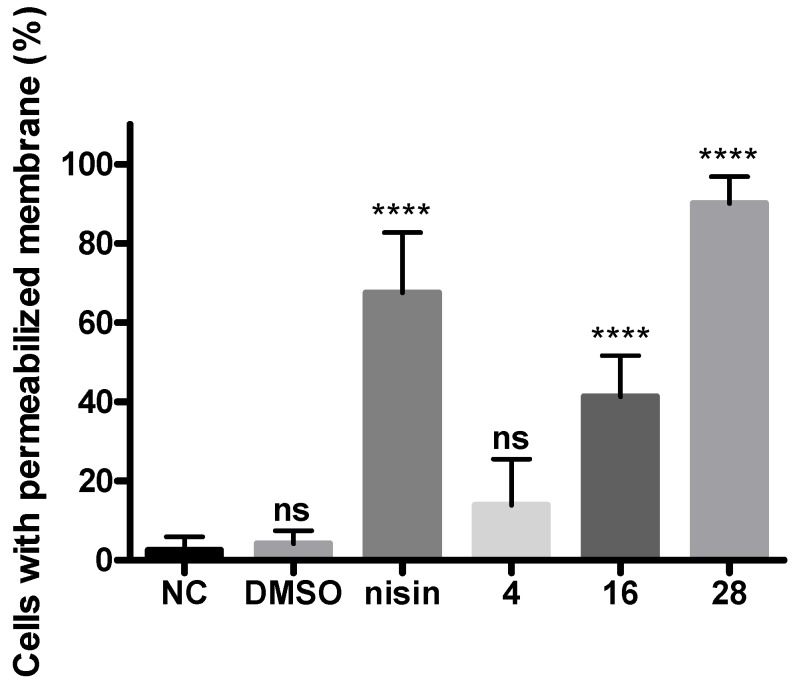
Cellular permeability evaluation in the presence of alkyl dihydroxybenzoates (**** *p* < 0.0001, ANOVA with Dunnett’s post-test), ns = not significant.

Membrane integrity was measured by using the commercial assay Live/Dead BacLight^®^ bacterial viability kit (Invitrogen, Carlsbad, CA, USA). We observed cells with intact membrane in fluorescent blue (SYTO 9) and cells with disturbance or affected membrane integrity in red or pink (propidium iodide). The data analyses were plotted as the percentage of cells permeated by propidium iodide (Figure 2).

The nisin was used as a positive control for causing pores in the membrane [26]. When we observed the permeability capacity of alkyl dihydroxybenzoates in ¼ MIC and compared these to nisin (79.47%), we noticed that compound **28** was the most effective, because its permeation ability reached 93.8%, while for compound **16** it was 41.3%, and for compound **4**, 13.9%.

**Figure 3:**
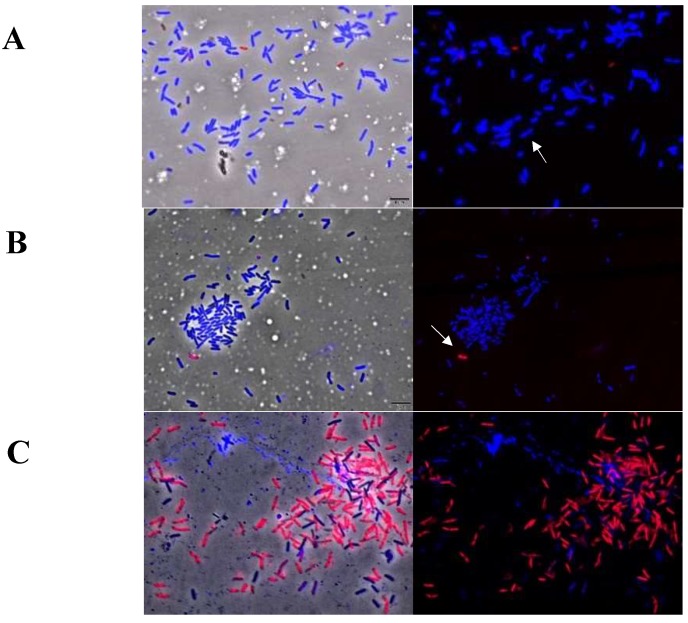

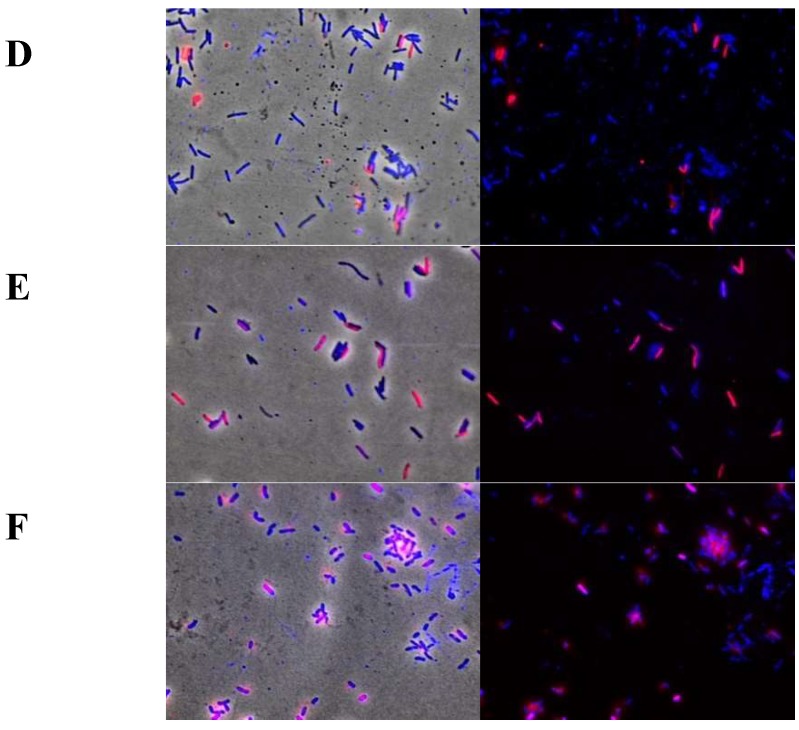
Optical microscopy of fluorescence images at ¼ MIC of alkyl dihydroxybenzoates for 15 min. (**A**) Negative control (untreated), (**B**) vehicle control (DMSO), (**C**) positive control (nisin), (**D**) compound **4**, (**E**) compound **16,** and (**F**) compound **28**.

Permeation percentages were obtained by fluorescence optical microscopy (Figure 3). Compound **28** showed high permeability, evidencing the entry of propidium iodide (PI) into cells. This behavior is similar to that of the positive control (nisin). For this reason, the target of compound **28** is membrane- related.

### 2.7. Inhibition Assays of GTPase Activity

The cell division process is mediated by the formation of the Z ring, coordinated by GTP hydrolysis activity of FtsZ [33,34,35]. Because of this, we evaluated the inhibition of GTPase activity of purified FtsZ from Xcc, incubated with compounds **4**, **16** and **28** using the continuous substrate regeneration assay [36].

When evaluating the behavior of compounds **4**, **16** and **28**, we could see decay in the GTPase activity of FtsZ. These data suggest that all three compounds promoted changes in protofilaments, leading to depolymerization, which reduces bacterial cell division.

## 3. Discussion

*Xanthomonas* is one of the most important genera among the phytopathogens. It causes diseases in economically important cultivars such as rice, beans, tomatoes, oranges, peppers, passion fruit, grapes and others [5].

In this study, we have focused our work on Xcc, which is the main cause of citrus canker. The chemical control of citrus canker is mainly through application of cupric compounds, but the emergence of copper-resistant strains has encouraged research into new compounds [37,38,39]. Recently, other effective compounds against Xcc have been reported, such as bismerthiazol [40] and 3-indolylacetonitrile [41], which are promising agents against citrus canker.

The 2,4 DHB, 2,5 DHB and 3,4 DHB acids were inactive (MIC > 100 μg mL^−1^). Then, we evaluated three ester homologous series containing ethyl, butyl, hexyl, octyl, decyl and dodecyl dihydroxybenzoates. In general, hexyl and octyl dihydroxybenzoates displayed potent activity against Xcc. Thus, we decided to evaluate heptyl and nonyl esters. Heptyl dihydroxybenzoates **4**, **16** and **28** exhibited the lowest MIC values. Subsequently the branched, cyclic and aromatic esters were evaluated to analyze the hypothetical effects of stereoelectronic parameters on the anti-Xcc effect. However, clear conclusions about the relationship between structure and activity were not established. Copper oxychloride showed anti-Xcc activity, with the MIC value of 43.1 μg mL^−1^.

In our previous study, we described the anti-Xcc activity of alkyl gallates (alkyl 3,4,5-trihydroxybenzoates). In order to investigate the relevance of number and position of hydroxyl groups on anti-Xcc activity, alkyl dihydroxybenzoates were evaluated. Heptyl dihydroxybenzoates (**4**—52 μM, **16**—80 μM and **28**—88 μM) were more active than heptyl gallate (MIC = 116 μM) [25]. This set of data indicated that reduction in the number of hydroxyls increased anti-Xcc activity. Comparisons among MIC values of compounds **4**, **16** and **28** suggested the hydroxyls at positions 2 and 4 (compound **4**) increased anti-Xcc activity when compared to positions 2 and 5 (compound **16**), as well as 3 and 4 (compound **28**).

This was corroborated using other data regarding the dihydroxybenzoic acids (DA) and their esters that had antimicrobial activity described, against Gram-positive and Gram-negative bacteria [42,43,44,45,46,47,48] and yeasts [29]. Some DAs, such as 2,3; 2,4; 2,5; 2,6 and 3,4 show activity against *Campylobacter jejuni*, *Escherichia coli*, *Listeria monocytogenes* and *Salmonella enterica* [30].

Merkl and collaborators studied the antimicrobial activity of the phenolic acids and their alkyl esters, against *E.* *coli*, *Bacillus cereus*, *L.* *monocytogenes*, *Saccharomyces cerevisiae* and *Fusarium culmorum* and reported significant differences between values of MIC of acids and their esters. In general, butyl esters were twice as potent as their acids. In addition to this, the authors associated the increase of antibacterial activity with the size of the side alkyl chain [28].

With the increase of the alkyl chain, a considerable decrease in solubility occurs in aqueous medium (culture medium). This favors the formation of micelles, which are not able to cross bacterial cells. The amphiphilic character of the alkyl dihydroxybenzoates allows the micellar formation due to the hydrophilicity of the hydroxyl groups on the phenolic ring, and the long carbonic chain is highly hydrophobic [49].

The compound with the highest lipophilicity was the dodecyl 2,4 dihydroxybenzoate (**8**), and the compound with the lowest lipophilicity was the 3,4 dihydroxybenzoic acid (**3,4-DHB acid**). As the side chain increased, the lipophilicity of these compounds increased; that is, the interaction of the compounds with the C18 column was more intense, culminating in longer retention times, being slightly different for the branched and cyclic compounds. Compound **4** has two hydroxyls at the 2,4 (meta) positions, compound **16** has two hydroxyls at the 2.5 (para), and compound **28** has another two at the 3,4 (ortho) positions. The log *P*_o/w_ values of 5.1 (compound **4**), 5.0 (compound **16**) and 4.3 (compound **28**) obtained through the partition coefficient are relatively close, with compound **4** being more lipophilic.

Branched compounds **9** (2.6), **21** (2.2) and **33** (1.4) presented smaller than their isomers **2** (3.9), **14** (3.4) and **26** (2.6). This is due to the fact that branching reduces the size of the molecule, decreasing the surface area and making it difficult to solvate in the non-polar phase [50], similar to cyclic compounds.

In this work, we demonstrated that heptyl dihydroxybenzoates had no cytotoxicity. Our results corroborate those of Medina-Alarcón and colleagues (2017), who reported that protocatechuates did not show cytotoxicity in MRC-5 and HepG2 cell lines [51].

When we observed the growth curves treated with the compounds, we noticed a delay in the exponential phase, which prolonged the time taken for the bacterium to reach the stationary phase. This delay is more evident when increasing the concentration of the compounds; the greater the concentration, the greater the influence on Xcc. The ester interactions with Xcc showed different curves, which is an interesting fact, since the esters have the same side carbonic chain size. Therefore, this made us consider the influence of hydroxyl positions on kinetics growth. Concerning the growth curve, it was observed that, compared to the PC and the DMSO, Xcc bacterial growth suffered a delay of approximately 18 h to reach the stationary phase. However, when comparing the compounds, **16** demonstrated a greater inhibition of bacterial growth, decreasing the height of the curve significantly; that is, with a lower optical density. Therefore, it can be inferred that its action on Xcc is greater than the action of compound **4**. The profile of compound **28** showed increased bacterial growth delay of 20 to 30 h, and even beyond this time it continued to inhibit the growth of Xcc more effectively than compound **4**, but with less killing capacity than compound **16** (Figure 1).

When we evaluated copper oxychloride, we found an MIC value of 43.1 μg mL^−1^ to potential anti-Xcc activity on cell lines tested that did not demonstrate cytotoxicity. On the growth curves treated with the copper oxychloride, we noticed that the curve pattern did not change and the copper oxychloride was inactive under the conditions tested. Behlau and colleagues (2017), described copper oxychloride as an insoluble copper formulation (ICuF) (syn. fixed copper), and being the most studied and commonly used form of copper for the control of citrus canker [12,17], this could explain the low activity of copper oxychloride in our trials.

The minimal bactericidal concentration was determined by observing the bacterial growth in Petri dishes transferred from the REMA plates. We obtained bactericidal action in compounds **11**, **22**–**24**, **31** and **36** at the concentration of 100 μg mL^−1^, in compounds **2**, **3**, **15**, **27**–**30** at the concentration of 50 μg mL^−1^ and in compounds **4** and **16** at the concentration of 25 μg mL^−1^.

Finally, we investigated the alkyl dihydroxybenzoates for interference in the modulation of FtsZ cell-mediated Xcc cell division [52]. FtsZ is strongly related to α/β-tubulin in three-dimensional structure [53], the possession of GTPase activity [35,54] and the ability to polymerize in a nucleotide-dependent manner in vitro [34,55]. The continuous substrate regeneration assay was performed to characterize GTPase activity similar to bacterial self-assembling tubulin, FtsZ [56]. The compounds **4**, **16** and **28** showed decay in the GTPase activity of FtsZ. These data suggest that all three compounds promoted changes in protofilaments, leading to depolymerization, which prevents bacterial cell division.

All compounds affected membrane integrity (Figure 2), but only compound **28** showed high permeability similar to nisin. Therefore, similar to alkyl gallates [26], compounds **4**, **16** and **28** possibly promote cell death by some combination of the FtsZ inhibition and membrane permeabilization mechanisms. 

In summary, three series of alkyl dihydroxybenzoates were designed and synthesized as part of our ongoing search for anti-Xcc agents based on natural phenolic compounds. The heptyl dihydroxybenzoates (compounds **4**, **16** and **28**) displayed potent anti-Xcc activity. Furthermore, due to preliminary low toxicity against eukaryotic cells, as well as the ability to act on membrane and cell division, these compounds are promising agents against citrus canker.

## 4. Methods

### 4.1. Synthesis of Alkyl Dihydroxybenzoates

**Scheme 1:**
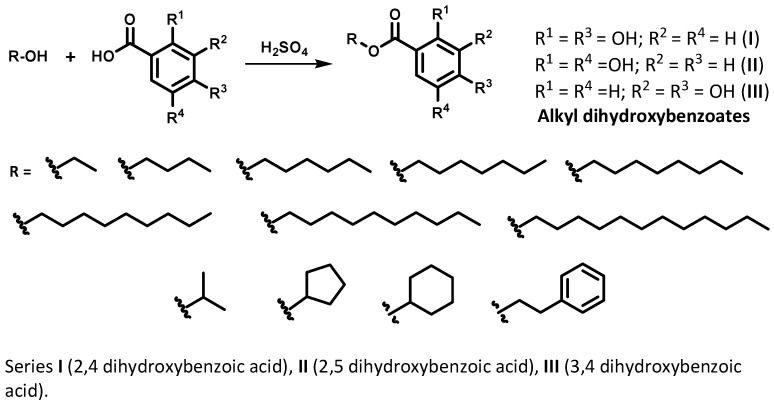
Preparation of alkyl dihydroxybenzoates by Fischer’s Esterification.

The esters of dihydroxybenzoic acids were prepared by Fischer’s Esterification (Scheme 1) methodology with modifications [25,57]. 6 mmol of carboxylic acids were dissolved in 18 mmol of alkyl alcohols (ethanol, 1-butanol, 1-hexanol, 1-heptanol, 1-octanol, 1-nonanol, 1-decanol, 1-dodecanol, 2-propanol, cyclopentanol, cyclohexanol and 2-phenylethanol) under constant magnetic stirring. After complete dissolution, catalytic amounts of sulfuric acid (1–3 drops) were added. The reactions were maintained at temperatures close to boiling point for the respective alcohols and were conducted for a period of 1–3 days. The progress of the reaction was checked by thin layer chromatography (TLC) [7:3 *n*-hexane: ethyl acetate]. After the conversion, the residue was partitioned 3 times with ethyl acetate. Later, it was washed with distilled water (3 times) and 1 mol L^−1^ sodium bicarbonate solution (3 times). The organic phase was dried using a rotary evaporator. The crude products were purified with a low temperature hexane washes, crystallized with hexane or acetone and over normal phase column chromatography (silica gel 0.06–0.20 mm, ACROS Organics, New Jersey, USA). All compounds were identified with the use of NMR spectra on a Bruker Avance III 14.1 T, Bruker Avance III 9.4 T and Bruker Fourier 7.1 T spectrometer, using deuterated chloroform (CDCl_3_) or deuterated dimethylsulfoxide (DMSO-*d_6_*) as solvents. Compounds were analyzed by HPLC for determination of their purity.

### 4.2. Partition Coefficient Determination Log P_o/w_ by HPLC

The log *P*_o/w_ were determined using the HPLC procedure according to OECD Guidelines for the Testing of Chemicals [31,58] on a system equipped with a binary pump, automatic sampler column (Agilent^®^ Technologies, Santa Clara, CA, USA, model 1220 Infinity), and photodiode array system Zorbax Eclipse Plus C-18 column (4.6 × 250 mm, 5 μm, Agilent^®^ Technologies, Santa Clara, CA, USA). The analyses were performed with the use of an isocratic flow in methanol: water (3:1) at 1.0 mL min^−1^. We injected 20.0 μL and analyses were performed at 254 nm. We used as reference compounds thiourea, phenol, acetophenone, benzoic acid, benzene, toluene, biphenyl, fluoranthene and triphenylamine in order to construct the curve log *K* × log *P*_o/w_. The lipophilicity of alkyl dihydroxybenzoates was determined from their retention times and interpolation in linearity curve log *K* × log *P*_o/w_. We performed the analyses in duplicate.

### 4.3. Determination of MIC and MBC against Xanthomonas citri subsp. citri Assay

The antibacterial activity of alkyl dihydroxybenzoates and the determination of minimum inhibition concentration (MIC) were performed by the fluorimetric method Resazurin Microtiter Assay (REMA) with some modifications [25,59]. An overnight culture of *X. citri* subsp. *citri* strain 306 (IBSBF 1594) [60] was grown in 20 mL in Nitrogen Yeast Glycerol (NYG) at 29 °C in constant agitation of 180–200 rpm. After 12 h, the Xcc inoculum was dispersed in NYG medium and distributed into a 96 well microtiter plate to a final volume of 100 μL per well (10^5^ CFU per well). Stock solutions of tested compounds at 10 mg mL^−1^ were prepared in dimethyl sulfoxide (DMSO) and dispersed in NYG medium to obtain 1% DMSO and the final compound concentration range of 0.78–100 μg mL^−1^. The negative control was constituted of 100 μL culture medium. In addition, the copper oxychloride was tested as a comparison to alkyl dihydroxybenzoates and kanamycin (20 μg mL^−1^), which was used as a control antibiotic. The plate was subsequently incubated at 29 °C for 12 h, and 15 μg mL^−1^ freshly prepared resazurin solution in water was added to each well, and the plate was incubated for 2 h. The fluorescence was determined on a Synergy H1 (BioTek, Winooski, VT, USA) microplate reader using the excitation and emission filters at wavelengths of 530 and 590 nm respectively. After the REMA assay, the minimum bactericidal concentration (CBM) assay was performed using a 96-pin stainless steel plate replicator, accurately transferring the wells from the REMA plate to Petri dishes Petri dishes were incubated in oven B.O.D. at 29 °C for 24 to 48 h [61]. The experiments were performed in triplicate.

### 4.4. Determination of Cytotoxicity

The cytotoxicity of compounds **4**, **16**, **28** and copper oxychloride was determined by MTT assay [62]. The cell lines used were HaCaT (human keratinocyte cell line—CLS 300493), Huh-7.5 (human hepatoma cell line—ATCC^®^ PTA-8561), MRC-5 (human lung fibroblast cell line—ATCC CCL-171), VERO (monkey kidney epithelial cell line—ATCC^®^ CCL81) and ACHN (human renal adenocarcinoma cell line—ATCC^®^ CRL1611). The cells were cultured in appropriate medium recommended by the American Type Culture Collection.

The assay was performed with the concentration of 10^4^ cells for both cell lines, distributed in 96-well plates and incubated at 37 °C in a 5% CO_2_ controlled atmosphere oven for 24 h. After the incubation period, the cells were treated with the compounds with a range of 1.56–200 μmol L^−1^ and incubated for 24 h. The supernatant was removed, then 100 μL of 3-(4,5-dimethyl-2-thiazolyl)-2,5-diphenyl-2*H*-tetrazolium (MTT) salt at 1 mg mL^−1^ diluted in DMEM medium (Gibco Life Technologies, Gaithersburg, MD, USA) was added and incubated for 30 min. Subsequently, the MTT was removed, then 100 μL of DMSO under agitation for 5 min at 60 rpm was added. The plates were read at 570 nm in a FLUOstar Omega/BMG LABTECH plate reader, Offenburg, BW, DE. We carried out the experiments in triplicate and three independent events for statistical analysis (GraphPad Software, San Diego, CA, USA). The MTT assay is based on the tetrazolium salt (yellow-colored) reduction to formazan (purple-colored) by metabolically active cells [63].

### 4.5. Growth Curve

An isolated colony of *Xanthomonas citri* subsp. *citri* was inoculated in NYG medium for 12 h at 29 °C. After this, the optical density (OD 600 nm) was measured and adjusted to ~0.1. Positive controls (cells and culture medium) and cells with treatments of compounds **4**, **16** and **28** were then prepared in MIC and ½ MIC values. Optical density reading was measured on a Synergy H1-BioTek plate reader every 30 min for 72 h. The growth curves were plotted with GraphPad Prism software. At the same time, the degradation assay was performed, solubilizing the compounds in DMSO, which were later incorporated in NYG medium. Later, we scanned the compounds to measure the absorbance values from 210 to 700 nm, and the maximum lambda value of each compound was selected after scanning. After that, punctual readings were carried out every 30 min for 72 h.

### 4.6. Cellular Membrane Permeability of Xcc Using the Live/Dead Kit

Phase contrast microscopy was used to observe changes in cell shape and signs of cell division alteration using an objective lens with a magnification of 100×. To evaluate the Xcc cells’ membrane integrity, the BacLight^®^ kit was used. Mixtures of SYTO 9 dyes and propidium iodide (PI). SYTO 9 (blue) can stain nucleic acids in general—with intact or damaged membranes. On the other hand, PI (red) penetrates only the bacteria with damaged membranes, causing overlap in the fluorescence of SYTO 9. Therefore, using a mixture of the dyes, the bacteria with disturbed cell membranes are stained in fluorescent red and the intact cell membranes are stained in fluorescent blue. The Xcc cells at density of 10^6^ cells mL^−1^ were submitted to 15 min treatments with compounds **4**, **16** and **28** at ¼ MIC values. Cellular immobilization was performed on slides containing 1% agarose gel in 0.85% saline buffer. The slides were prepared with 1.0 mL of the agarose solution, which was dispersed in optical microscopy slides and on each slide another slide was placed so that the gel continued like a thin film and was of homogeneous height. The slides were dried at room temperature for approximately 20 min and opened at the time of use, when 10 μL of the cell suspension was deposited in the center and covered with the cover slip. The cells were visualized using the Olympus BX-61 optical microscope equipped with OrcaFlash 2.8 monochrome camera (Hamamatsu, Shizuoka, Japan). Cell Sensation software v11 (Olympus) was used to document and analyze the images.

### 4.7. Inhibition Assays of GTPase Activity

The evaluation of the inhibition of GTPase activity was measured by the characterization of the FtsZ from Xcc [36,57]. This methodology is known as continuous assay of substrate regeneration and consists of the hydrolysis of guanosine triphosphate (GTP) in guanosine diphosphate (GDP) and inorganic phosphate. The GTP is regenerated by the enzyme pyruvate kinase which, in turn, converts the phosphoenol pyruvate (PEP) to pyruvate. The pyruvate produced by this reaction is reduced to lactate by lactate dehydrogenase, using NADH [64].

Reagent stocks were dispersed in Tris 50 buffer (1:1 tris:KCl at pH 7.9). We also prepared two mixtures: Mixture 1—magnesium chloride (MgCl_2_), pyruvate kinase/lactate dehydrogenase, phosphoenol-pyruvate, NADH and GTP, adding or not the test compounds to MIC values; and mixture 2—Xcc-FtsZ. The assay was performed in a 96-well plate, 75 μL of mixture 1 was pipetted into a well and incubated for 5 min at 30 °C; then 75 μL of mixture 2 was added and the readings started. A Biotek Synergy MX plate reader was used. The readings were performed every 5 s, at the wavelength of 340 nm at 30 °C, under constant stirring.

The total volume of 150 μL presented the following concentrations: 5 mM MgCl_2_; pyruvate kinase/lactate dehydrogenase 20 U mL^−1^; 2 mM phosphoenol-pyruvate; 1 mM NADH; 400 μM GTP; Xcc-FtsZ 5 μM.

### 4.8. Statistical Analysis

**Figure 4:**
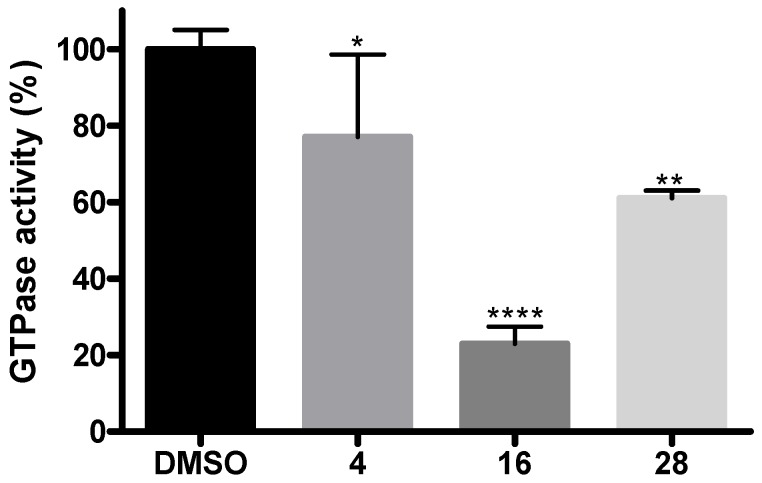
Inhibition of GTPase activity of alkyl dihydroxybenzoates in MIC value (* *p* < 0.04, ** *p* < 0.001, **** *p* < 0.0001, ANOVA with Dunnett’s post-test).

All assays were performed in triplicate. The data were analyzed using one-way analysis of variance (ANOVA) followed by Tukey’s test for Table 2, and Dunnett’s test for Figure 2 and Figure 4. The data analyses were plotted with GraphPad Prism version 5.0 (GraphPad, San Diego, CA, USA).

## Figures and Tables

**Table 1:** Antibacterial activities against *Xanthomonas citri* subsp. *citri*, purity and log *P*_o/w_ of dihydroxybenzoates **1**–**36**.

	Entry	R	Purity	log *P*_o/w_	MIC μg mL^−1^ (MIC in μM)	MBC μg mL^−1^
Series I 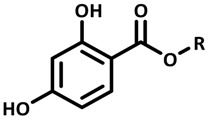	**2,4 DHB acid**	H	85.7	2.6	>100 (>648)	>100
	**1**	CH_2_CH_3_	87.6	2.8	>100 (>548)	>100
	**2**	(CH_2_)_3_CH_3_	85.6	3.9	39.8 (189)	50
	**3**	(CH_2_)_5_CH_3_	81.7	5.0	20.0 (84)	50
	**4**	(CH_2_)_6_CH_3_	83.8	5.1	13.2 (52)	25
	**5**	(CH_2_)_7_CH_3_	79.6	6.1	>100 (>375)	>100
	**6**	(CH_2_)_8_CH_3_	99.0	6.2	>100 (>356)	>100
	**7**	(CH_2_)_9_CH_3_	99.5	6.9	>100 (>340)	>100
	**8**	(CH_2_)_11_CH_3_	>99.9	7.0	>100 (>310)	>100
	**9**	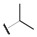	93.2	2.6	>100 (>510)	>100
	**10**	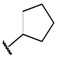	>99.9	2.2	>100 (>450)	>100
	**11**	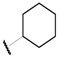	>99.9	4.0	40.5 (171)	100
Series II 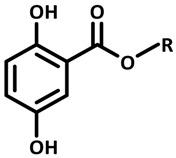	**2,5 DHB acid**	H	97.3	2.8	>100 (>648)	>100
	**13**	CH_2_CH_3_	98.3	2.4	>100 (>549)	>100
	**14**	(CH_2_)_3_CH_3_	82.0	3.4	>100 (>475)	>100
	**15**	(CH_2_)_5_CH_3_	80.1	4.5	33.5 (140)	50
	**16**	(CH_2_)_6_CH_3_	99.3	5.0	20.2 (80)	25
	**17**	(CH_2_)_7_CH_3_	79.8	5.2	>100 (>375)	>100
	**18**	(CH_2_)_8_CH_3_	>99.9	5.7	>100 (>357)	>100
	**19**	(CH_2_)_9_CH_3_	76.5	6.3	>100 (>340)	>100
	**20**	(CH_2_)_11_CH_3_	>99.9	6.8	>100 (>310)	>100
	**21**	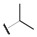	>99.9	2.2	>100 (>510)	>100
	**22**	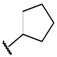	>99.9	3.0	83.5 (375)	100
	**23**	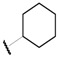	>99.9	3.5	90.4 (382)	100
	**24**	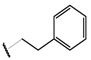	>99.9	3.1	59.7 (231)	100
Series III 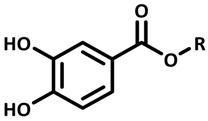	**3,4 DHB acid**	H	99.6	0.8	>100 (>648)	>100
	**25**	CH_2_CH_3_	99.1	1.7	>100 (>549)	>100
	**26**	(CH_2_)_3_CH_3_	99.1	2.6	>100 (>475)	>100
	**27**	(CH_2_)_5_CH_3_	99.1	3.7	44.4 (186)	50
	**28**	(CH_2_)_6_CH_3_	98.5	4.3	22.2 (88)	50
	**29**	(CH_2_)_7_CH_3_	98.5	4.9	31.2 (117)	50
	**30**	(CH_2_)_8_CH_3_	97.9	5.5	26.7 (95)	50
	**31**	(CH_2_)_9_CH_3_	91.3	6.0	>100 (>340)	100
	**32**	(CH_2_)_11_CH_3_	>99.9	6.9	>100 (>310)	>100
	**33**	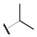	>99.9	1.4	>100 (>510)	>100
	**34**	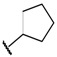	>99.9	2.2	>100 (>450)	>100
	**35**	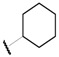	>99.9	2.7	>100 (>423)	>100
	**36**	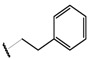	>99.9	2.4	89.1 (345)	100
	**copper oxychloride**				43.1	-
	**Kanamycin**				19.9 (40)	-

MIC—minimal inhibitory concentration; MBC—minimal bactericidal concentration.

**Table 2:** IC_50_ value of compounds **4**, **16** and **28** in μM against cell lines.

Compounds	HaCaT	Huh-7,5	MRC-5	VERO	ACHN
**4**	64.86 ± 8.91 ^a^	70.63 ± 5.52 ^a^	127.45 ± 4.59 ^b,c,h^	111.4 ± 8.34 ^c,f,h^	152.9 ± 7.07 ^d^
**16**	69.81 ± 4.87 ^a^	20.60 ± 0.23 ^e^	144.8 ± 6.29 ^b,d^	93.55 ± 1.93 ^f^	140.35 ± 7.56 ^b,d^
**28**	74.02 ± 11.74 ^a,g^	64.84 ± 14.91 ^a^	128.7 ± 2.12 ^b,c,h^	92.31 ± 9.34 ^f,g^	114.05 ± 9.97 ^g^
**Kanamycin**	>200 ^i^	>200 ^i^	>200 ^i^	>200 ^i^	>200 ^i^
**copper oxychloride**	>200 ^i^	>200 ^i^	>200 ^i^	>200 ^i^	>200 ^i^

Different letters indicate statistical significance *p* < 0.05 in Tukey’s multiple comparisons test.

## Supplementary Materials

Supplementary materials can be found at http://www.mdpi.com/1422-0067/19/10/3050/s1.
