# Supplementary material for: Design of Antibacterial Agents: Alkyl Dihydroxybenzoates against Xanthomonas citri subsp. citri
Source: Int J Mol Sci. 2018 Oct 6;19(10):3050. doi: 10.3390/ijms19103050 (PMC6213047; doi:10.3390/ijms19103050)

Supplementary Materials

Design of Antibacterial Agents: Alkyl Dihydroxybenzoates Against *Xanthomonas citri* subsp. *citri*

Ana Carolina Nazaré[1,*], Carlos Roberto Polaquini[1], Lucia Bonci Cavalca[2], Daiane Bertholin Anselmo[1], Marilia de Freitas Calmon Saiki[3], Diego Alves Monteiro[3], Paula Rahal[3], Eleni Gomes[3], Dirk-Jan Scheffers[4], Henrique Ferreira[2] and Luis Octavio Regasini[1,*]

[1]Department of Chemistry and Environmental Sciences, Institute of Biosciences, Humanities and Exact Sciences, São Paulo State University (UNESP), São José do Rio Preto, SP, Brazil

[2] Department of Biochemistry and Microbiology, Biosciences Institute, São Paulo State University (UNESP), Rio Claro, SP, Brazil

[3]Department of Biology, Institute of Biosciences, Humanities and Exact Sciences, São Paulo State University (UNESP), São José do Rio Preto, SP, Brazil

[4]Department of Molecular Microbiology, Groningen Biomolecular Sciences and Biotechnology Institute, University of Groningen, Groningen, Netherlands

***** Corresponding author Tel.: +55 17 3221-2362

e-mail: acarolnazare@gmail.com (A. C. Nazaré) and luis.regasini@unesp.br (L.O. Regasini)

1. SPECTROSCOPY DATA ANALYSES

The purity of compounds was measured on HPLC-PAD equipment, the analysis were performed for calculations of partition coefficient, using MeOH:H_2O (3:1) as isocratic mobile phase. The NMR spectra were recorded on a Bruker Avance III 14.1 T (600 MHz), Bruker Avance III 9.4 T (400 MHz) and Bruker Fourier 7.1 T (300 MHz) spectrometers, using $CDCl_3$ or DMSO-d_6 as solvent. The chemical shifts (δ) and coupling constants (J) were expressed in ppm and Hz, respectively. Multiplicities were reported as singlet (s), doublet (d), doublet of doublet (dd), triplet (t), quartet (q), and septet (sp).

1.1 ethyl 2,4 dihydroxybenzoate (**1**)

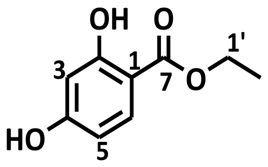

Pale yellow solid

Yield: 20 %

Purity: 87.6 %

^{1}H NMR (400 MHz, $CDCl_3$) δ_H in ppm (multiplicity; J in Hz): 11.09 (s, 2-OH), 7.77 (d, 8.8, H-6), 6.42 (d, 2.4, H-3), 6.39 (dd, 8.8 and 2.4, H-5), 5.92 (s; 4-OH), 4.39 (q, 7.2, H-1’), 1.42 (t, 7.2, H-2’)

^{13}C NMR (100 MHz, $CDCl_3$) δ_C in ppm: 170.0 (C-7), 163.6 (C-4), 162.0 (C-2), 131.9 (C-3), 107.8 (C-5), 106.1 (C-1), 103.1 (C-6), 61.1 (C-1’), 14.2 (C-2’)

1.2 butyl 2,4 dihydroxybenzoate (**2**)

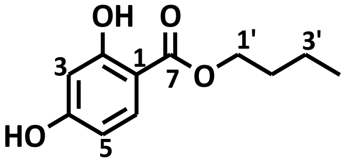

White crystal

Yield: 98 %

Purity: 85.6 %

^{1}H NMR(400 MHz, CDCl$_3$) δ_H in ppm (multiplicity; *J* in Hz): 11.09 (s, 2-OH), 7.76 (d, 8.4, H-6), 6.42 (d, 2.4, H-3), 6.39 (dd, 8.4 and 2.4, H-5), 5.93 (s, 4-OH), 4.34 (t, 6.4, H-1’), 1.81–1.73 (H-2’), 1.54–1.45 (H-3’), 1.00 (t, 7.6, H-4’)

^{13}C NMR (100 MHz, CDCl$_3$) δ_C in ppm:170.1 (C-7), 163.6 (C-4), 162.0 (C-2), 131.9 (C-3), 107.8 (C-5), 106.1 (C-1), 103.1 (C-6), 64.9 (C-1’), 30.6 (C-2’), 19.2 (C-3’), 13.7 (C-4’)

1.3 hexyl 2,4 dihydroxybenzoate (**3**)

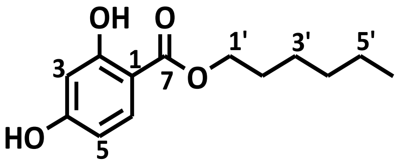

White crystal

Yield: 92 %

Purity: 81.7 %

^{1}H NMR (400 MHz, CDCl$_3$) δ_H in ppm (multiplicity; *J* in Hz): 11.08 (s, 2-OH), 7.76 (d, 8.8, H-6), 6.42 (d, 2.4, H-3), 6.39 (dd, 8.8 and 2.4, H-5), 5.83 (s, 4-OH), 4.33 (t, 6.8, H-1’), 1.80–1.76 (H-2’), 1.47–1.36 (H-3’−H-5’), 0.93 (t, 6.8, H-6’)

^{13}C NMR (100 MHz, CDCl$_3$) δ_C in ppm: 170.1 (C-7), 163.7 (C-4), 162.0 (C-2), 131.9 (C-3), 107.7 (C-5), 106.1 (C-1), 103.1 (C-6), 65.2 (C-1’), 31.4, 28.6, 25.6, and 22.5 (C-2’−C-5’), 14.0 (C-6’)

1.4 heptyl 2,4 dihydroxybenzoate (**4**)

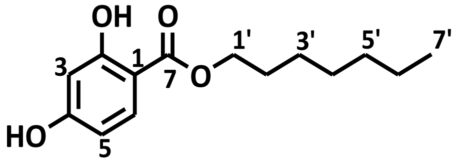

White crystal

Yield: 82 %

Purity: 83.8 %

^{1}H NMR (600 MHz, $CDCl_3$) δ_H in ppm (multiplicity; *J* in Hz): 11.09 (s, 2-OH), 7.76 (d, 9.0, H-6), 6.42 (d, 2.4, H-3), 6.40 (dd, 9.0 and 2.4, H-5), 5.90 (s, 4-OH), 4.32 (t, 6.7, H-1’), 1.80–1.76 (H-2’), 1.47–1.27 (H-3’−H-6’), 0.91 (t, 6.9, CH_3)

^{13}C NMR (150 MHz, $CDCl_3$) δ_C in ppm: 170.1 (C-7), 163.7 (C-4), 161.9 (C-2), 131.9 (C-3), 107.7 (C-5), 106.1 (C-1), 103.1 (C-6), 65.2 (C-1’), 31.7, 28.9, 28.6, 25.9, and 22.6 (C-2’−C-6’), 14.1 (C-7’)

1.5 octyl 2,4 dihydroxybenzoate (**5**)

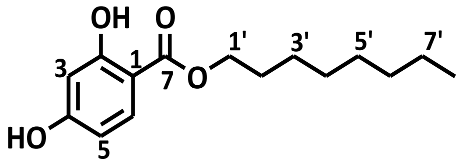

White crystal

Yield: 82 %

Purity: 79.6 %

^{1}H NMR (400 MHz, $CDCl_3$) δ_H in ppm (multiplicity; *J* in Hz): 7.74 (d, 8.4, H-6), 6.40–6.37 (H-3 and H-5), 4.31 (t, 6.4, H-1’), 1.80–1.73 (H-2’), 1.46–1.30 (H-3’–H-7’), 0.91 (t, 6.4, H-8’)

^{13}C NMR (100 MHz, $CDCl_3$) δ_C in ppm: 170.7 (C-7), 163.6 (C-4), 162.2 (C-2), 131.9 (C-3), 107.9 (C-5), 106.0 (C-1), 103.1 (C-6), 65.2 (C-1’), 31.8, 29.2, 29.1, 28.6, 26.0, and 22.6 (C-2’−C-7’), 14.1 (H-8’)

1.6 nonyl 2,4 dihydroxybenzoate (**6**)

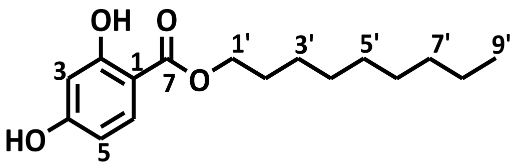

White crystal

Yield: 98 %

Purity: 99.0 %

^{1}H NMR (600 MHz, $CDCl_3$) δ_H in ppm (multiplicity; *J* in Hz): 11.08 (s, 2-OH), 7.76 (d, 8.8, H-6), 6.42 (d, 2.4, H-3), 6.39 (dd, 8.8 and 2.4, H-5), 5.83 (s, 4-OH), 4.35 (t, 6.7, H-1'), 1.81–1.76 (H-2'), 1.47–1.29 (H-3'–H-8'), 0.90 (t, 6.8, H-9')

^{13}C NMR (100 MHz, $CDCl_3$) δ_C in ppm: 170.1 (C-7), 163.6 (C-4), 162.0 (C-2), 131.9 (C-3), 107.8 (C-5), 106.1 (C-1), 103.1 (C-6), 65.2 (C-1'), 31.9, 29.5, 29.2, 28.6, 26.0, and 22.7 (C-2'−C-8'), 14.1 (C-9')

1.7 decyl 2,4 dihydroxybenzoate (**7**)

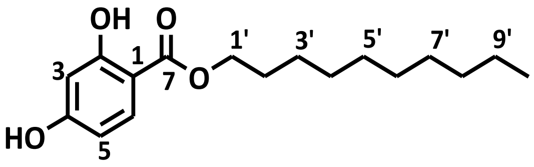

Pale yellow solid

Yield: 95 %

Purity: 99.5 %

^{1}H NMR (400 MHz, $CDCl_3$) δ_H in ppm (multiplicity; *J* in Hz): 11.08 (s, 2-OH), 7.76 (d, 8.4 Hz, H-6), 6.42 (d, 2.4, H-3), 6.39 (dd, 8.4 and 2.4, H-5), 5.65 (s, 4-OH), 4.33 (t, 6.4, H-1'), 1.81–1.74 (H-2'), 1.47–1.29 (H-3'–H-9'), 0.90 (t, 6.8, H-10')

^{13}C NMR (100 MHz, $CDCl_3$) δ_C in ppm: 170.1 (C-7), 163.6 (C-4), 162.0 (C-2), 131.9 (C-3), 107.8 (C-5), 106.1 (C-1), 103.1 (C-6), 65.3 (C-1'), 31.9, 29.5, 29.3, 29.2, 28.6, 26.0, and 22.7 (C-2'−C-9'), 14.1 (C-10')

1.8 dodecyl 2,4 dihydroxybenzoate (**8**)

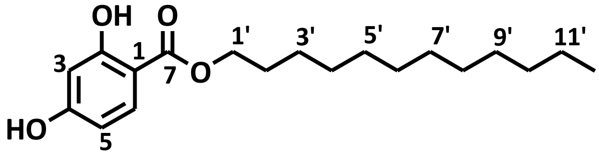

White crystal

Yield: 99 %

Purity: > 99.9 %

^{1}H NMR (300 MHz, $CDCl_3$) δ_H in ppm (multiplicity; *J* in Hz): 11.07 (s, 2-OH), 7.75 (d, 8.4, H-6), 6.41 (d, 2.1, H-3) 6.38 (dd, 8.4 and 2.1, H-5), 5.68 (s, 4-OH), 4.31 (t, 6.6, H-1'), 1.81–1.72 (H-2'), 1.43–1.27 (H-3'–H-11'), 0.89 (t, 6.6, H-12')

^{13}C NMR (100 MHz, $CDCl_3$) δ_C in ppm: 170.1 (C-7), 163.7 (C-4), 162.0 (C-2), 131.8 (C-3), 107.8 (C-5), 106.1 (C-1), 103.1 (C-6), 65.2 (C-1'), 31.9, 29.7, 29.6, 29.5, 29.4, 29.3, 29.2, 28.6, 26.0, and 22.7 (C-2'−C-11'), 14.1 (C-12')

1.9 isopropyl 2,4 dihydroxybenzoate (**9**)

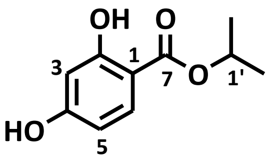

Brown oil

Yield: 68 %

Purity: 93.2 %

^{1}H NMR (600 MHz, $CDCl_3$) δ_H in ppm (multiplicity; *J* in Hz): 11.21 (s, 2-OH), 7.75 (d, 9.0, H-6), 6.42 (d, 2.4, H-3), 6.39 (dd, 9.0 and 2.4, H-5), 5.27 (sp, 6.6, H-1’), 1.39 (d, 6.6, H-2’)

^{13}C NMR (150 MHz, $CDCl_3$) δ_C in ppm: 169.7 (C-7), 163.6 (C-4), 162.0 (C-2), 131.9 (C-3), 107.8 (C-5), 106.3 (C-1), 103.0 (C-6), 68.9 (C-1’), 21.9 (C-2’)

1.10 cyclopentyl 2,4 dihydroxybenzoate (**10**)

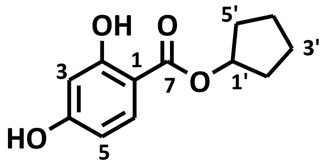

White crystal

Yield: 40 %

Purity: > 99.9 %

^{1}H NMR (600 MHz, $CDCl_3$) δ_H in ppm (multiplicity; *J* in Hz): 11.17 (s, 2-OH), 7.72 (d, 8.4, H-6), 6.41 (d, 2.4, H-3), 6.38 (dd, 8.4 and 2.4, H-5), 5.44– 5.41 (H-1’), 2.03–1.59 (H-2’–H-5’)

^{13}C NMR (150 MHz, $CDCl_3$) δ_C in ppm: 169.5 (C-7), 163.7 (C-4), 161.9 (C-2), 131.9 (C-3), 107.7 (C-5), 106.5 (C-1), 103.1 (C-6), 73.5 (C-1’), 31.6 (C-2’ and C-5’), 23.6 (C-3’ and C-4’)

1.11 cyclohexyl 2,4 dihydroxybenzoate (**11**)

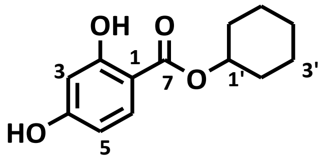

Brown oil

Yield: 92 %

Purity: > 99.9%

^{1}H NMR (600 MHz, $CDCl_3$) δ_H in ppm (multiplicity; *J* in Hz): 11.18 (s, 2-OH), 7.78 (d, 8.4, H-6), 6.42 (d, 2.4, H-3), 6.39 (dd, 8.4 and 2.4, H-5), 5.83 (s, 4-OH), 5.07– 5.02 (H-1’), 1.97–1.34 (H-2’−H-6’)

^{13}C NMR (150 MHz, $CDCl_3$) δ_C in ppm: 169.5 (C-7), 163.7 (C-4), 161.9 (C-2), 131.9 (C-3), 107.7 (C-5), 106.5 (C-1), 103.1 (C-6), 73.5 (C-1’), 31.6 (C-2’ and C-6’), 25.4 (C-4’), 23.6 (C-3’ and C-5’)

1.13 ethyl 2,5 dihydroxybenzoate (**13**)

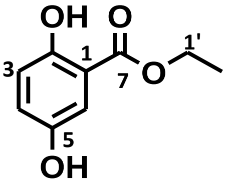

Pale yellow crystal

Yield: 32 %

Purity: 98.3 %

^{1}H NMR (400 MHz, $CDCl_3$) δ_H in ppm (multiplicity; *J* in Hz): 10.44 (s, 2-OH), 7.32 (d, 3.2, H-6), 7.03 (dd, 8.8 and 3.2, H-4), 6.89 (d, 8.8, H-3), 4.41 (q, 7.2, H-1’), 1.42 (t, 7.2, H-2’)

^{13}C NMR (100 MHz, $CDCl_3$) δ_C in ppm: 169.8 (C-7), 155.8 (C-2), 147.7 (C-5), 123.9 (C-4), 118.5 (C-3), 114.8 (C-6), 112.4 (C-1), 61.5 (C-1’), 14.2 (C-2’)

1.14 butyl 2,5 dihydroxybenzoate (**14**)

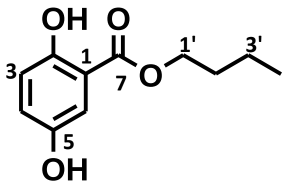

Pale yellow crystal

Yield: 87 %

Purity: 82.0 %

^{1}H NMR (400 MHz, CDCl$_3$) δ$_H$ in ppm (multiplicity; *J* in Hz): 10.44 (s, 2-OH), 7.31 (d, 2.8, H-6), 7.03 (dd, 8.8 and 2.8, H-4), 6.90 (d, 8.8, H-3), 4.90 (s; 5-OH), 4.36 (t, 6.8, H-1’), 1.81−1.74 (H-2’), 1.54−1.45 (H-3’), 1.01 (t, 7.2, H-4’)

^{13}C NMR (100 MHz, CDCl$_3$) δ$_C$ in ppm: 169.8 (C-7), 155.9 (C-2), 147.7 (C-5), 123.9 (C-4), 118.5 (C-3), 114.7 (C-6), 112.5 (C-1), 65.3 (C-1’), 30.6 (C-2’), 19.2 (C-3’), 13.7 (C-4’)

1.15 hexyl 2,5 dihydroxybenzoate (**15**)

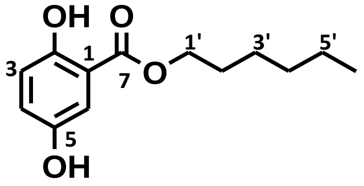

Pale yellow oil

Yield: 67 %

Purity: 80.1 %

^{1}H NMR (400 MHz, CDCl$_3$) δ$_H$ in ppm (multiplicity; *J* in Hz): 10.44 (s, 2-OH), 7.31 (d, 3.2, H-6), 7.03 (dd, 9.2 and 3.2, H-4), 6.89 (d, 9.2, H-3), 4.35 (t, 6.8, H-1’), 1.83−1.75 (H-2’), 1.48−1.36 (H-3’–H-5’), 0.93 (t, 6.8, H-6’)

^{13}C NMR (100 MHz, $CDCl_3$) δ_C in ppm: 169.8 (C-7), 155.9 (C-2), 147.7 (C-5), 123.9 (C-4), 118.5 (C-3), 114.7 (C-6), 112.5 (C-1), 65.6 (C-1’), 31.4, 28.5, 25.6, and 22.5 (C-2’–C-5’), 14.0 (C-6’)

1.16 heptyl 2,5 dihydroxybenzoate (**16**)

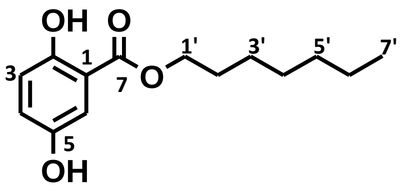

Pale yellow crystal

Yield: 40 %

Purity: 99.3 %

^{1}H NMR (600 MHz, $CDCl_3$) δ_H in ppm (multiplicity; *J* in Hz): 10.45 (s, 2-OH), 7.31 (d, 3.0, H-6), 7.03 (dd, 9.0 and 3.0, H-4), 6.90 (d, 9.0, H-3), 4.35 (t, 6.7, H-1’), 1.81–1.77 (H-2’), 1.48–1.27 (H-3’–H-6’), 0.92 (t, 6.9, H-7’)

^{13}C NMR (150 MHz, $CDCl_3$) δ_C in ppm: 169.8 (C-7), 155.9 (C-2), 147.6 (C-5), 123.9 (C-4), 118.5 (C-3), 114.7 (C-6), 112.5 (C-1), 65.6 (C-1’), 31.7, 28.9, 28.6, 25.9, and 22.6 (C-2’–C6’), 14.1 (C-7’)

1.17 octyl 2,5 dihydroxybenzoate (**17**)

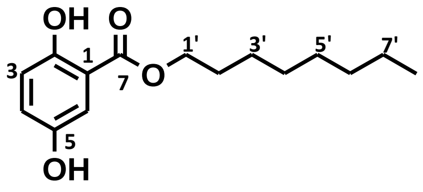

Pale yellow crystal

Yield: 95 %

Purity: 79.8 %

^{1}H NMR (400 MHz, $CDCl_3$) δ_H in ppm (multiplicity; *J* in Hz): 10.45 (s, 2-OH), 7.31 (d, 3.2, H-6), 7.03 (dd, 9.2 and 3.2, H-4), 6.90 (d, 9.2, H-3), 4.35 (t, 6.8, H-1’), 1.82−1.75 (H-2’), 1.49−1.31 (H-3’–H-7’), 0.91 (t, 6.8, H-8’)

^{13}C NMR (100 MHz, $CDCl_3$) δ_C in ppm: 169.8 (C-7), 155.9 (C-2), 147.7 (C-5), 123.9 (C-4), 118.5 (C-3), 114.8 (C-6), 112.5 (C-1), 65.7 (C-1’), 31.8, 29.2, 29.1, 28.5, 26.0, and 22.6 (C-2’–C7’), 14.1 (C-8’)

1.18 nonyl 2,5 dihydroxybenzoate (**18**)

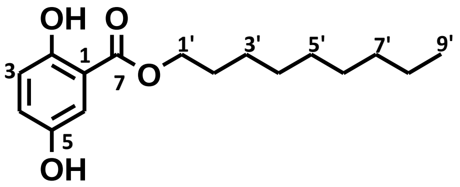

Pale yellow crystal

Yield: 99 %

Purity: > 99.9 %

^{1}H NMR (600 MHz, $CDCl_3$) δ_H in ppm (multiplicity; *J* in Hz): 10.46 (s, 2-OH), 7.31 (d, 3.0, H-6), 7.03 (dd, 9.0 and 3.0, H-4), 6.90 (d, 9.0, H-3), 4.35 (t, 6.7, H-1’), 1.81–1.76 (H-2’), 1.47–1.29 (H-3’–H-8’), 0.90 (t, 6.8, H-9’)

^{13}C NMR (150 MHz, $CDCl_3$) δ_C in ppm: 169.9 (C-7), 155.8 (C-2), 147.7 (C-5), 123.9 (C-4), 118.5 (C-3), 114.7 (C-6), 112.5 (C-1), 65.7 (C-1’), 31.9, 29.5, 29.3, 28.5, 26.0, and 22.7 (C-2’–C-8’), 14.1 (C-9’)

1.19 decyl 2,5 dihydroxybenzoate (**19**)

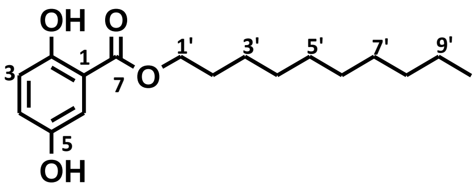

Pale yellow crystal

Yield: 70 %

Purity: 76.5 %

^{1}H NMR (400 MHz, $CDCl_3$) δ_H in ppm (multiplicity; *J* in Hz): 10.44 (s, 2-OH), 7.31 (d, 3.2, H-6), 7.02 (dd, 9.2 and 3.2, H-4), 6.90 (d, 9.2, H-3), 4.84 (s, 5-OH), 4.35 (t, 6.4, H-1'), 1.82−1.75 (H-2'), 1.47−1.30 (H-3'–H-9'), 0.90 (t, 6.8, H-10')

^{13}C NMR (100 MHz, $CDCl_3$) δ_C in ppm: 169.8 (C-7), 155.9 (C-2), 147.7 (C-5), 123.9 (C-4), 118.5 (C-3), 114.8 (C-6), 112.5 (C-1), 65.6 (C-1'), 31.9, 29.6, 29.5, 29.3, 29.2, 28.5, 26.0, and 22.7 (C-2'–C-9'), 14.1 (C-10')

1.20 dodecyl 2,5 dihydroxybenzoate (**20**)

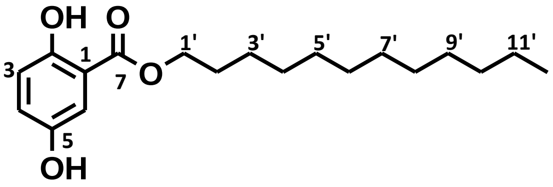

Pale yellow crystal

Yield: 99 %

Purity: > 99.9 %

^{1}H NMR (300 MHz, $CDCl_3$) δ_H in ppm (multiplicity; *J* in Hz): 10.43 (s, 2-OH), 7.30 (d, 3.3, H-6), 7.02 (dd, 8.7 and 3.3, H-4), 6.89 (d, 8.7, H-3), 4.34 (t, 6.6, H-1'), 1.82−1.73 (H-2'), 1.42−1.27 (H-3'–H-11'), 0.89 (t, 6.3, H-12')

^{13}C NMR (100 MHz, $CDCl_3$) δ_C in ppm: 169.8 (C-7), 155.9 (C-2), 147.7 (C-5), 123.9 (C-4), 118.5 (C-3), 114.7 (C-6), 112.5 (C-1), 65.6 (C-1'), 31.9, 29.7, 29.6, 29.5, 29.3, 29.2, 28.6, 26.0, and 22.7 (C-2'–C-11'), 14.1 (C-12')

1.21 isopropyl 2,5 dihydroxybenzoate (**21**)

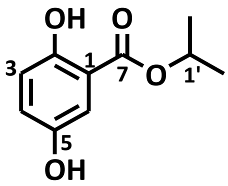

Pale yellow solid

Yield: 80 %

Purity: > 99.9 %

^{1}H NMR (400 MHz, $CDCl_3$) δ_H in ppm (multiplicity; *J* in Hz): 10.51 (s, 2-OH), 7.31 (d, 3.2, H-6), 7.02 (dd, 9.2 and 3.2, H-4), 6.89 (d, 9.2, H-3), 5.29 (sp, 6.4, H-1'), 1.40 (d, 6.4, H-2')

^{13}C NMR (150 MHz, $CDCl_3$) δ_C in ppm: 169.3 (C-7), 155.9 (C-2), 147.6 (C-5), 123.8 (C-4), 118.4 (C-3), 114.8 (C-6), 112.8 (C-1), 69.4 (C-1'), 21.8 (C-2')

1.22 cyclopentyl 2,5 dihydroxybenzoate (**22**)

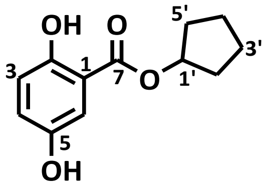

White solid

Yield: 75 %

Purity: > 99.9 %

^{1}H NMR (300 MHz, $CDCl_3$) δ_H in ppm (multiplicity; *J* in Hz): 10.50 (s, 2-OH), 7.27 (d, 3.0, H-6), 7.00 (dd, 9.0 and 3.0, H-4), 6.88 (d, 9.0, H-3), 5.45–5.41 (H-1'), 4.76 (s, 5-OH), 1.97–1.67 (H-2'–H-5')

^{13}C NMR (150 MHz, $CDCl_3$) δ_C in ppm: 169.5 (C-7), 155.9 (C-2), 147.5 (C-5), 123.7 (C-4), 118.4 (C-3), 114.8 (C-6), 112.8 (C-1), 78.6 (C-1'), 32.7 (C-2' and C-5'), 23.8 (C-3' and C-4')

1.23 cyclohexyl 2,5 dihydroxybenzoate (**23**)

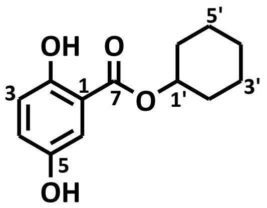

Pale yellow solid

Yield: 75 %

Purity: > 99.9 %

^{1}H NMR (300 MHz, $CDCl_3$) δ_H in ppm (multiplicity; *J* in Hz): 10.51 (s, 2-OH), 7.32 (d, 3.0, H-6), 7.01 (dd, 9.0 and 3.0, H-4), 6.88 (d, 9.0, H-3), 5.10–5.02 (H-1’), 1.93–1.26 (H-2’–H-6’)

^{13}C NMR (150 MHz, $CDCl_3$) δ_C in ppm: 169.2 (C-7), 155.9 (C-2), 147.6 (C-5), 123.8 (C-4), 118.4 (C-3), 114.8 (C-6), 112.9 (C-1), 74.0 (C-1’), 31.5 (C-2’ and C-6’), 25.3 (C-3’ and C-5’), 23.5 (C-4’)

1.24 phenylethyl 2,5 dihydroxybenzoate (**24**)

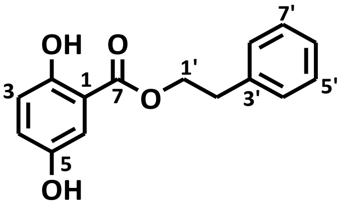

Pale yellow oil

Yield: 7 %

Purity: > 99.9 %

^{1}H NMR (400 MHz, $CDCl_3$) δ_H in ppm (multiplicity; *J* in Hz): 10.33 (s, 2-OH), 7.38−7.28 (H-4’−H-8’), 7.26 (d, 3.2, H-6), 7.02 (dd, 8.8 and 3.2, H-4), 6.90 (d, 8.8, H-3), 4.69 (s, 5-OH), 4.55 (t, 6.8, H-1’), 3.12 (t, 6.8, H-2’)

^{13}C NMR (150 MHz, $CDCl_3$) δ_C in ppm: 169.6 (C-7), 156.0 (C-2), 147.6 (C-5), 137. 4 (C-3’), 128,9 (C-5’ and C-7’), 128.7 (C-4’ and C-8’), 126.8 (C-6’), 124.1 (C-4), 118.5 (C-3), 114.7 (C-6), 112.2 (C-1), 65.9 (C-1’), 35,0 (C-2’)

1.25 ethyl 3,4 dihydroxybenzoate (**25**)

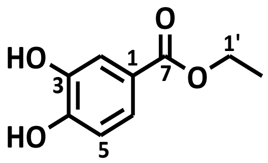

Pale yellow solid

Yield: 90 %

Purity: 99.1 %

^{1}H NMR (400 MHz, DMSO-d_6) δ_H in ppm (multiplicity; *J* in Hz): 7.35 (d, 2.4, H-2), 7.31 (dd, 8.4 and 2.4, H-6), 6.80 (d, 8.4, H-5), 4.31 (q, 7.2, H-1’), 1.27 (t, 7.2, H-2’)

^{13}C NMR (100 MHz, DMSO-d_6) δ_C in ppm: 166.2 (C-7), 150.8 (C-4), 145.5 (C-3), 122.2 (C-6), 121.2 (C-1), 116.7 (C-5), 115.7 (C-2), 60.5 (C-1’), 14.7 (C-2’)

1.26 butyl 3,4 dihydroxybenzoate (**26**)

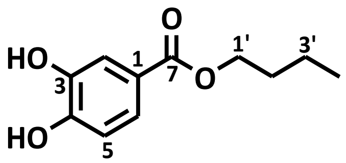

Pale yellow solid

Yield: 98 %

Purity: 99.1 %

^{1}H NMR (400 MHz, $CDCl_3$) δ_H in ppm (multiplicity; *J* in Hz): 7.67 (H-2), 7.58 (dd, 8.4 and 1.6, H-6), 6.93 (d, 8.4, H-5), 4.31 (t, 6.8, H-1’), 1.79−172 (H-2’), 1.53−1.44 (H-3’), 0.99 (t, 5.4, H-4’)

^{13}C NMR (100 MHz, $CDCl_3$) δ_C in ppm: 167.1 (C-7), 148.9 (C-4), 143.1 (C-3), 123.7 (C-6), 122.7 (C-1), 116.6 (C-5), 114.8 (C-2), 65.0 (C-1’), 30.7 (C-2’), 19.3 (C-3’), 13.8 (C-4’)

1.27 hexyl 3,4 dihydroxybenzoate (**27**)

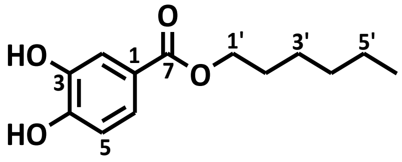

Pale yellow crystal

Yield: 87 %

Purity: 99.1 %

^1H NMR (400 MHz, DMSO-d_6) δ_H in ppm (multiplicity; *J* in Hz): 7.35 (d, 2.4, H-2), 7.31 (dd, 2.4 and 8.0, H-5), 6.81 (d, 8.0, H-6), 4.18 (t, 6.4, H-1’), 1.70−1.63 (H-2’), 1.39–1.30 (H-3’–H-5’), 0.87 (t, 7.2, H-6’)

^{13}C NMR (100 MHz, DMSO-d_6) δ_C in ppm: 166.2 (C-7), 150.9 (C-4), 145.5 (C-3), 122.2 (C-6), 121.1 (C-1), 116.6 (C-5), 115.7 (C-2), 64.4 (C-1’), 31.4, 28.7, 25.6, and 22.5 (C-2’–C-5’), 14.4 (C-6’)

1.28 heptyl 3,4 dihydroxybenzoate (**28**)

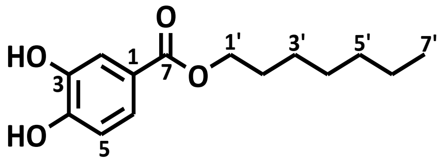

White crystal

Yield: 96 %

Purity: 98.5 %

^{1}H NMR (600 MHz, DMSO-d_6) δ_H in ppm (multiplicity; *J* in Hz): 9.80 (s, 4-OH), 9.39 (s, 3-OH), 7.35 (d, 1.8, H-2), 7.31 (dd, 8.4 and 1.8, H-6), 6.81 (d, 8.4, H-5), 4.17 (t, 6.5, H-1'), 1.68–1.63, (H-2'), 1.38–1.26 (H-3'−H-6'), 0.86 (t, 6.9, H-7')

^{13}C NMR (150 MHz, DMSO-d_6) δ_C in ppm: 166.2 (C-7), 150.8 (C-4), 145.5 (C-3), 122.2 (C-6), 121.2 (C-1), 116.6 (C-5), 115.8 (C-2), 64.5 (C-1'), 31.7, 28.8, 28.7, 26.0, and 22.5 (C-2'–C-6'), 14.4 (C-7')

1.29 octyl 3,4 dihydroxybenzoate (**29**)

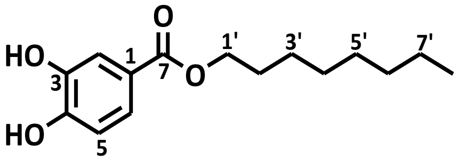

White crystal

Yield: 76 %

Purity: 98.5 %

^{1}H NMR (400 MHz, $CDCl_3$) δ_H in ppm (multiplicity; *J* in Hz): 7.70 (d, 2.0, H-2), 7.58 (dd, 8.4 and 2.0, H-6), 6.93 (d, 8.4, H-5), 4.30 (t, 6.8, H-1'), 1.80−1.73 (H-2'), 1.48–1.30 (H-3'–H-7'), 0.90 (t, 6.8, H-8')

^{13}C NMR (100 MHz, $CDCl_3$) δ_C in ppm: 167.3 (C-7), 148.9 (C-4), 143.2 (C-3), 123.7 (C-6), 122.6 (C-1), 116.7 (C-5), 114.8 (C-2), 65.4 (C-1'), 31.8, 29.2, 29.1, 28.7, 26.0, and 22.6 (C-2'–C-7'), 14.1 (C-8')

1.30 nonyl 3,4 dihydroxybenzoate (**30**)

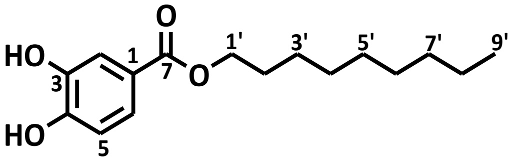

White solid

Yield: 98 %

Purity: 97.9 %

^{1}H NMR (300 MHz, $CDCl_3$) δ_H in ppm (multiplicity; *J* in Hz): 7.67 (d, 2.1, H-2), 7.58 (dd, 8.4 and 2.1, H-6), 6.92 (d, 8.4, H-5), 6.42 (s, 4-OH), 6.15 (s, 3-OH), 4.29 (t, 6.7, H-1'), 1.78–1.71 (H-2'), 1.44–1.28 (H-3'–H-8'), 0.89 (t, 6.7, H-9')

^{13}C NMR (150 MHz, DMSO-d_6) δ_C in ppm: 166.2 (C-7), 150.8 (C-4), 145.5 (C-3), 122.2 (C-6), 121.2 (C-1), 116.6 (C-5), 115.7 (C-2), 64.5 (C-1'), 31.7, 29.4, 29.2, 29.1, 28.7, 26.0, and 22.6 (C-2'–C-8'), 14.1 (C-9')

1.31 decyl 3,4 dihydroxybenzoate (**31**)

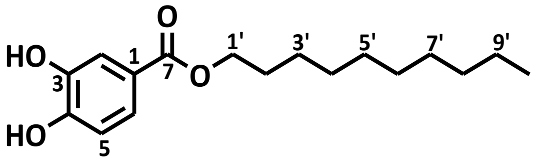

White solid

Yield: 95 %

Purity: 91.3 %

^{1}H NMR (400 MHz, $CDCl_3$) δ_H in ppm (multiplicity; *J* in Hz): 7.68–7.67 (H-2), 7.59 (dd, 8.4 and 1.6, H-6), 6.93 (d, 8.4, H-5), 4.30 (t, 6.8, H-1'), 1.80−1.73 (H-2'), 1.48–1.29 (H-3'–H-9'), 0.90 (t, 6.8, H-10')

^{13}C NMR (100 MHz, $CDCl_3$) δ_C in ppm: 166.9 (C-7), 148.8 (C-4), 143.1 (C-3), 123.7 (C-6), 121.5 (C-1), 116.6 (C-5), 114.8 (C-2), 65.3 (C-1'), 31.9, 29.5, 29.3, 28.7, 26.0, and 22.7 (C-2'–C-9'), 14.1 (C-10')

1.32 dodecyl 3,4 dihydroxybenzoate (**32**)

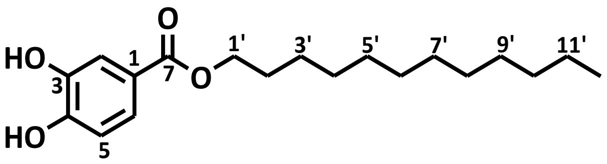

White solid

Yield: 82 %

Purity: > 99.9 %

^{1}H NMR (300 MHz, CDCl$_3$) δ$_H$ in ppm (multiplicity; *J* in Hz): 9.78 (s, 4-OH), 9.36 (s, 3-OH), 7.34 (d, 2.1, H-2), 7.29 (dd, 8.1 and 2.1, H-6), 6.79 (d, 8.1, H-5), 4.16 (t, 6.3, H-1'), 1.67−1.60 (H-2'), 1.38−1.23 (H-3'–H-11'), 0.84 (t, 6.3, H-12')

^{13}C NMR (100 MHz, CDCl$_3$) δ$_C$ in ppm: 166.5 (C-7), 152.9 (C-4), 148.6 (C-3), 123.5 (C-6), 123.2 (C-1), 112.0 (C-5), 110.2 (C-2), 65.0 (C-1'), 31.9, 29.7, 29.6, 29.5, 29.3, 29.2, 28.8, 26.1, and 22.7 (C-2'–C-11'), 14.1 (C-12')

1.33 isopropyl 3,4 dihydroxybenzoate (**33**)

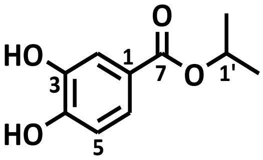

White solid

Yield: 56 %

Purity: > 99.9 %

^{1}H NMR (300 MHz, DMSO-*d_6*) δ$_H$ in ppm (multiplicity; *J* in Hz): 9.75 (s, 4-OH), 9.34 (s, 3-OH), 7.34 (d, 2.1, H-2), 7.28 (dd, 8.4 and 2.1, H-6), 6.79 (d, 8.4, H-5), 5.04 (h, 6.3, H-1'), 1.26 (d, 6.3, H-2')

^{13}C NMR (150 MHz, DMSO-*d_6*) δ$_C$ in ppm: 165.6 (C-7), 150.7 (C-4), 145.5 (C-3), 122.1 (C-6), 121.6 (C-1), 116.6 (C-5), 115.7 (C-2), 67.7 (C-1') 22.2 (C-2')

1.34 cyclopentyl 3,4 dihydroxybenzoate (**34**)

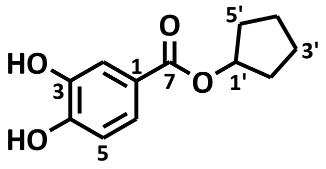

White solid

Yield: 68 %

Purity: > 99.9 %

^{1}H NMR (300 MHz, DMSO-*d*$_6$) δ_H in ppm (multiplicity; *J* in Hz): 9.75 (s, 4-OH), 9.34 (s, 3-OH), 7.32 (d, 2.1, H-2), 7.27 (dd, 8.1 and 2.1, H-6), 6.78 (d, 8.1, H-5), 5.23–5.20 (H-1’), 1.89–1.59 (H-2’–H-5’)

^{13}C NMR (150 MHz, DMSO-*d*$_6$) δ_C in ppm: 165.9 (C-7), 150.7 (C-4), 145.4 (C-3), 122.1 (C-6), 121.6 (C-1), 116.6 (C-5), 115.7 (C-2), 76.9 (C-1’), 32.8 (C-2’ and C-5’), 23.8 (C-3’ and C-4’)

1.35 cyclohexyl 3,4 dihydroxybenzoate (**35**)

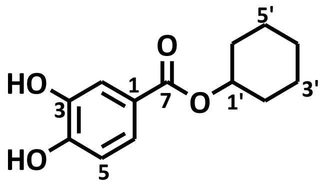

White crystal

Yield: 74 %

Purity: > 99.9 %

^{1}H NMR (600 MHz, DMSO-*d*$_6$) δ_H in ppm (multiplicity; *J* in Hz): 7.36 (d, 1.8, H-2), 7.31 (dd, 8.4 and 1.8, H-6), 6.80 (d, 8.4, H-5), 4.86–4.82 (H-1’), 1.52–1.30 (H-2’–H-6’)

^{13}C NMR (150 MHz, DMSO-d_6) δ_C in ppm: 165.5 (C-7), 150.7 (C-4), 145.5 (C-3), 122.2 (C-6), 121.6 (C-1), 116.7 (C-5), 115.7 (C-2), 72.1 (C-1'), 31.6 (C-2' and C-6'), 25.4 (C-4'), 23.5 (C-3' and C-5')

1.36 phenylethyl 3,4 dihydroxybenzoate (**36**)

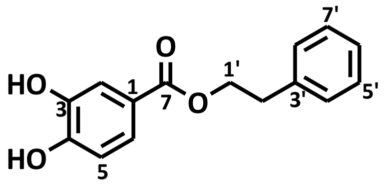

White crystal

Yield: 21 %

Purity: > 99.9 %

^{1}H NMR (300 MHz, DMSO-d_6) δ_H in ppm (multiplicity; *J* in Hz): 7.32–7.18 (H-2, H-6 and H-4'–H-8'), 6.78 (d, 8.4, H-5), 4.37 (t, 6.6, H-1'), 2.98 (t, 6.6 Hz, H-2')

^{13}C NMR (150 MHz, DMSO-d_6) δ_C in ppm: 166.1 (C-7), 150.9 (C-4), 145.5 (C-3), 138.7 (C-3'), 129.4 (C-5' and C-7'), 128.8 (C-4' and C-8'), 126.8 (C-6'), 122.2 (C-6), 121.0 (C-1), 116.7 (C-5), 115.7 (C-2), 65.2 (C-1'), 35.0 (C-2').

Figure S1. I) ^{1}H NMR spectrum of compound **1** (400 MHz – $CDCl_3$)

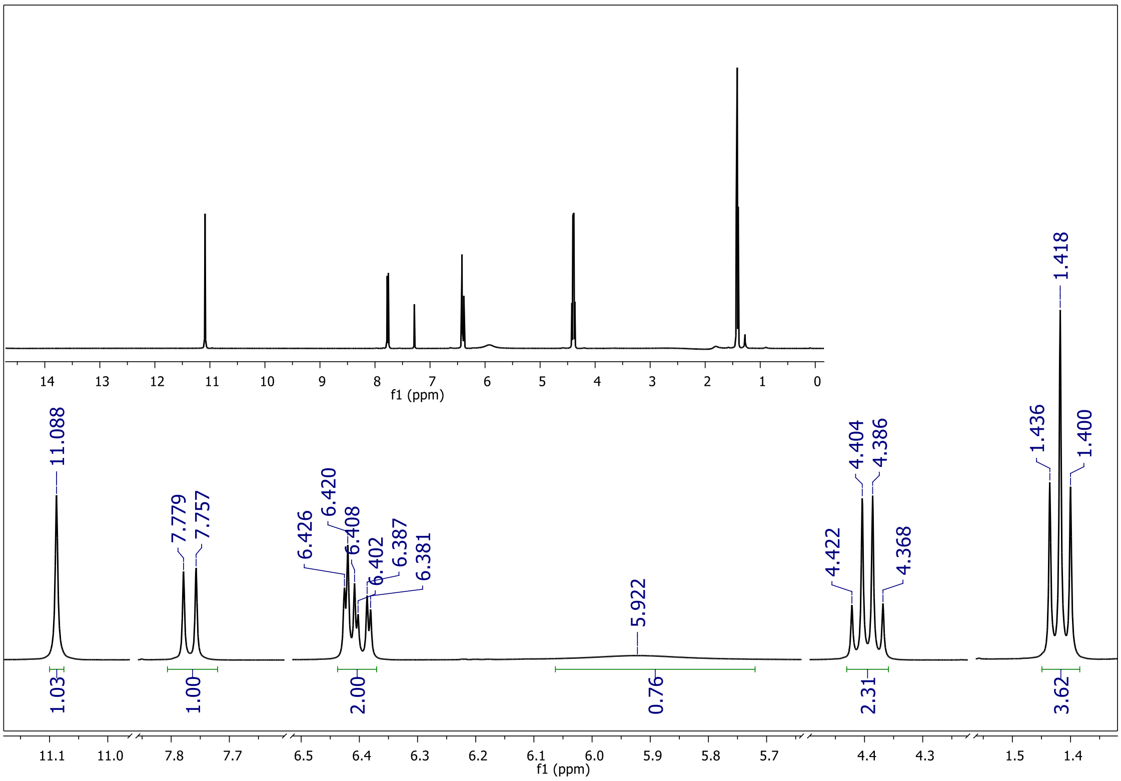

Figure S1. II) ^{13}C NMR spectrum of compound **1** (100 MHz – $CDCl_3$)

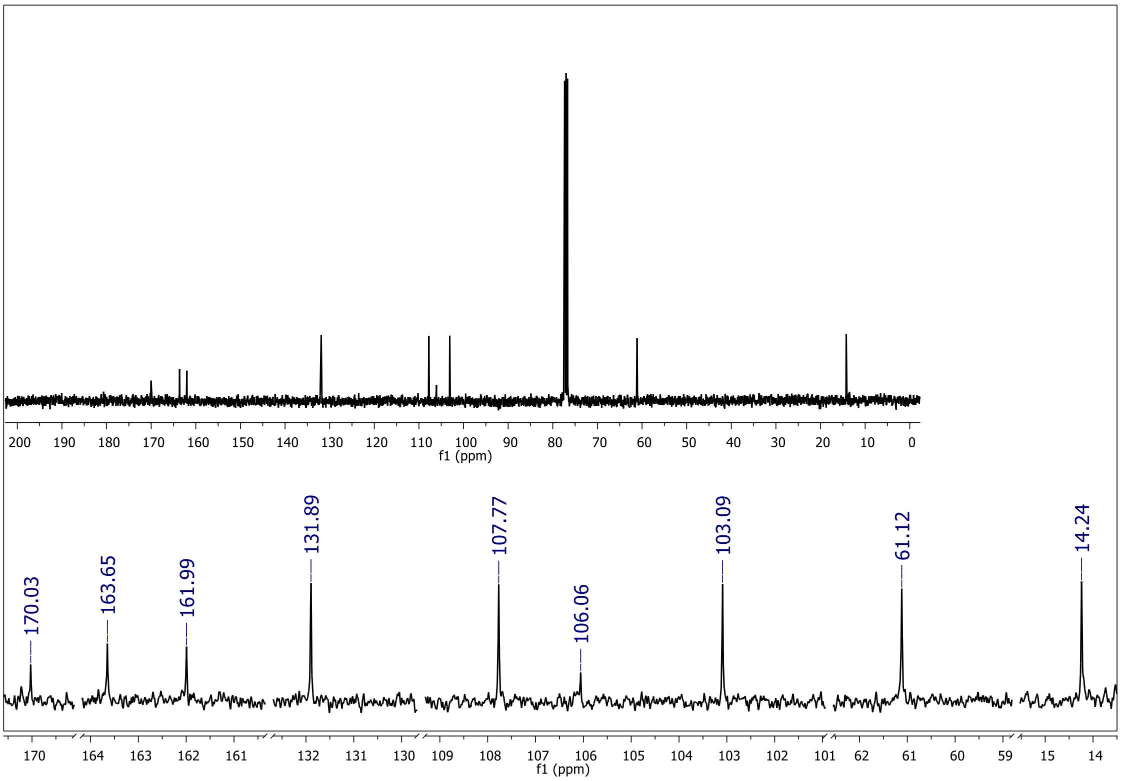

Figure S1. III) HPLC chromatogram of compound **1**

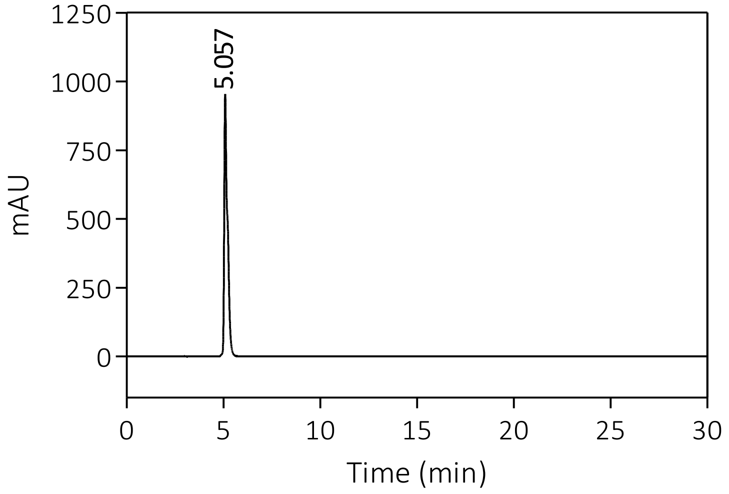

Figure S2. I) ^{1}H NMR spectrum of compound **2** (400 MHz – $CDCl_3$)

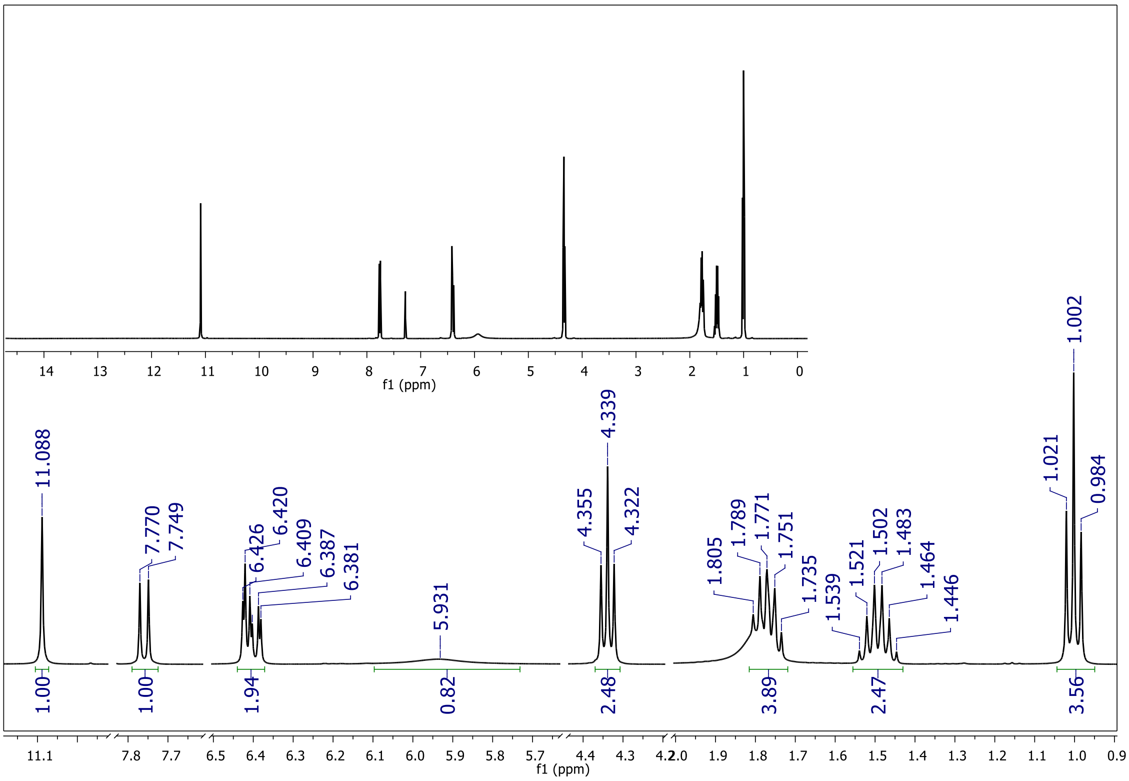

Figure S2. II) ^{13}C NMR spectrum of compound **2** (100 MHz – $CDCl_3$)

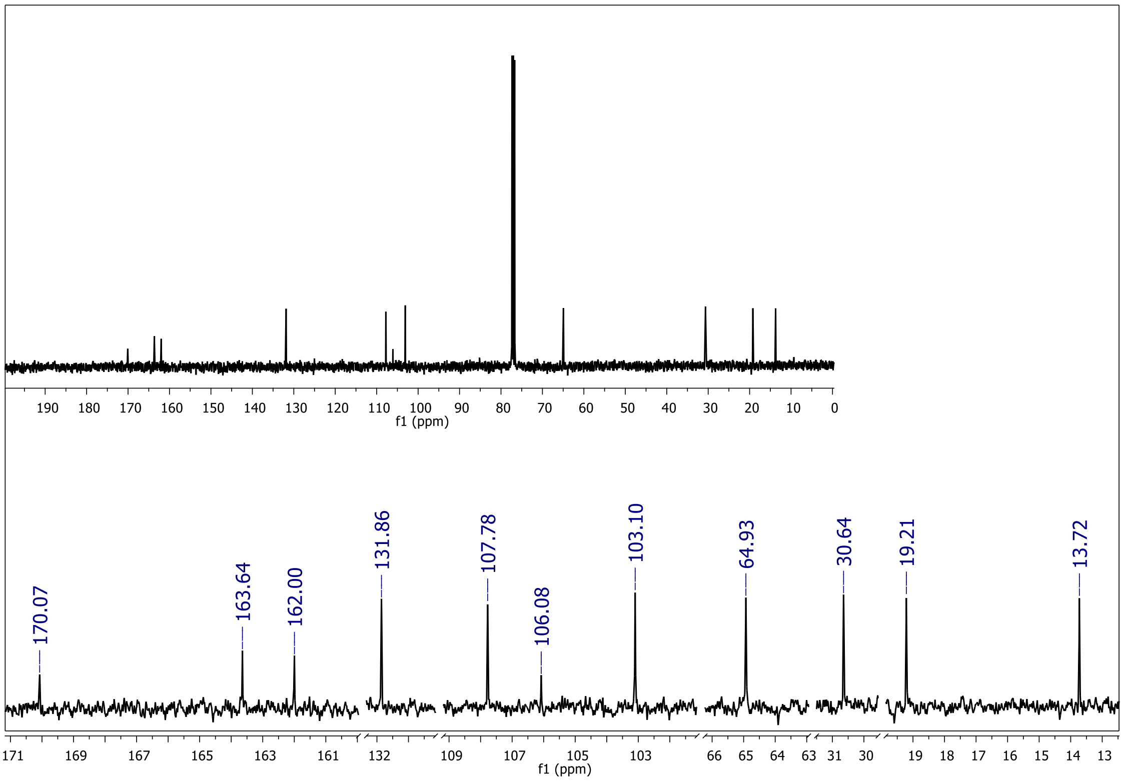

Figure S2. III) HPLC chromatogram of compound **2**

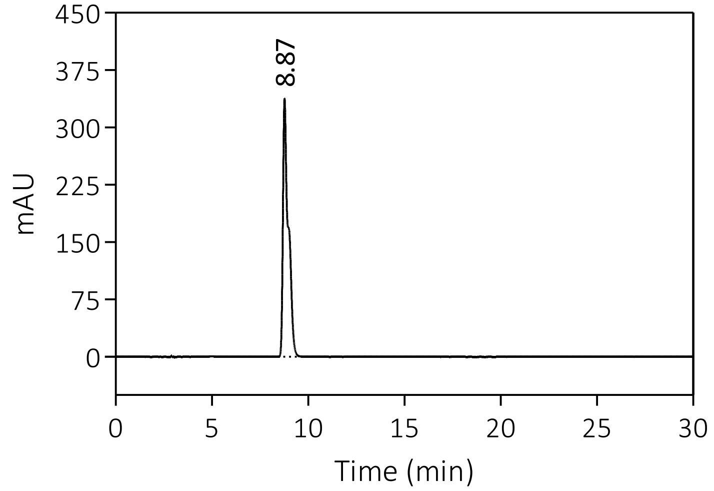

Figure S3. I) ^{1}H NMR spectrum of compound **3** (400 MHz – $CDCl_3$)

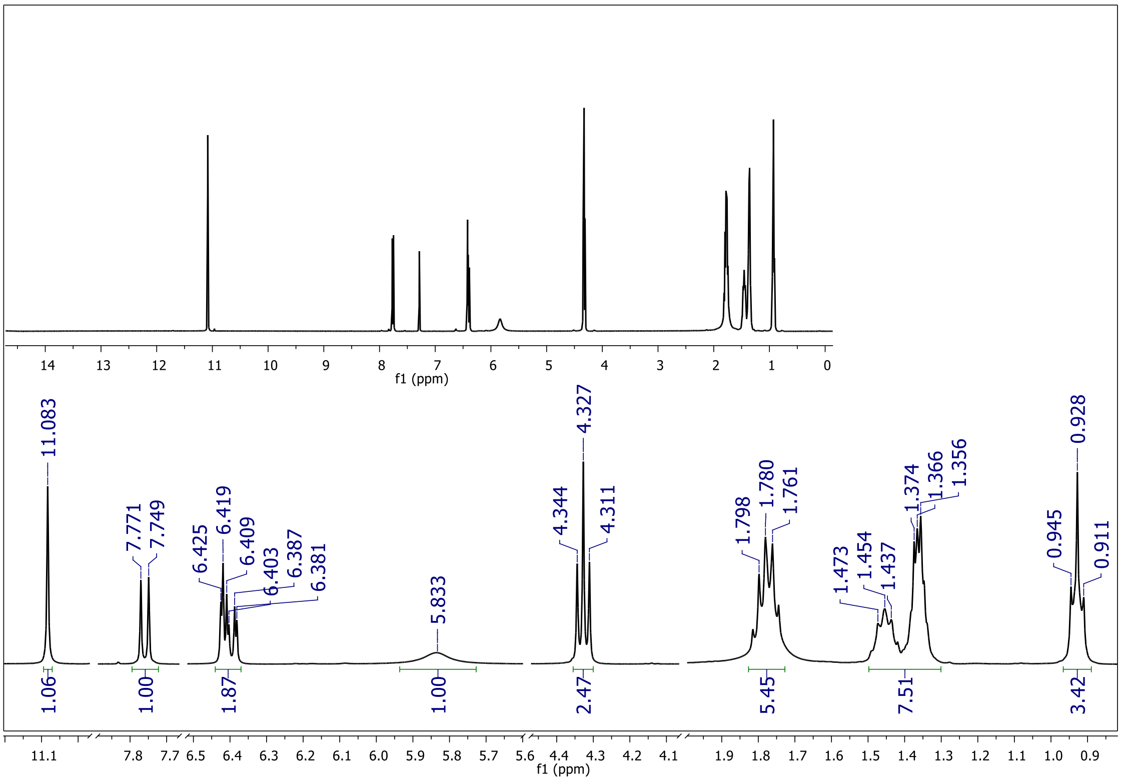

Figure S3. II) ^{13}C NMR spectrum of compound **3** (100 MHz – $CDCl_3$)

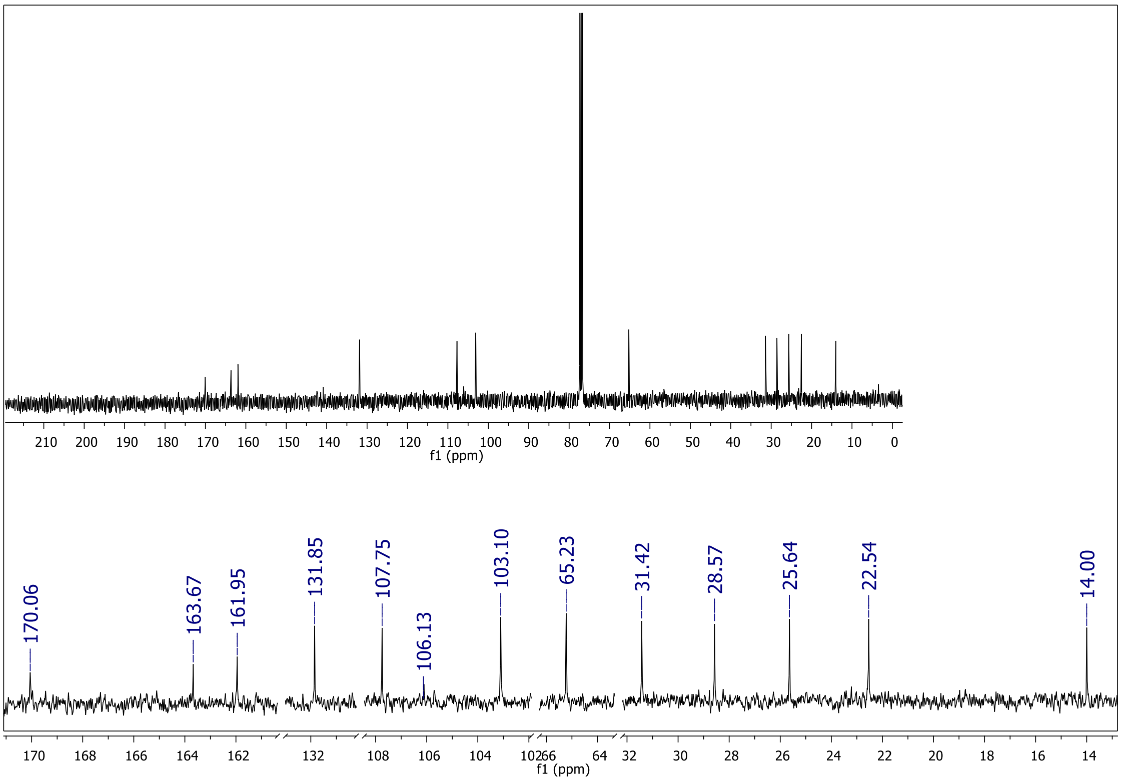

Figure S3. III) HPLC chromatogram of compound **3**

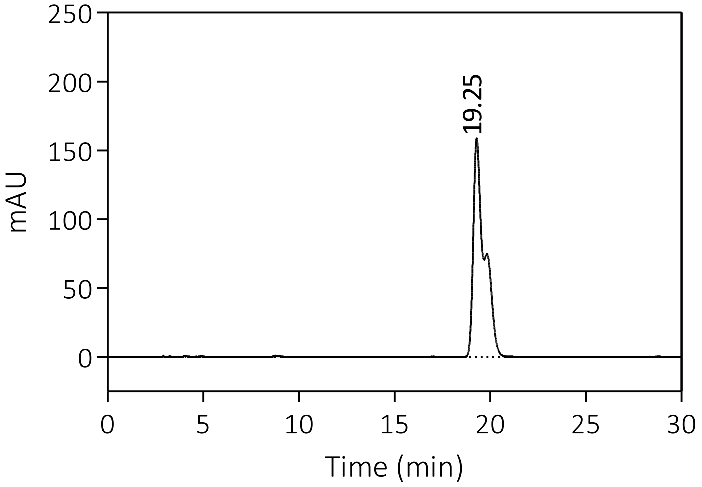

Figure S4. I) ^{1}H NMR spectrum of compound **4** (600 MHz – $CDCl_3$)

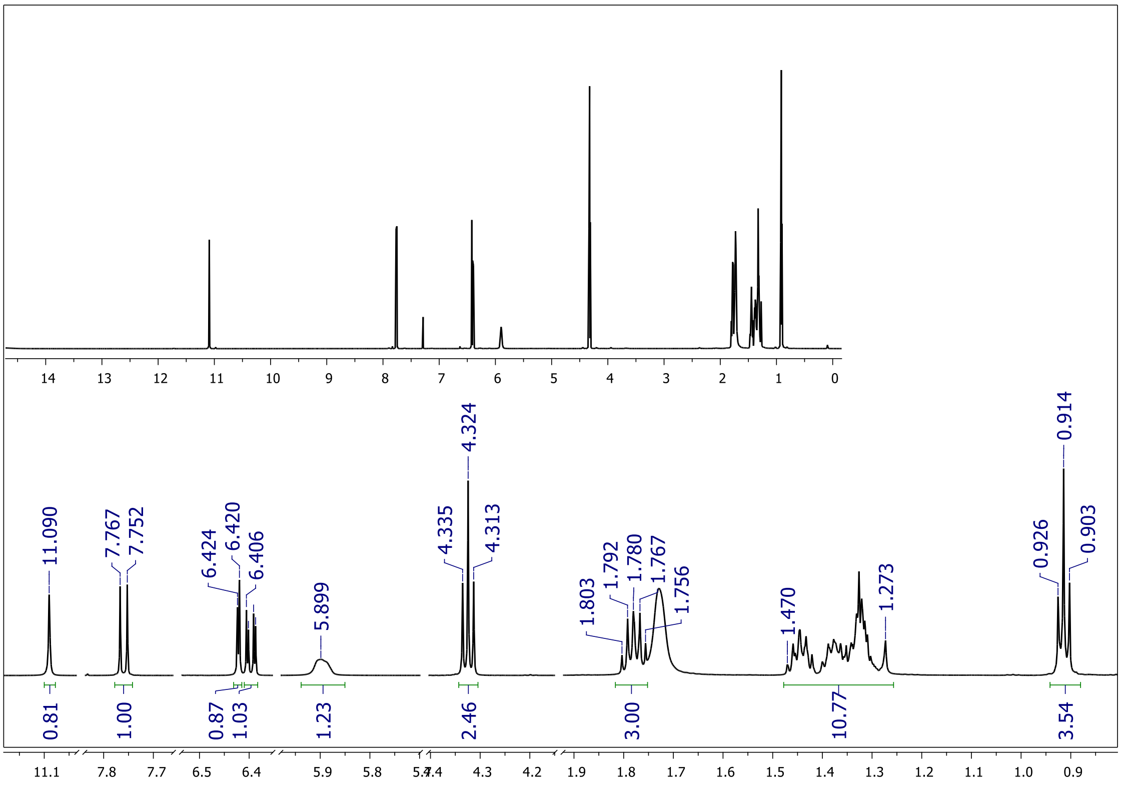

Figure S4. II) ^{13}C NMR spectrum of compound **4** (150 MHz – $CDCl_3$)

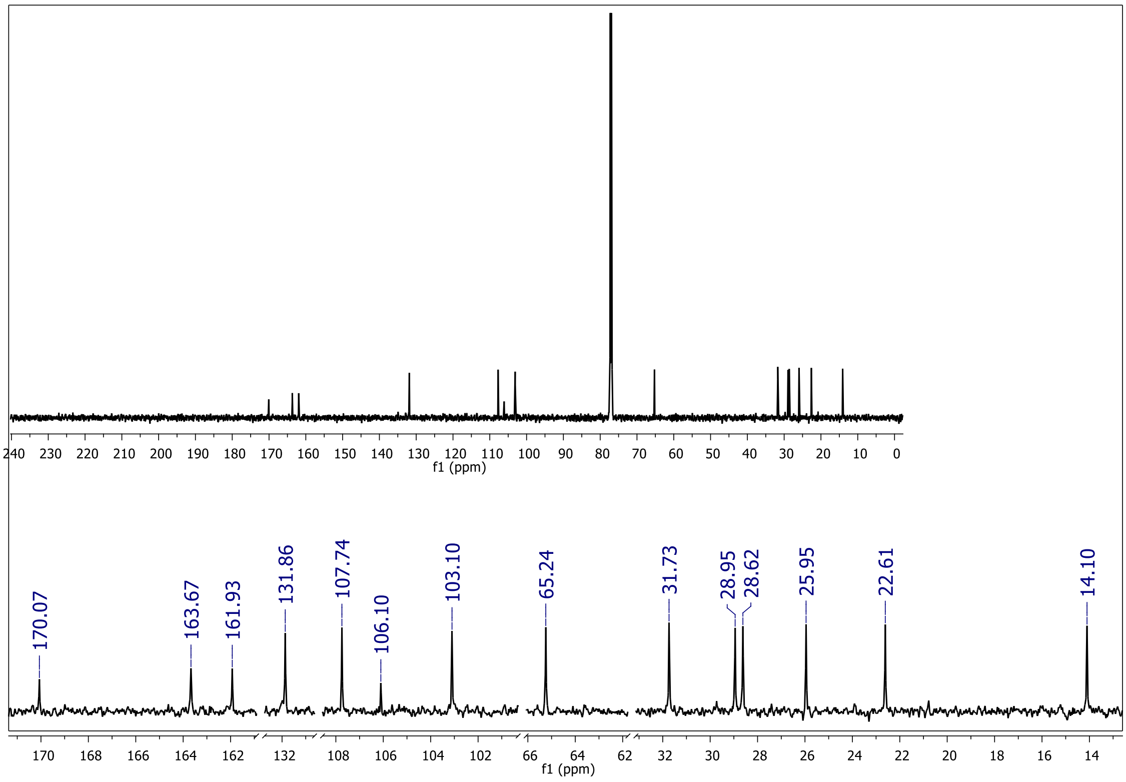

Figure S4. III) HPLC chromatogram of compound **4**

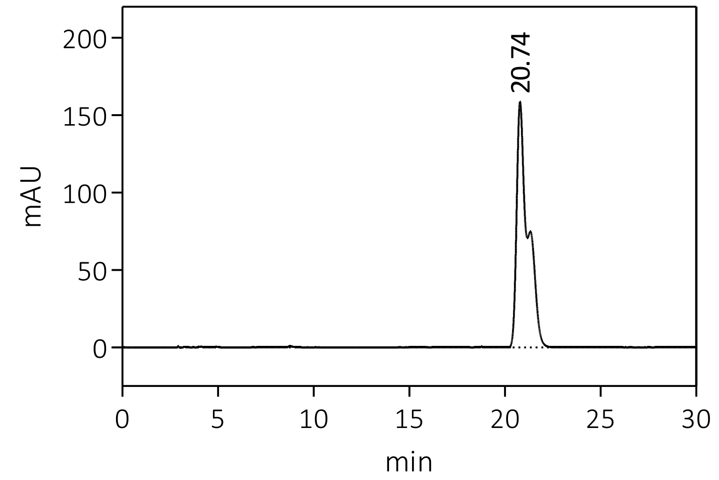

Figure S5. I) ^{1}H NMR spectrum of compound **5** (400 MHz – $CDCl_3$)

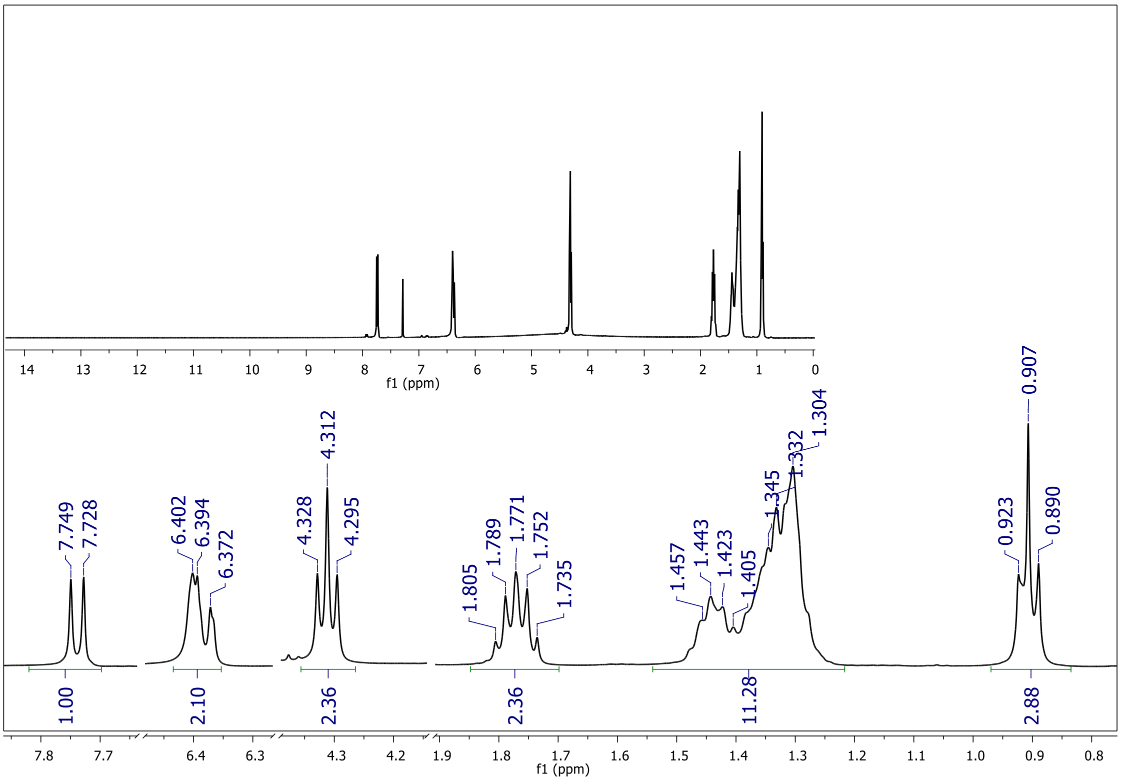

Figure S5. II) ^{13}C NMR spectrum of compound **5** (100 MHz – $CDCl_3$)

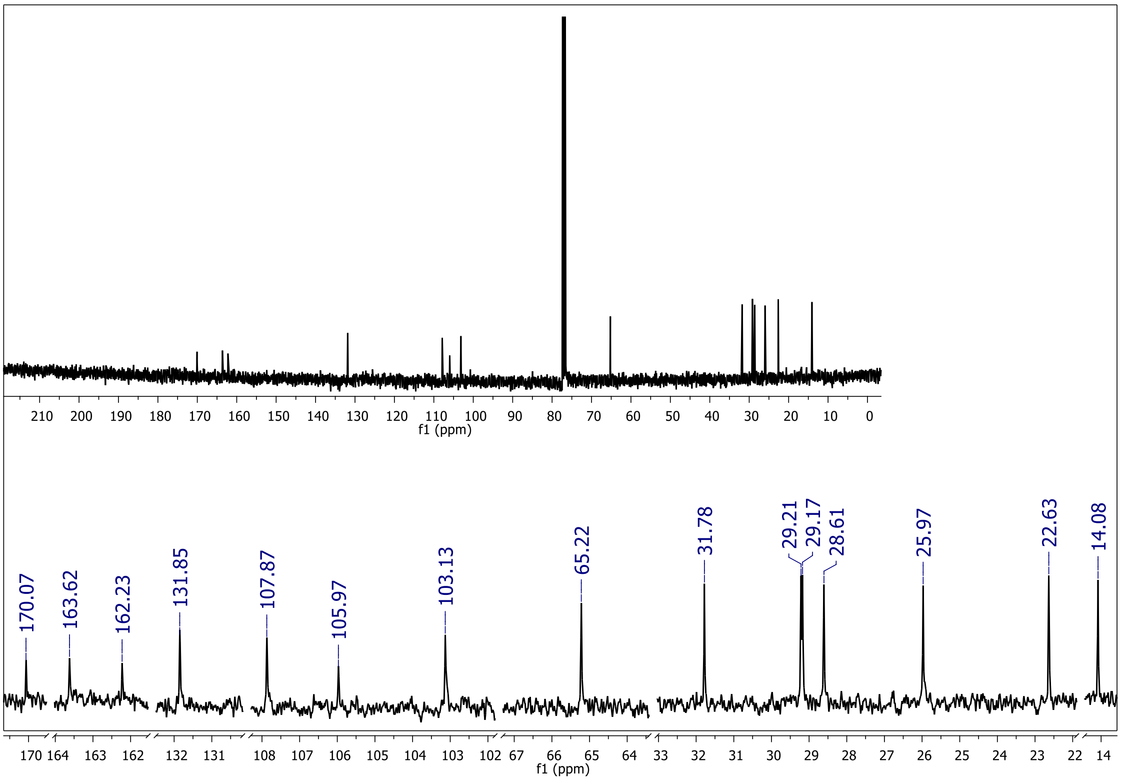

Figure S5. III) HPLC chromatogram of compound **5**

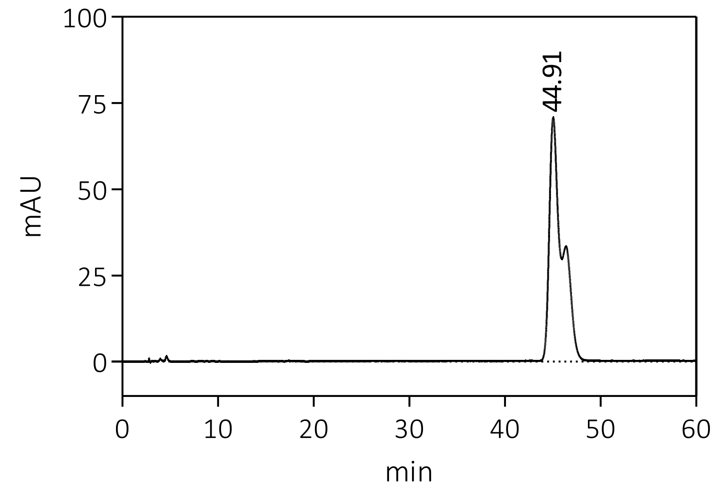

Figure S6. I) ^{1}H NMR spectrum of compound **6** (400 MHz – $CDCl_3$)

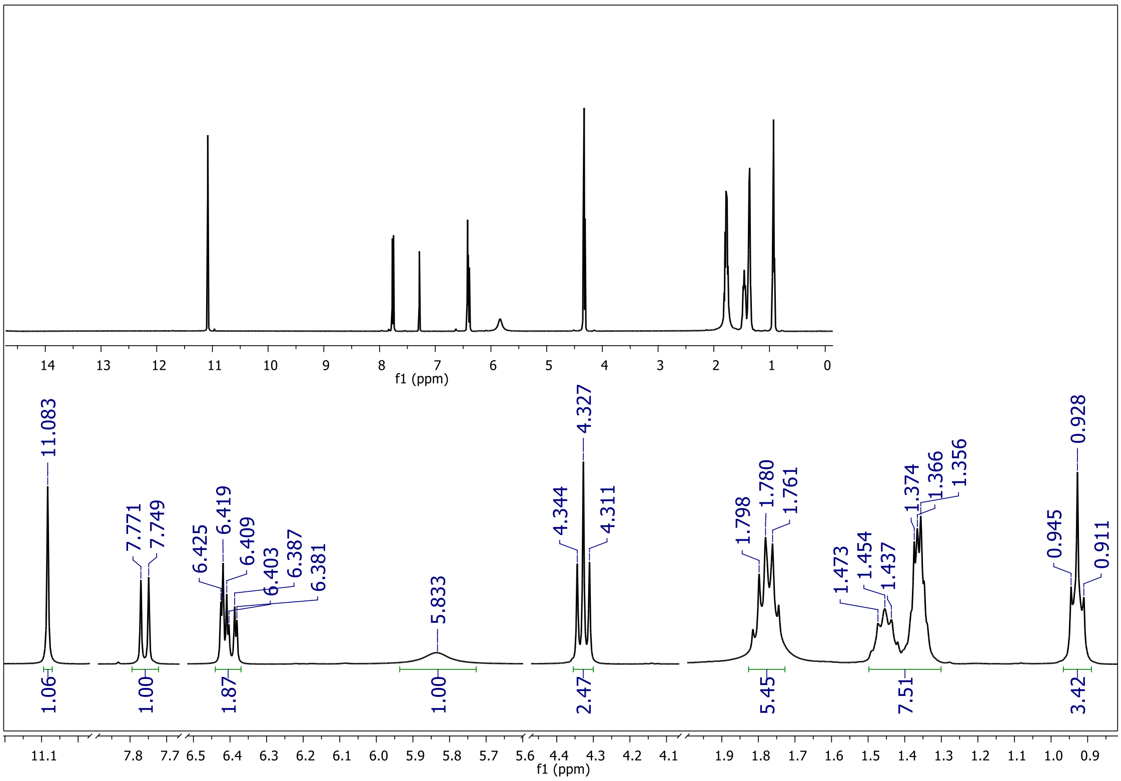

Figure S6. II) ^{13}C NMR spectrum of compound **6** (100 MHz – $CDCl_3$)

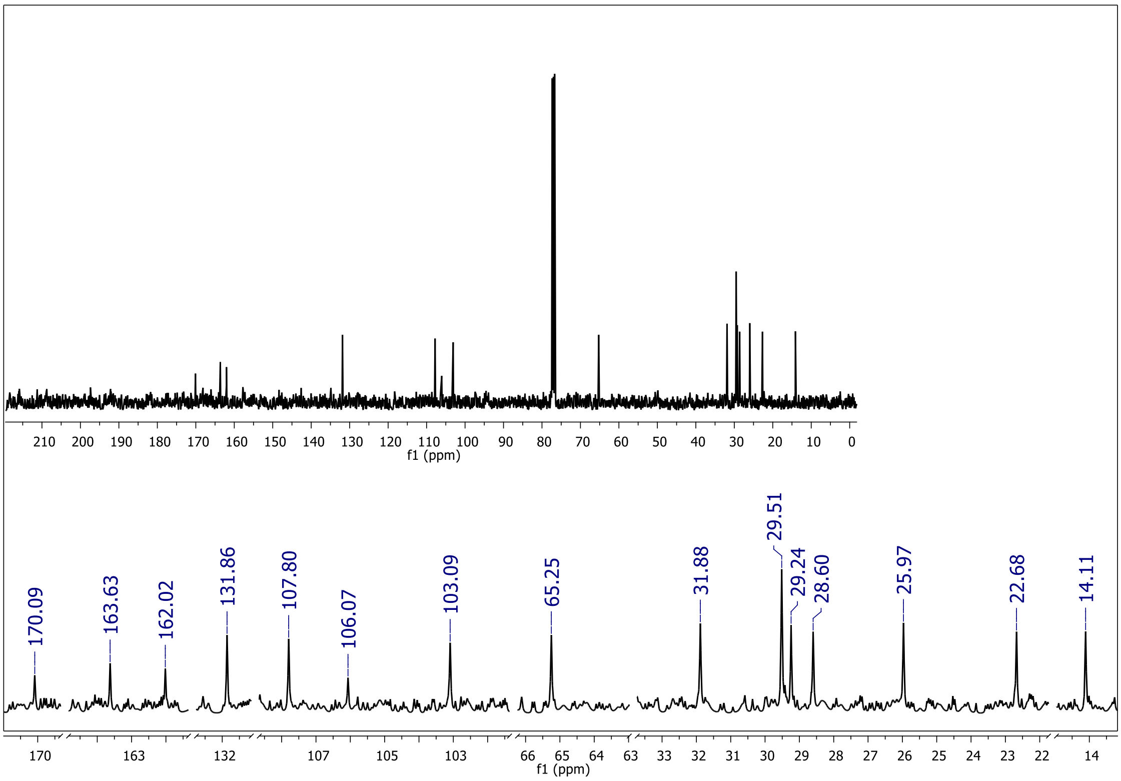

Figure S6. III) HPLC chromatogram of compound **6**

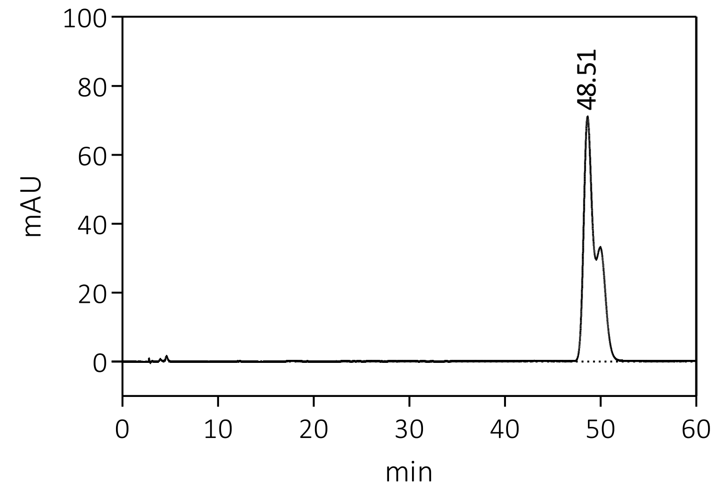

Figure S7. I) ^{1}H NMR spectrum of compound **7** (400 MHz – $CDCl_3$)

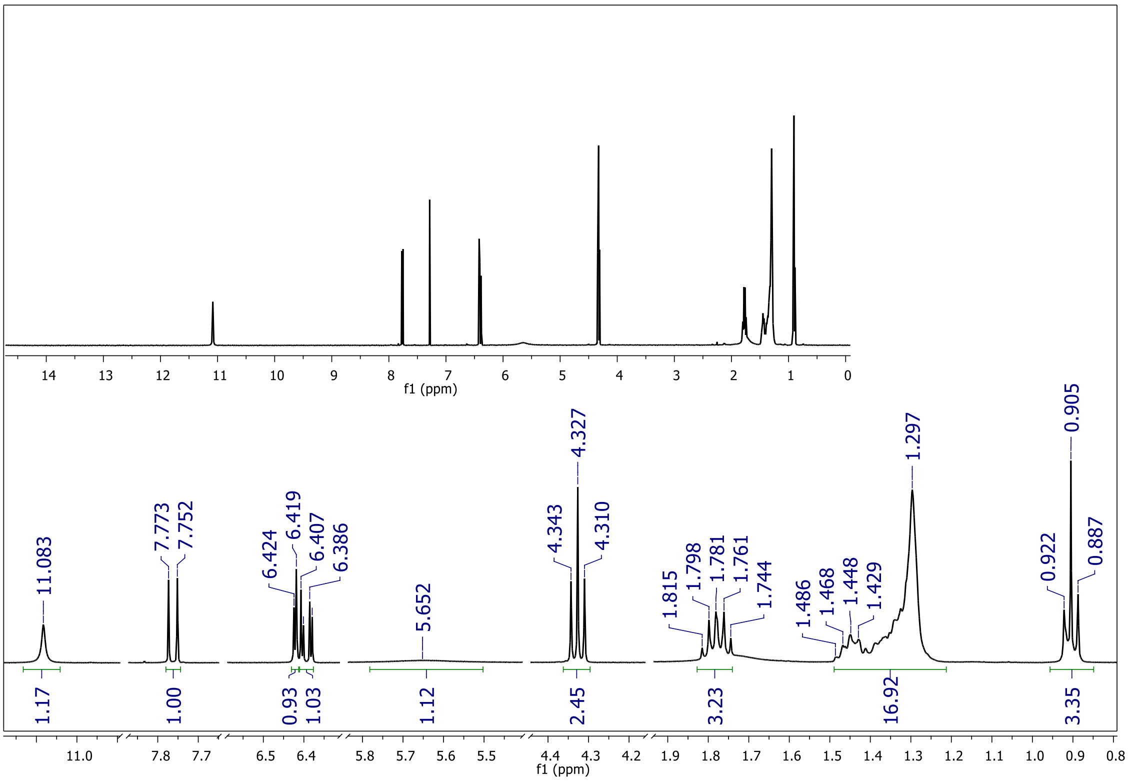

Figure S7. II) ^{13}C NMR spectrum of compound **7** (100 MHz – $CDCl_3$)

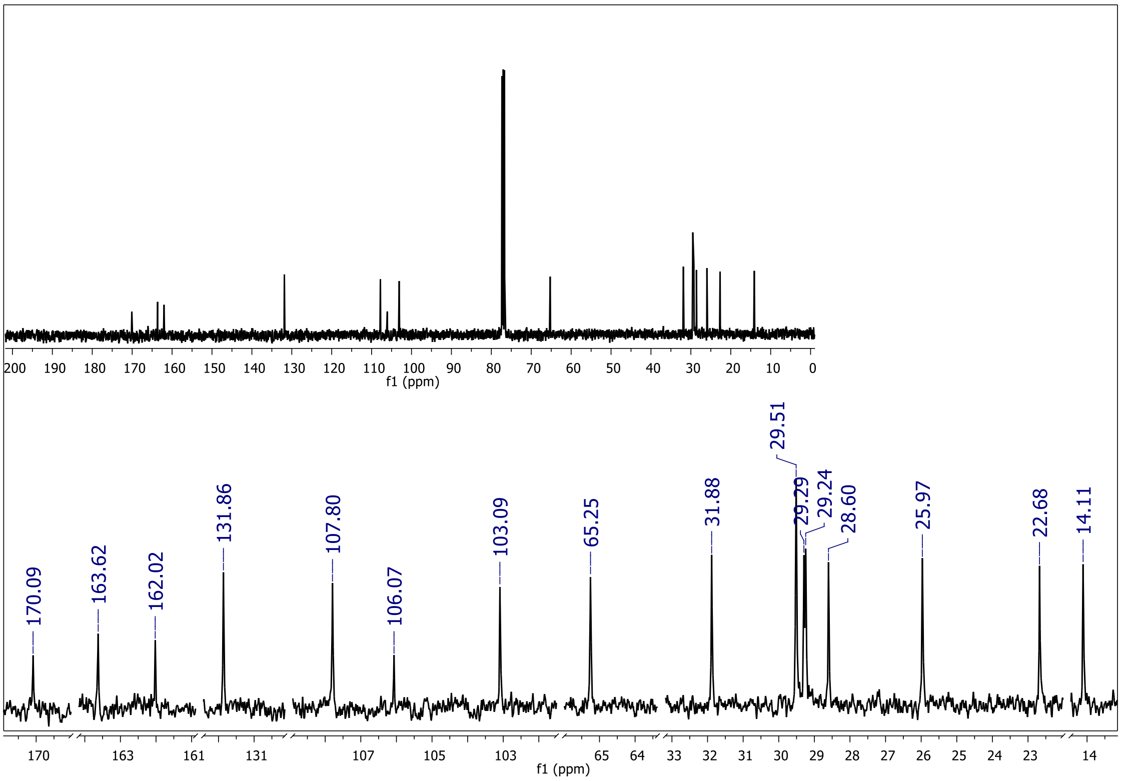

Figure S7. III) HPLC chromatogram of compound **7**

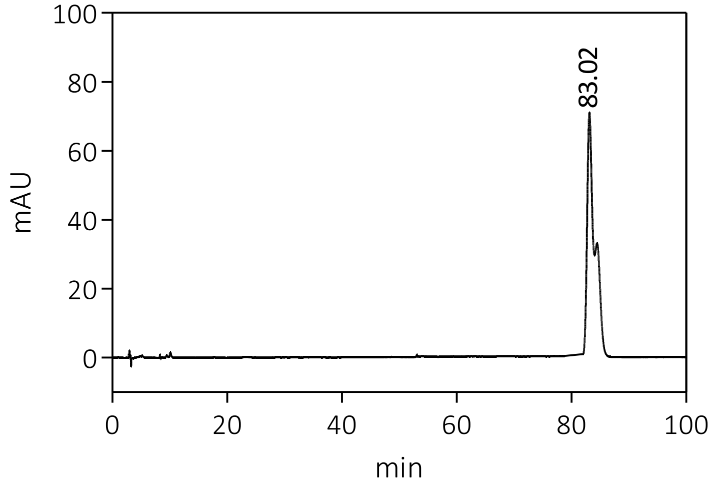

Figure S8. I) ^{1}H NMR spectrum of compound **8** (300 MHz – $CDCl_3$)

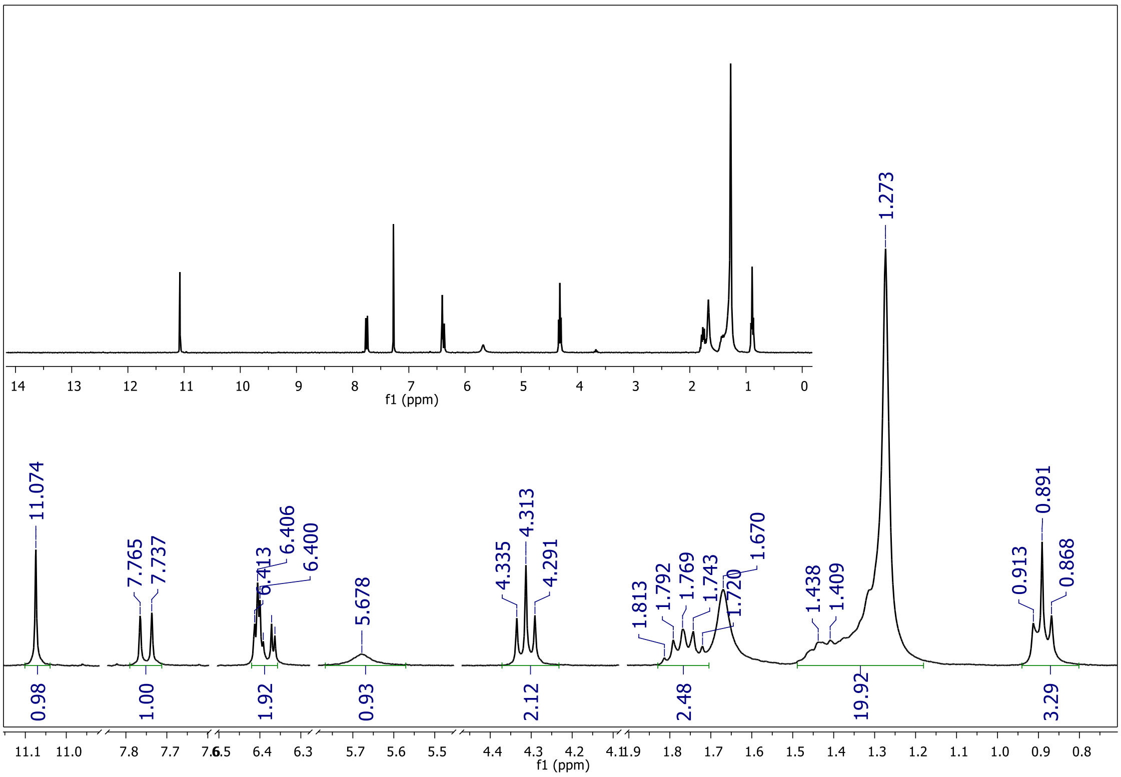

Figure S8. II) ^{13}C NMR spectrum of compound **8** (100 MHz – $CDCl_3$)

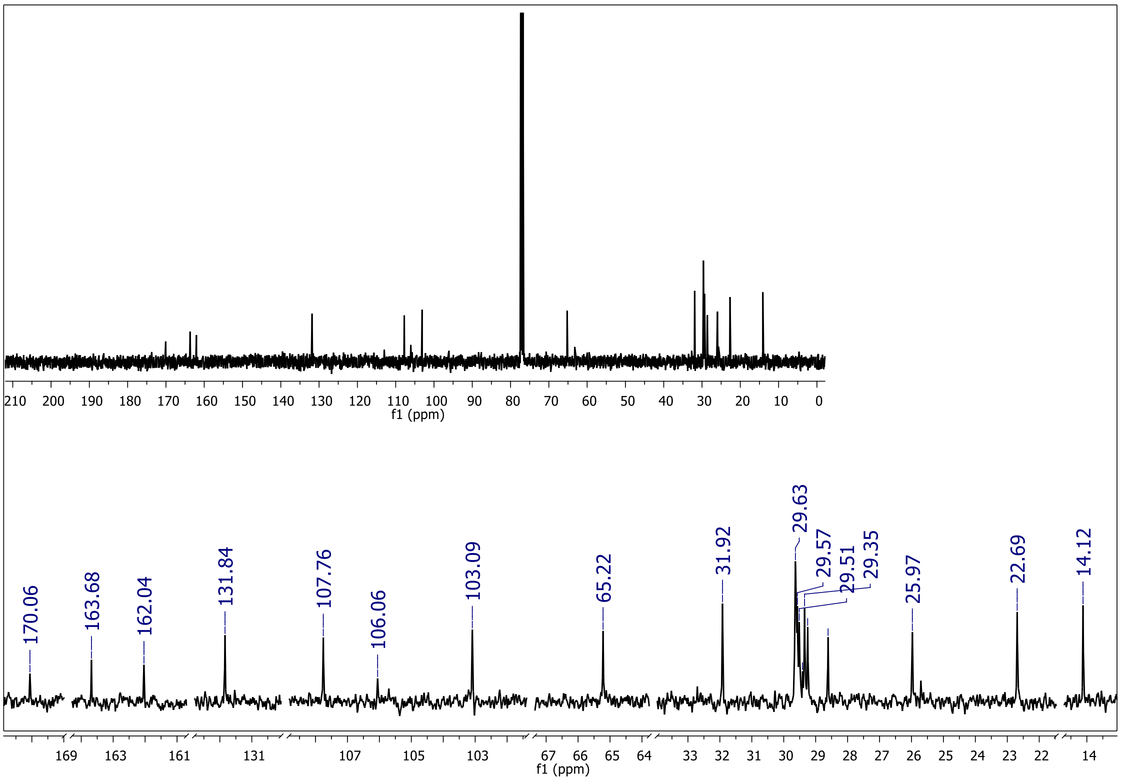

Figure S8. III) HPLC chromatogram of compound **8**

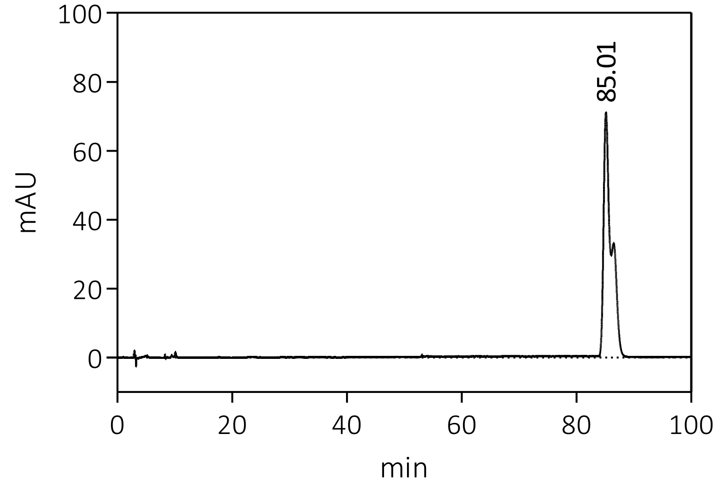

Figure S9. I) ^{1}H NMR spectrum of compound **9** (600 MHz – $CDCl_3$)

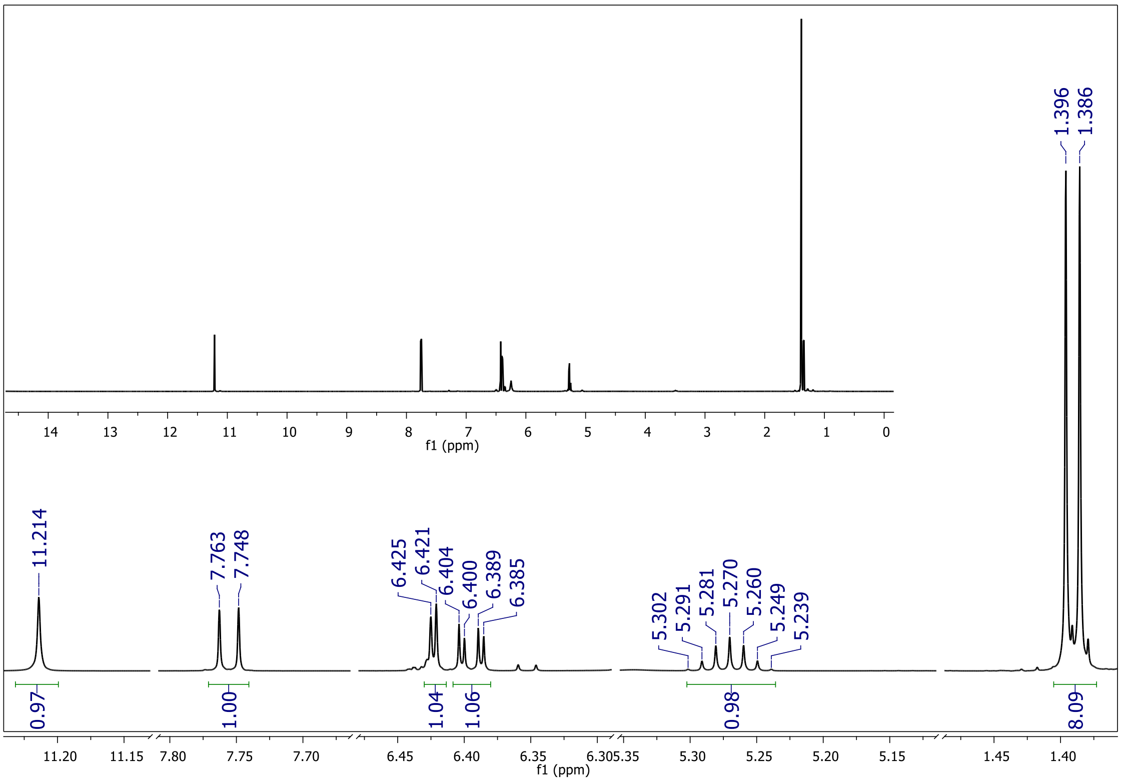

Figure S9. II) ^{13}C NMR spectrum of compound **9** (150 MHz – $CDCl_3$)

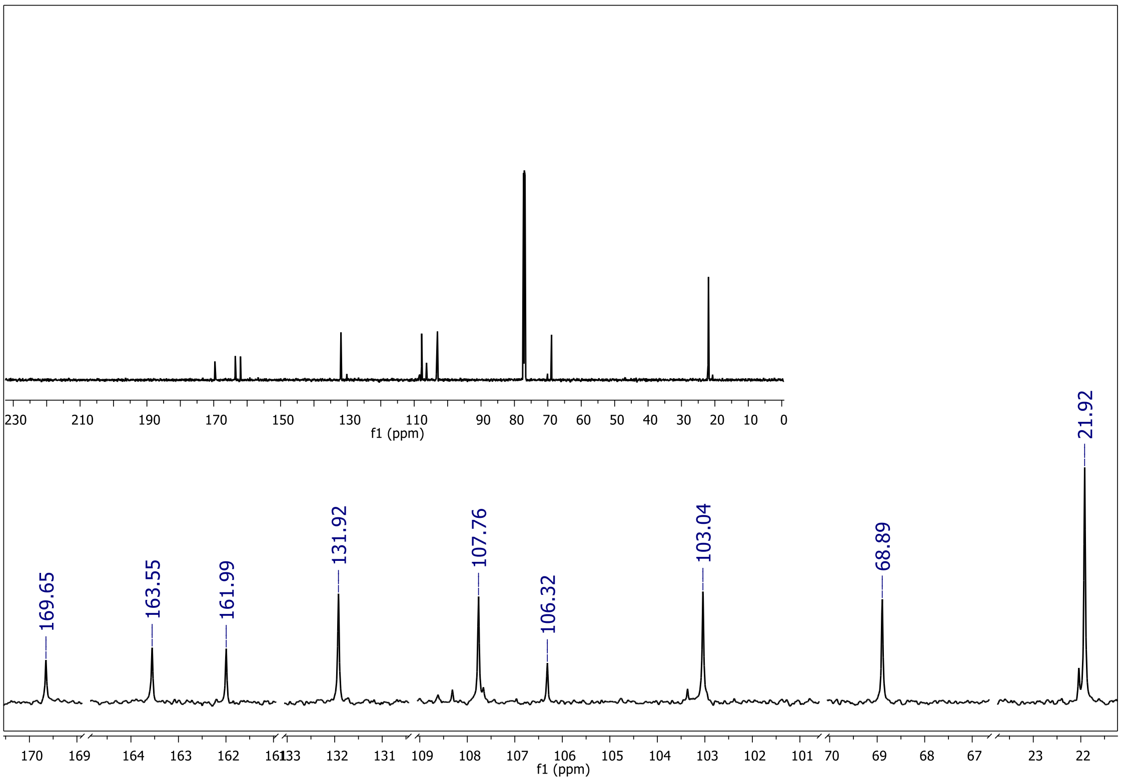

Figure S9. III) HPLC chromatogram of compound **9**

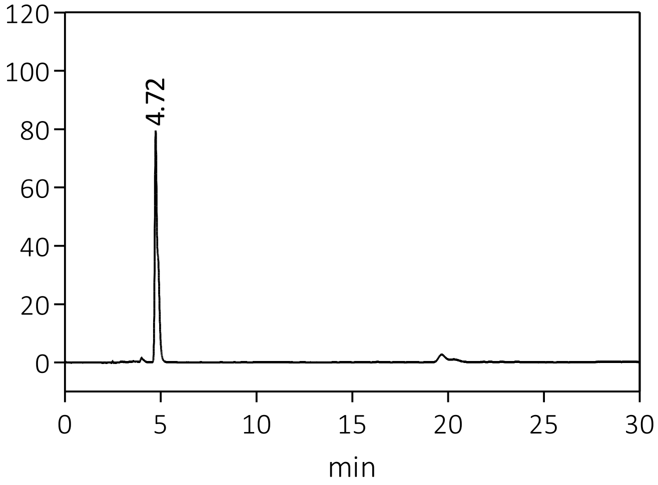

Figure S10. I) ^{1}H NMR spectrum of compound **10** (600 MHz – $CDCl_3$)

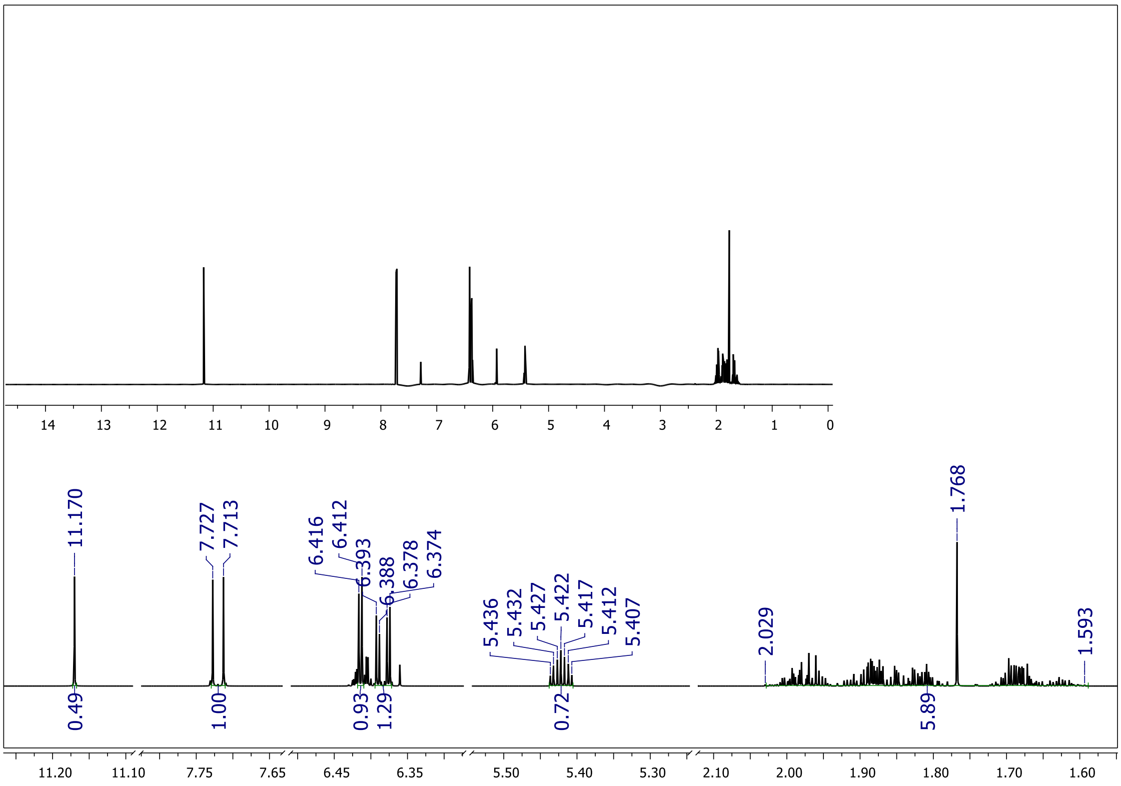

Figure S10. II) ^{13}C NMR spectrum of compound **10** (150 MHz – $CDCl_3$)

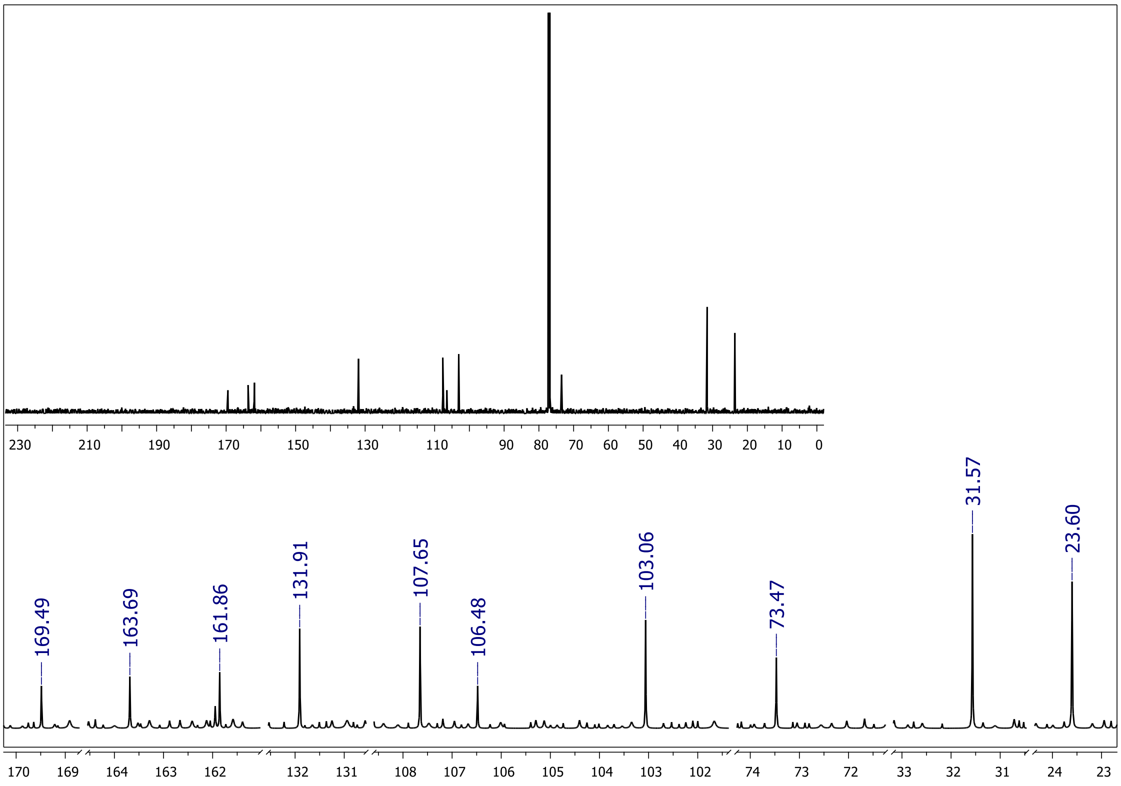

Figure S10. III) HPLC chromatogram of compound **10**

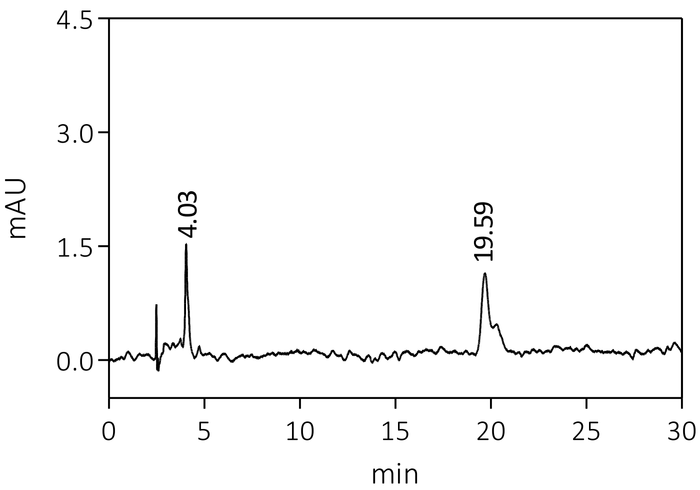

Figure S11. I) ^{1}H NMR spectrum of compound **11** (600 MHz – $CDCl_3$)

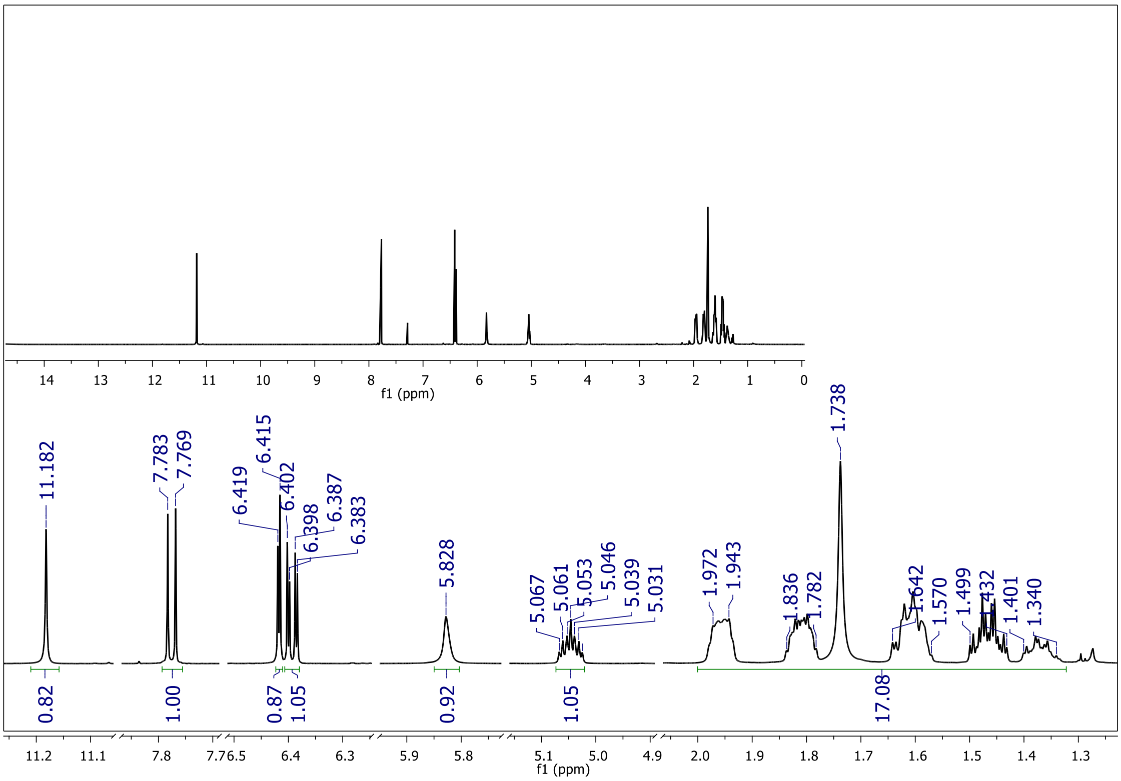

Figure S11. II) ^{13}C NMR spectrum of compound **11** (150 MHz – $CDCl_3$)

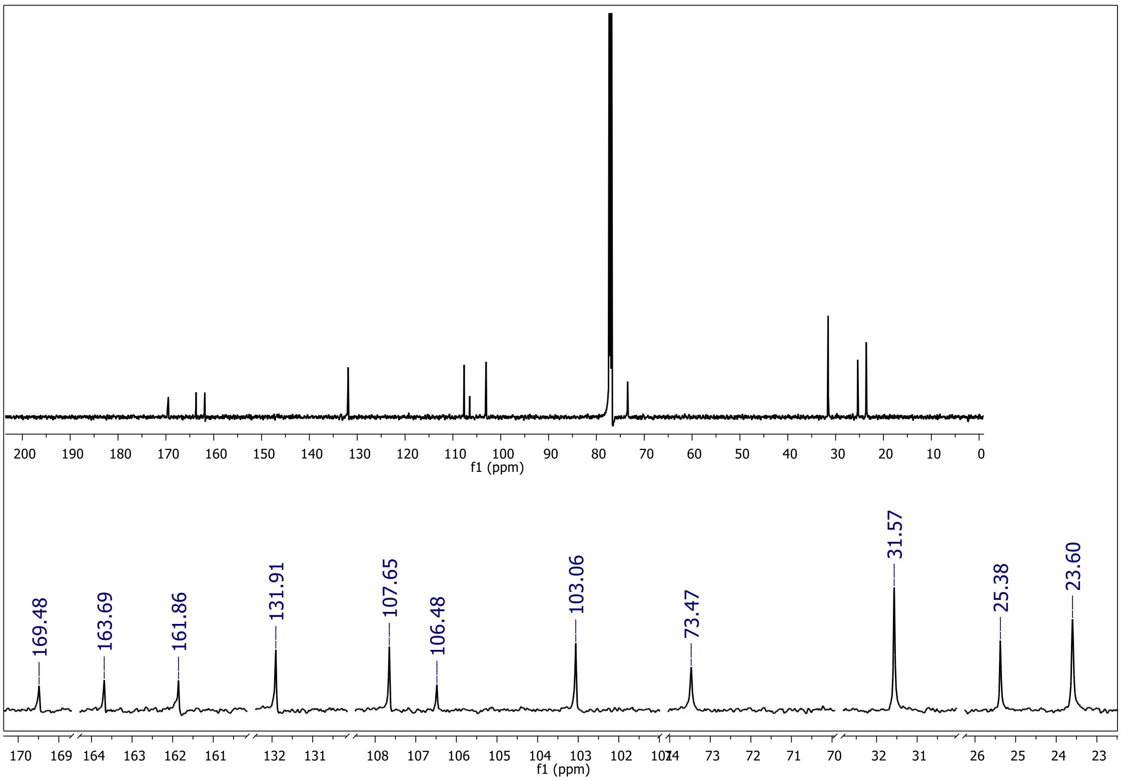

Figure S11. III) HPLC chromatogram of compound **11**

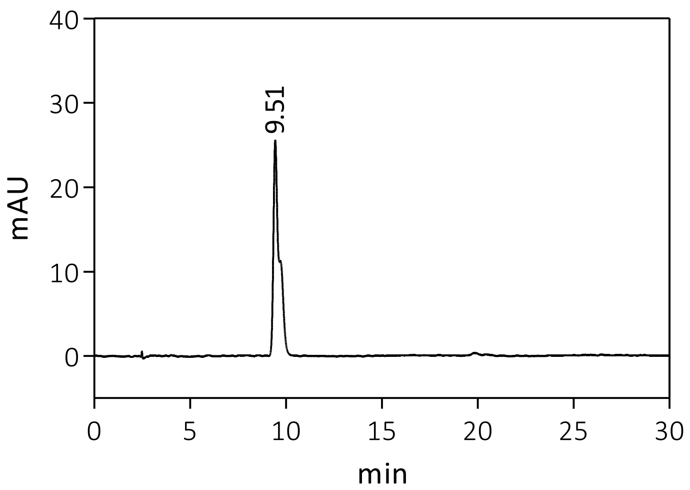

Figure S13. I) ^{1}H NMR spectrum of compound **13** (400 MHz – $CDCl_3$)

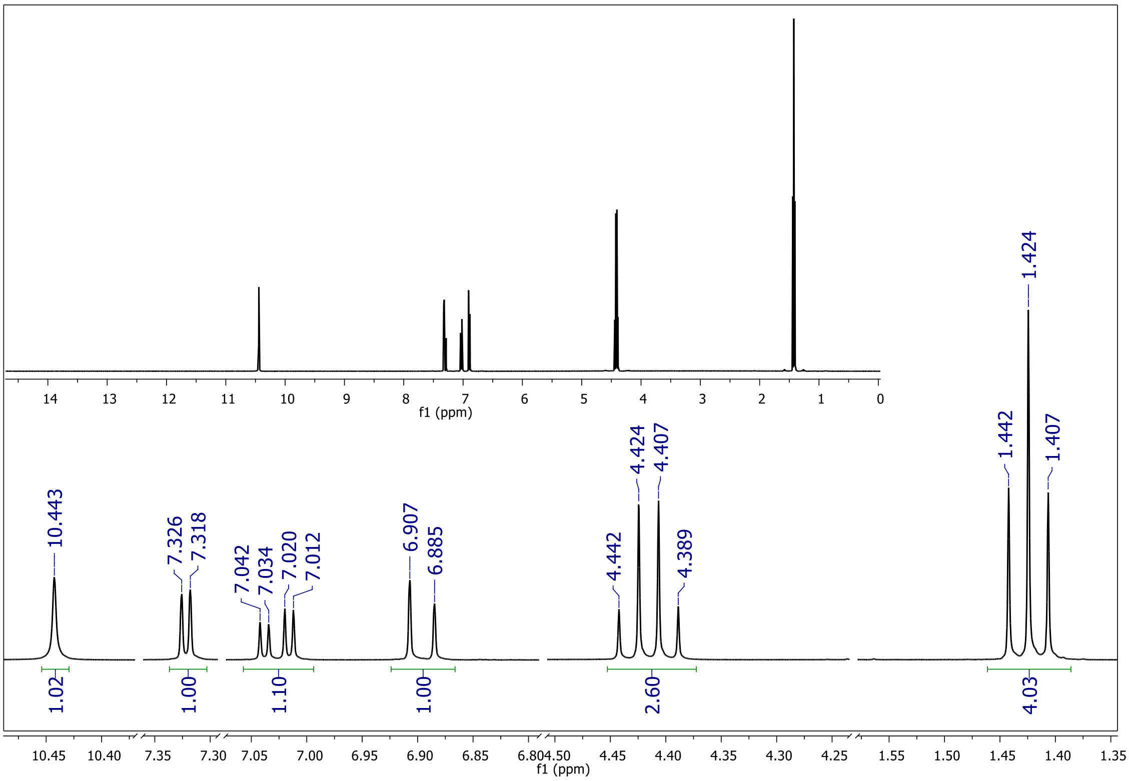

Figure S13. II) ^{13}C NMR spectrum of compound **13** (100 MHz – $CDCl_3$)

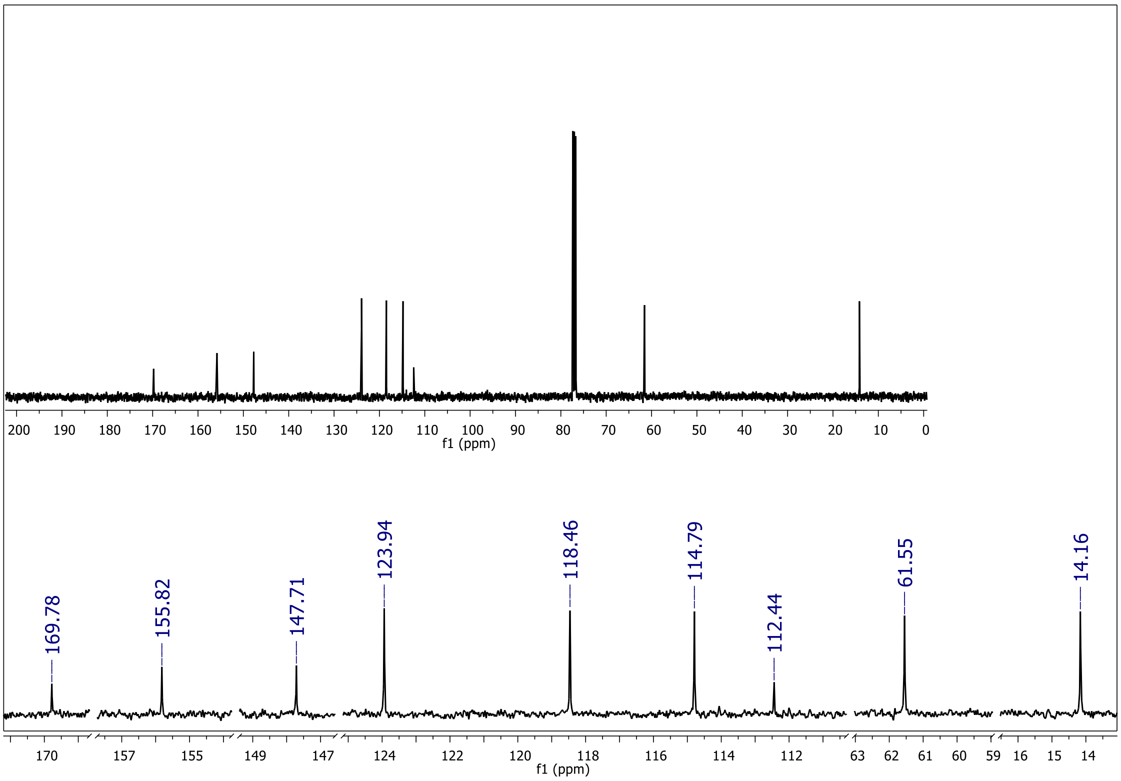

Figure S13. III) HPLC chromatogram of compound **13**

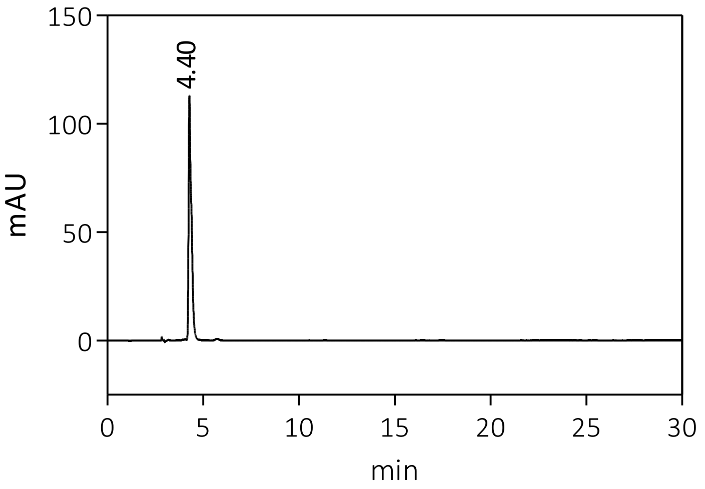

Figure S14. I) ^{1}H NMR spectrum of compound **14** (400 MHz – $CDCl_3$)

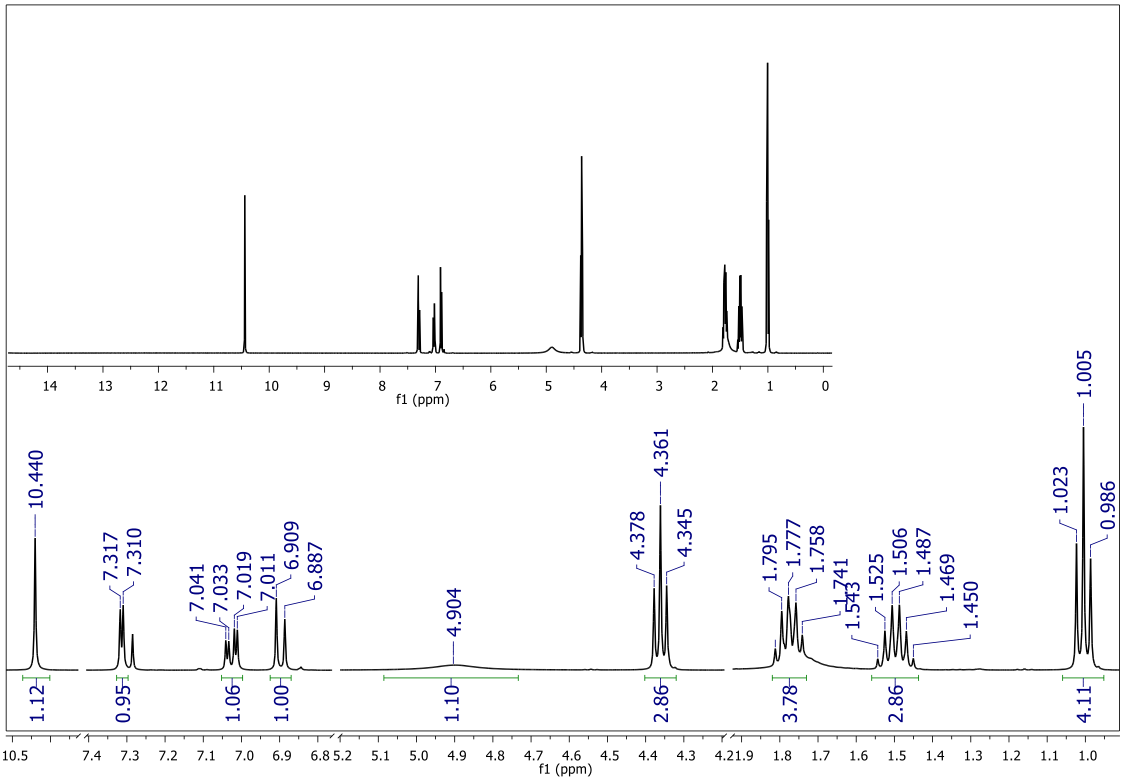

Figure S14. II) ^{13}C NMR spectrum of compound **14** (100 MHz – $CDCl_3$)

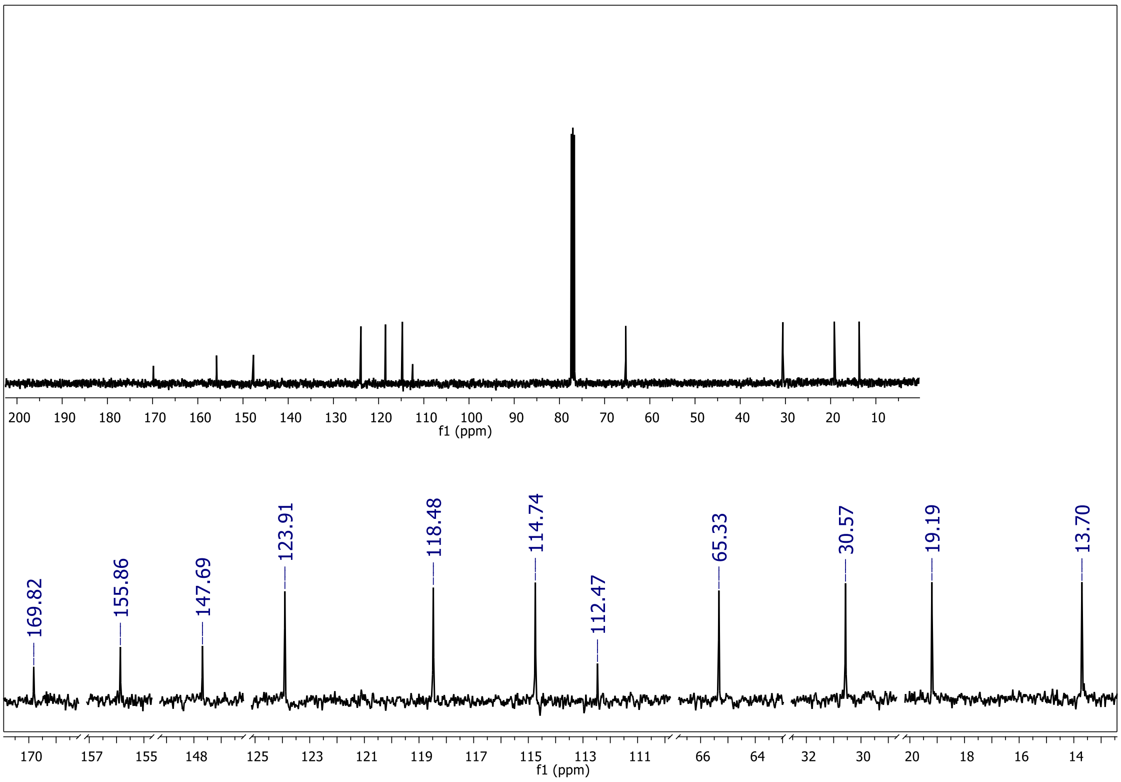

Figure S14. IIII) HPLC chromatogram of compound **14**

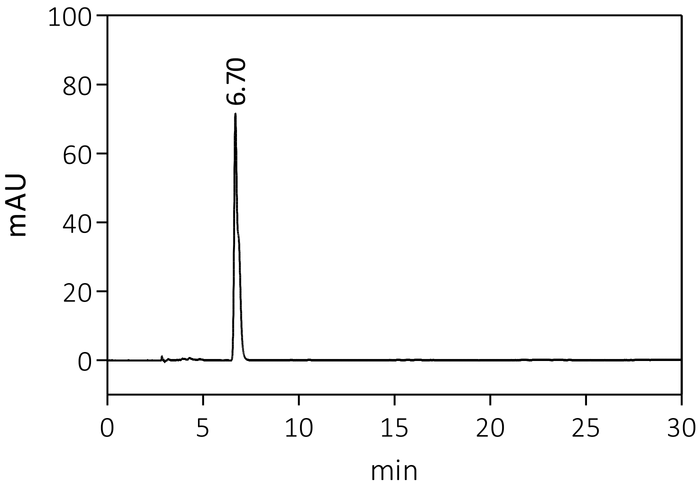

Figure S15. I) ^{1}H NMR spectrum of compound **15** (400 MHz – $CDCl_3$)

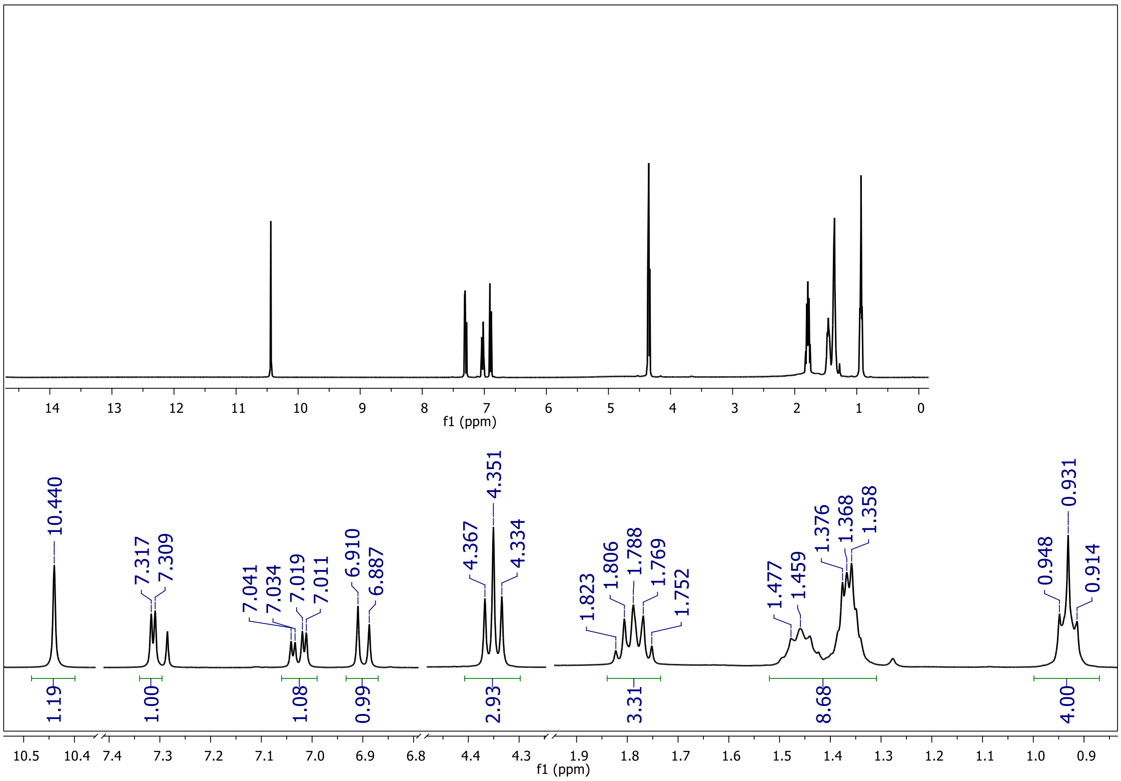

Figure S15. II) ^{13}C NMR spectrum of compound **15** (100 MHz – $CDCl_3$)

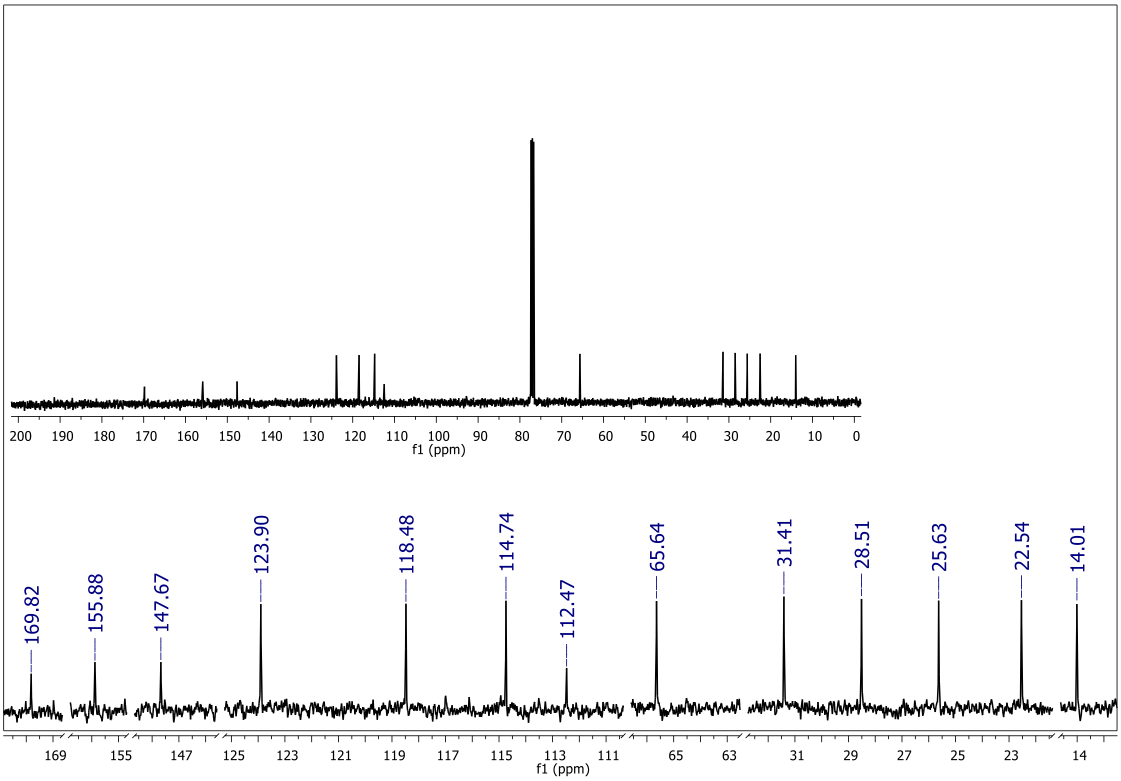

Figure S15. III) HPLC chromatogram of compound **15**

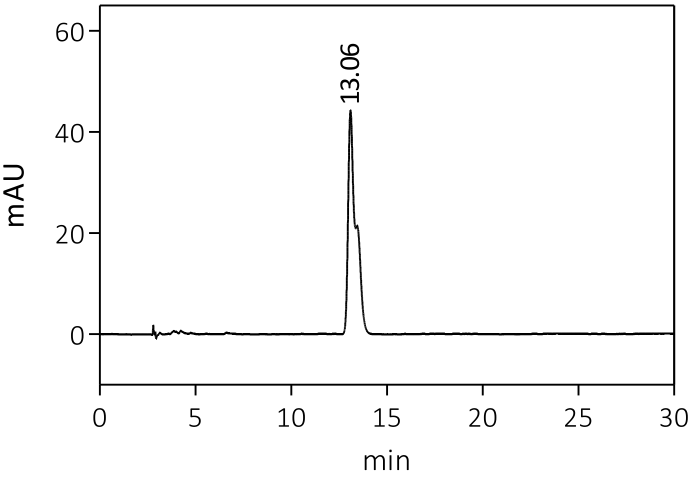

Figure S16. I) ^{1}H NMR spectrum of compound **16** (600 MHz – $CDCl_3$)

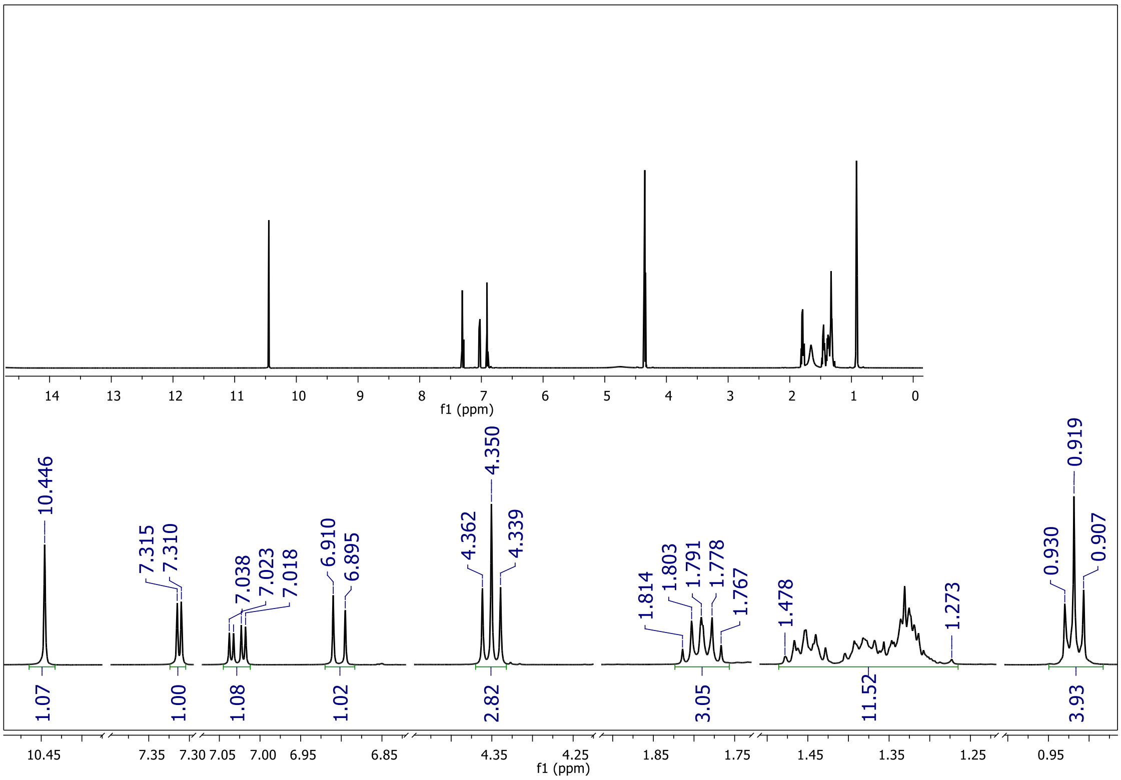

Figure S16. II) ^{13}C NMR spectrum of compound **16** (150 MHz – $CDCl_3$)

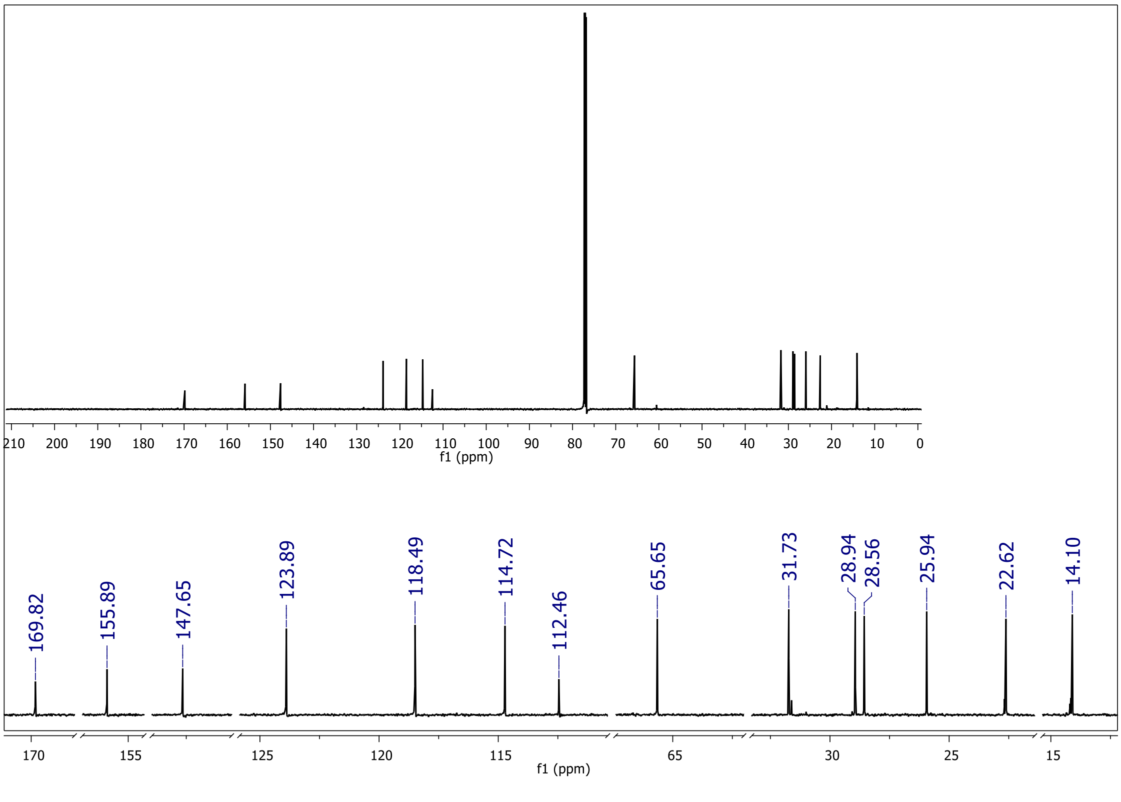

Figure S16. III) HPLC chromatogram of compound **16**

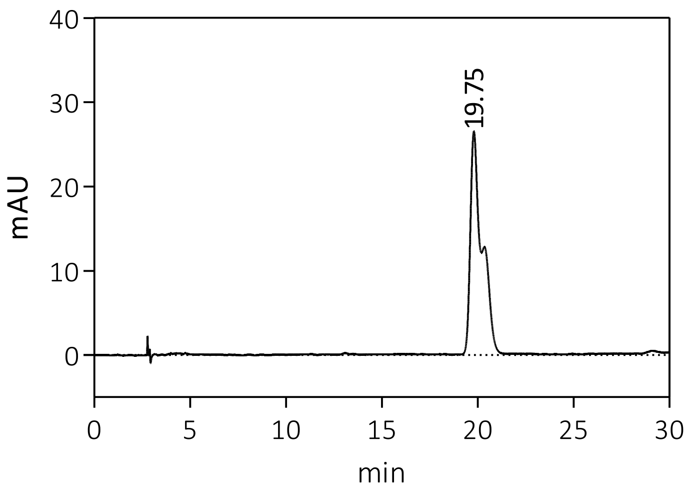

Figure S17. I) ^{1}H NMR spectrum of compound **17**(400 MHz – $CDCl_3$)

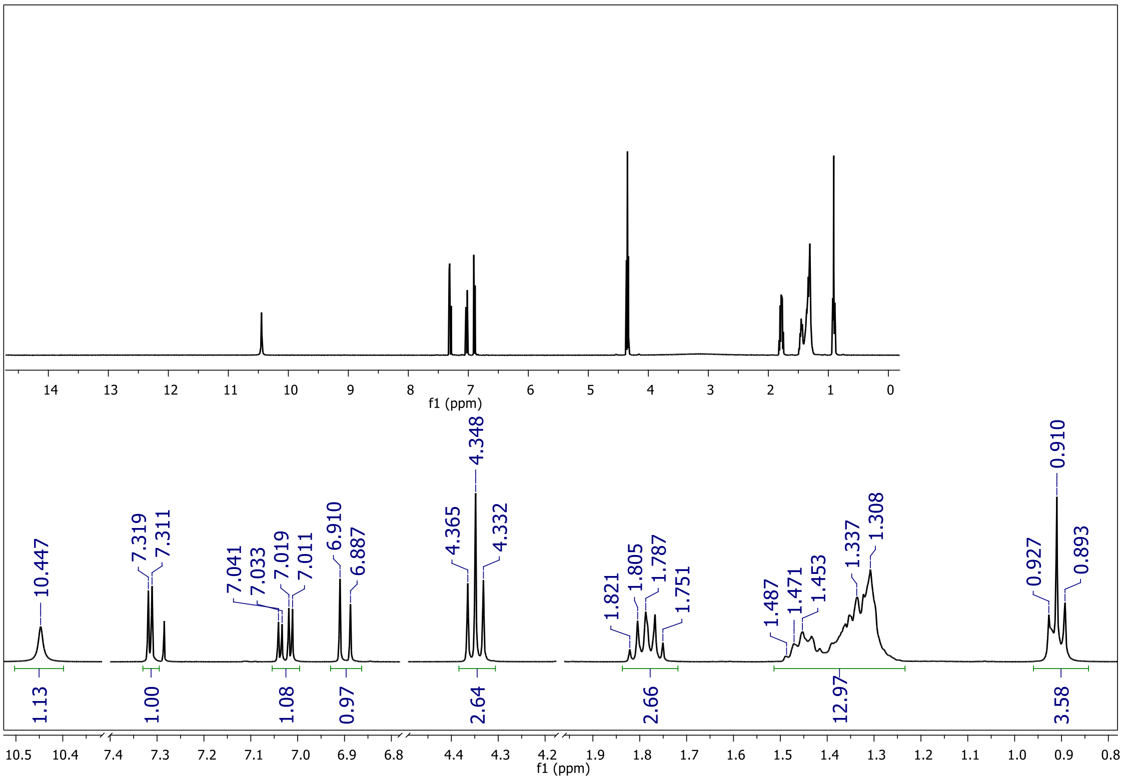

Figure S17. II) ^{13}C NMR spectrum of compound **17** (100 MHz – $CDCl_3$)

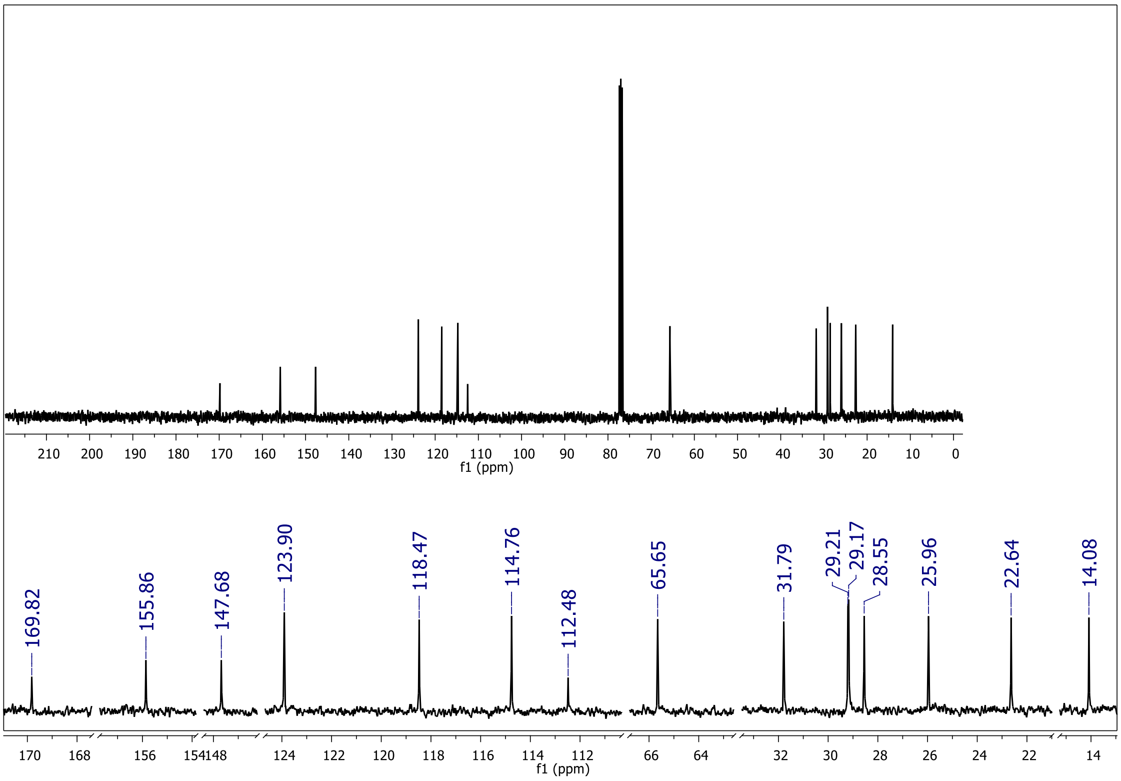

Figure S17. III) HPLC chromatogram of compound **17**

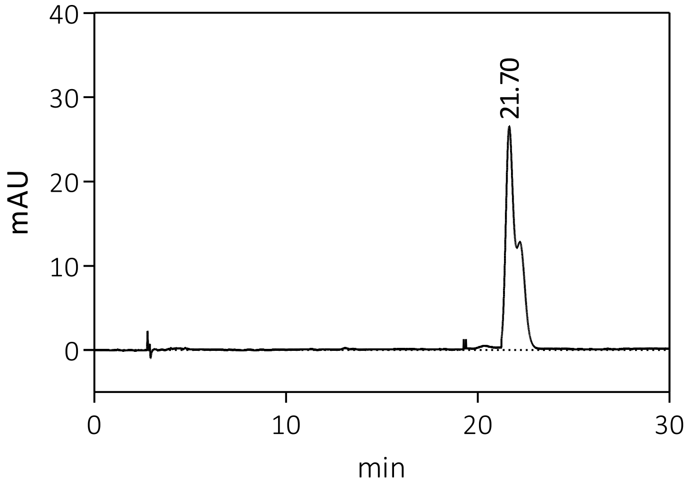

Figure S18. I) ^{1}H NMR spectrum of compound **18** (600 MHz – $CDCl_3$)

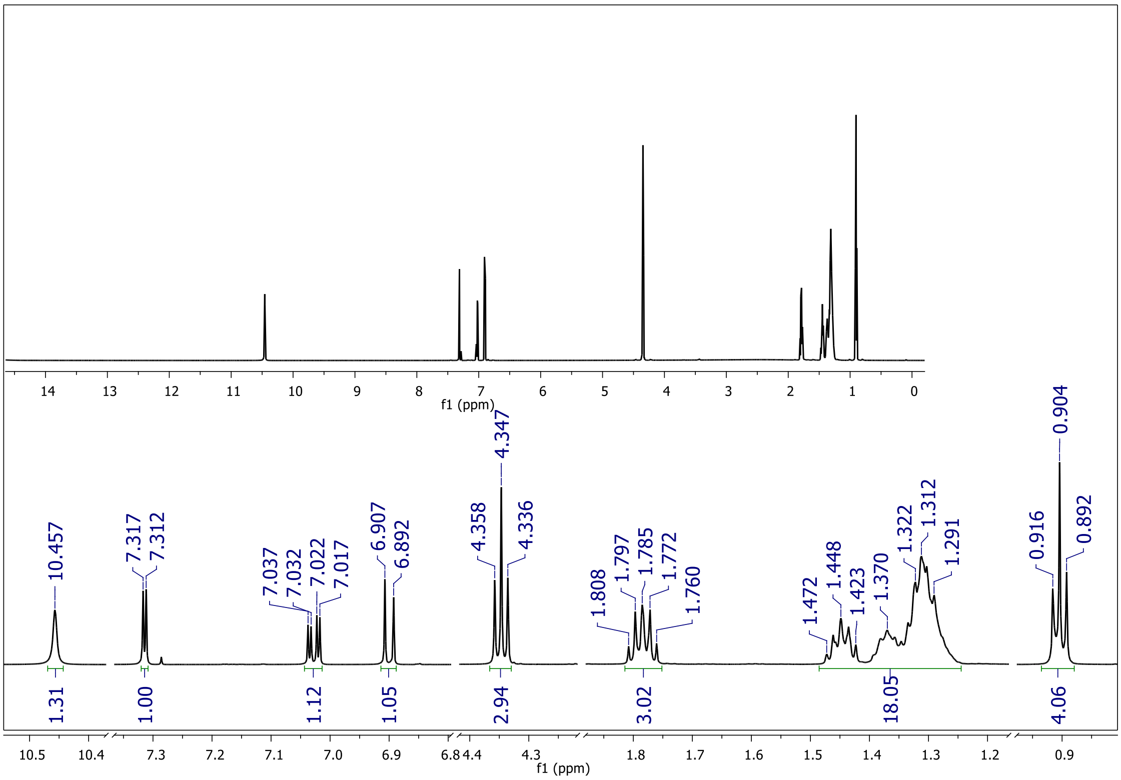

Figure S18. II) ^{13}C NMR spectrum of compound **18** (100 MHz – $CDCl_3$)

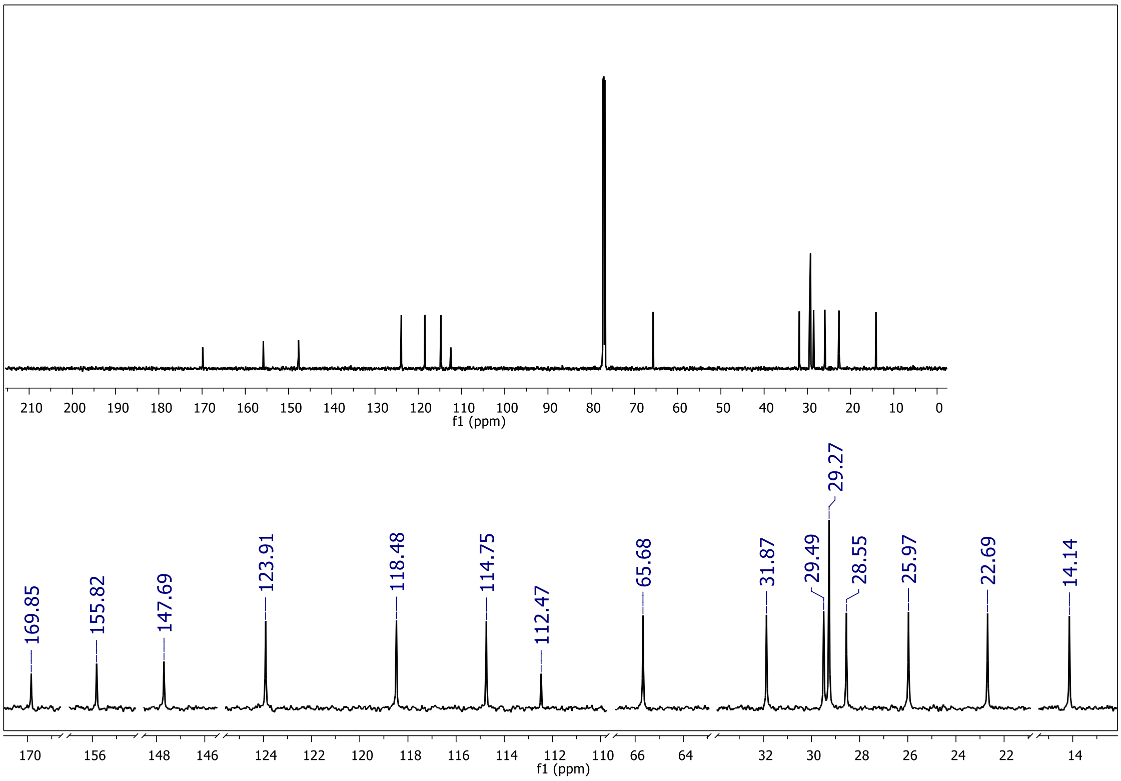

Figure S18. III) HPLC chromatogram of compound **18**

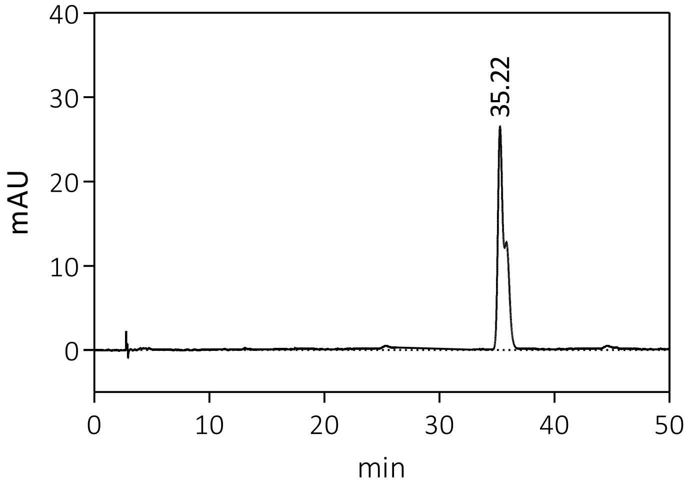

Figure S19. I) ^{1}H NMR spectrum of compound **19** (400 MHz – $CDCl_3$)

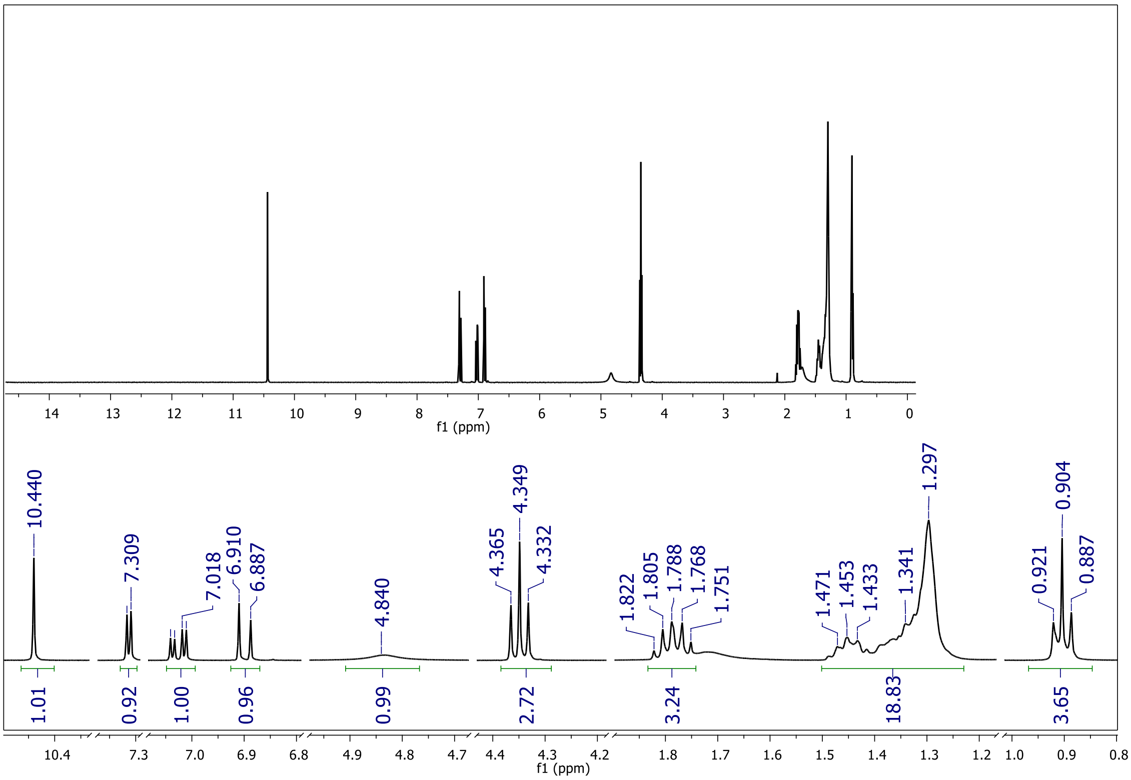

Figure S19. II) ^{13}C NMR spectrum of compound **19** (100 MHz – $CDCl_3$)

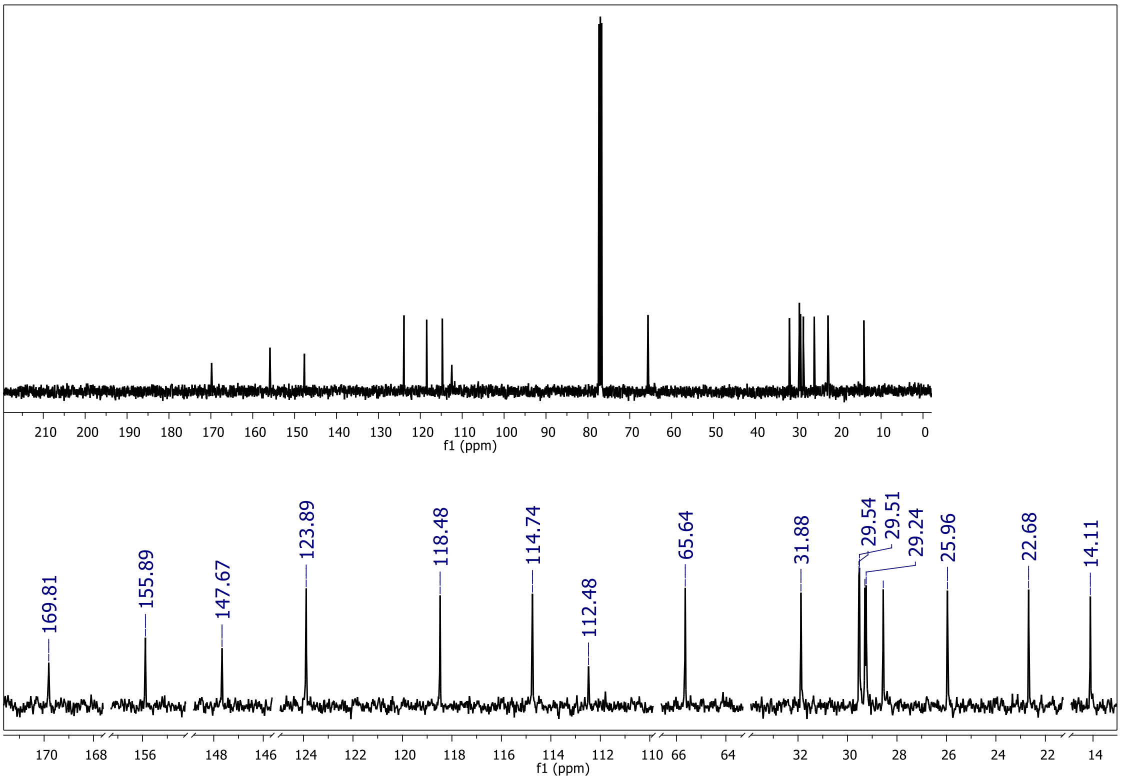

Figure S19. III) HPLC chromatogram of compound **19**

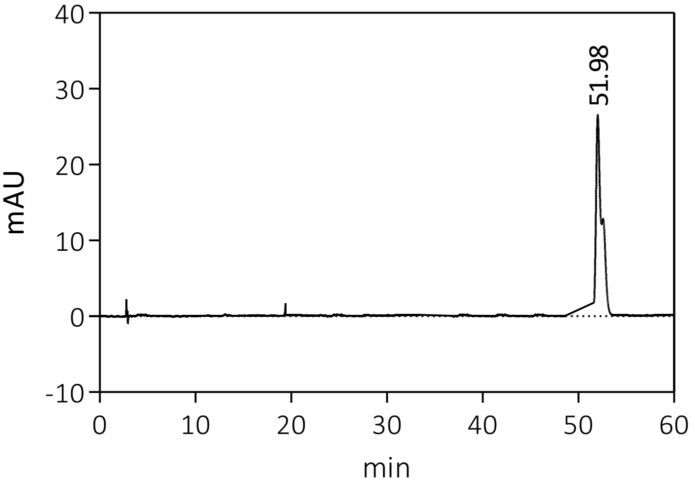

Figure S20. I) ^{1}H NMR spectrum of compound **20** (300 MHz – $CDCl_3$)

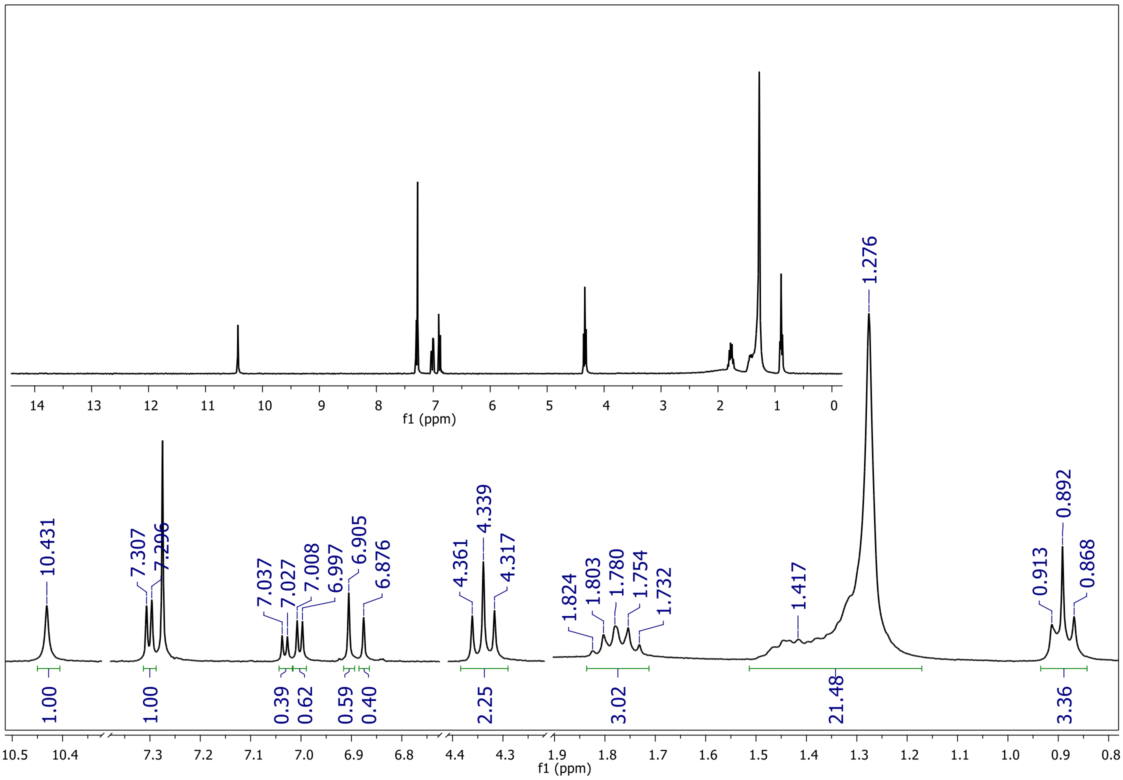

Figure S20. II) ^{13}C NMR spectrum of compound **20** (100 MHz – $CDCl_3$)

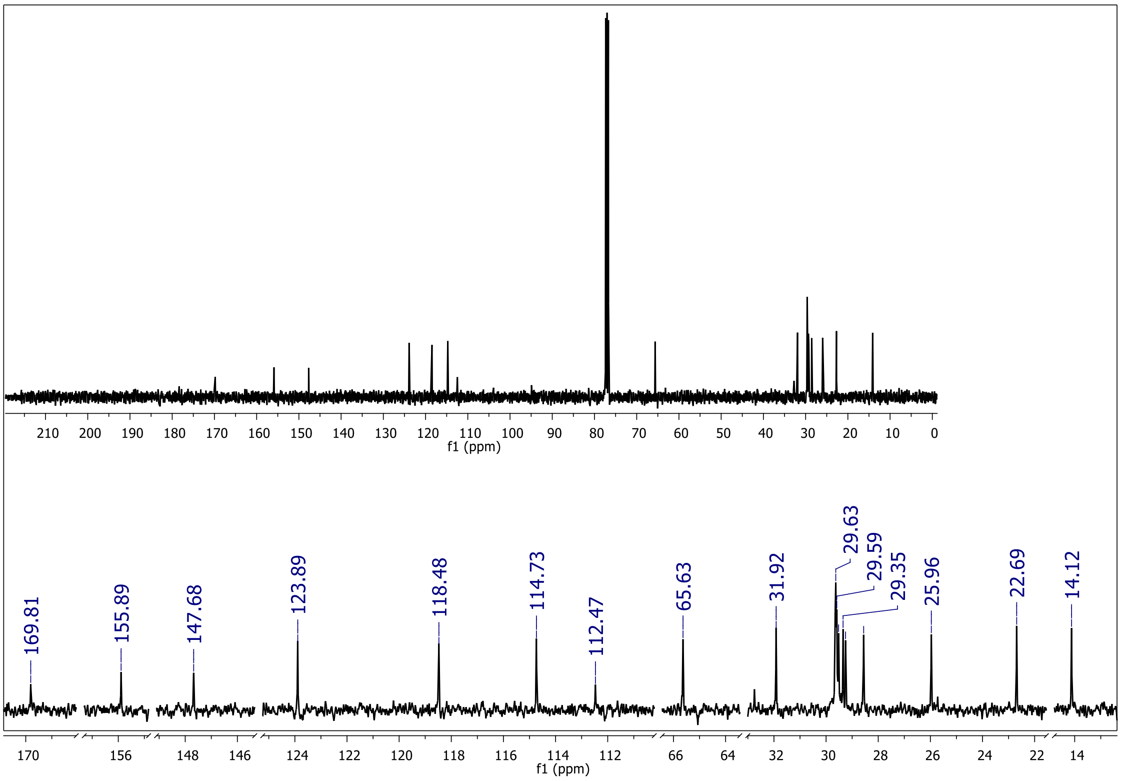

Figure S20. III) HPLC chromatogram of compound **20**

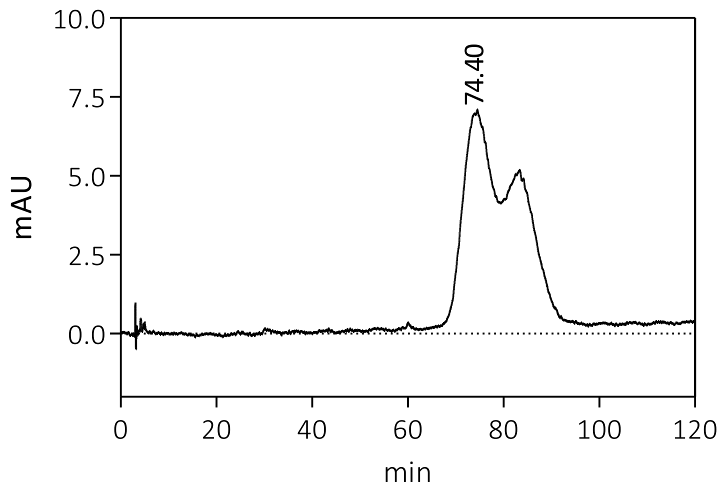

Figure S21. I) ^{1}H NMR spectrum of compound **21** (400 MHz – $CDCl_3$)

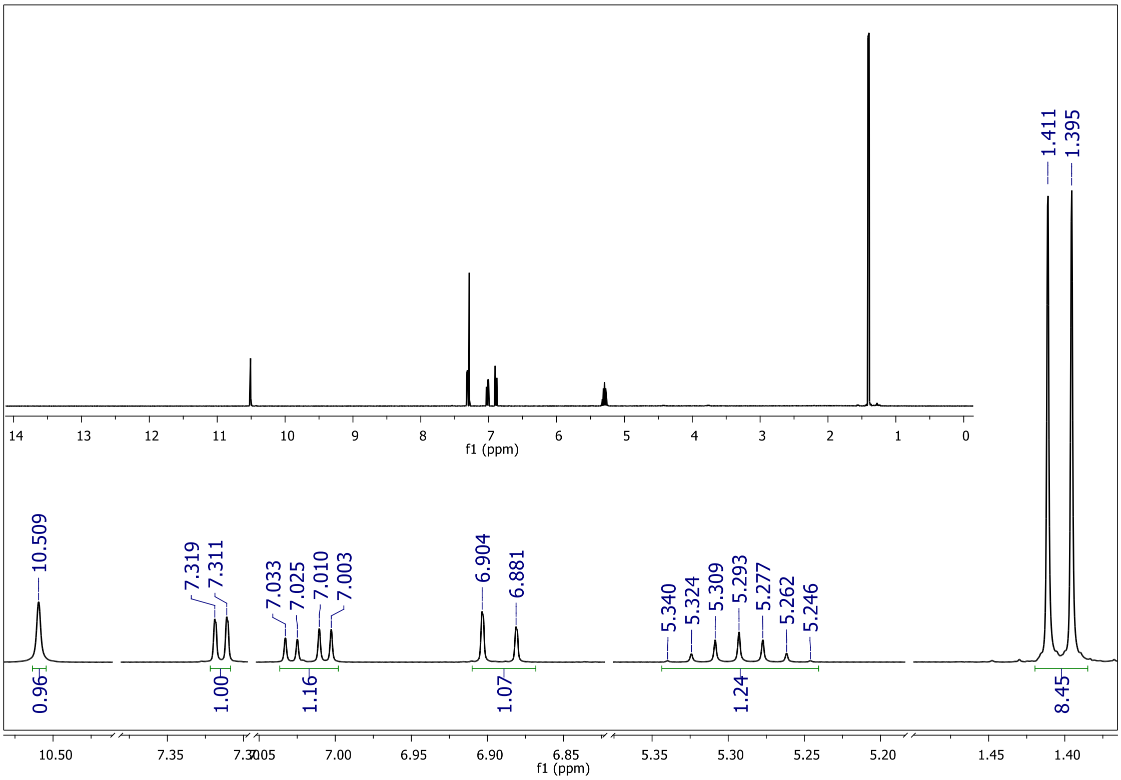

Figure S21. II) ^{13}C NMR spectrum of compound **21** (150 MHz – $CDCl_3$)

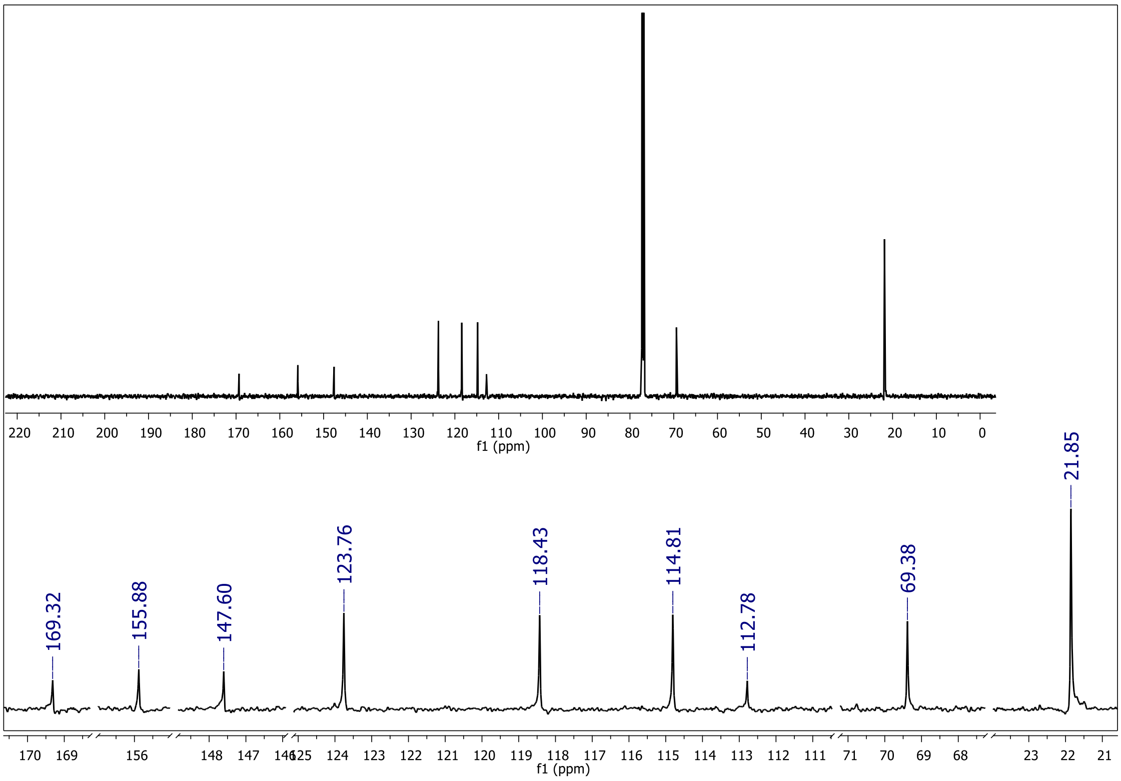

Figure S21. III) HPLC chromatogram of compound **21**

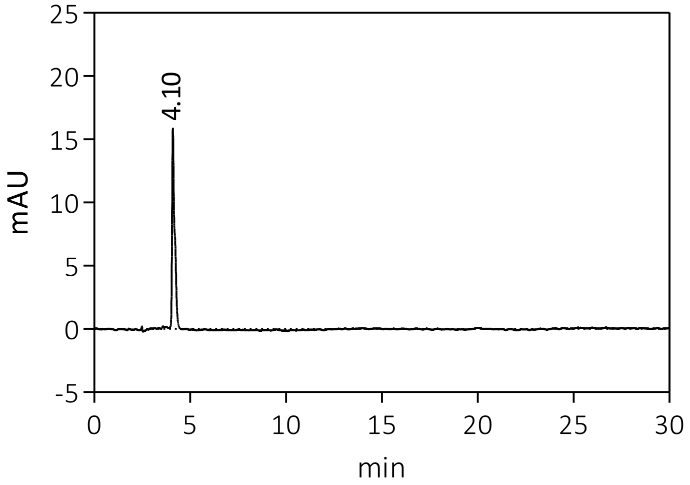

Figure S22. I) ^{1}H NMR spectrum of compound **22** (300 MHz – $CDCl_3$)

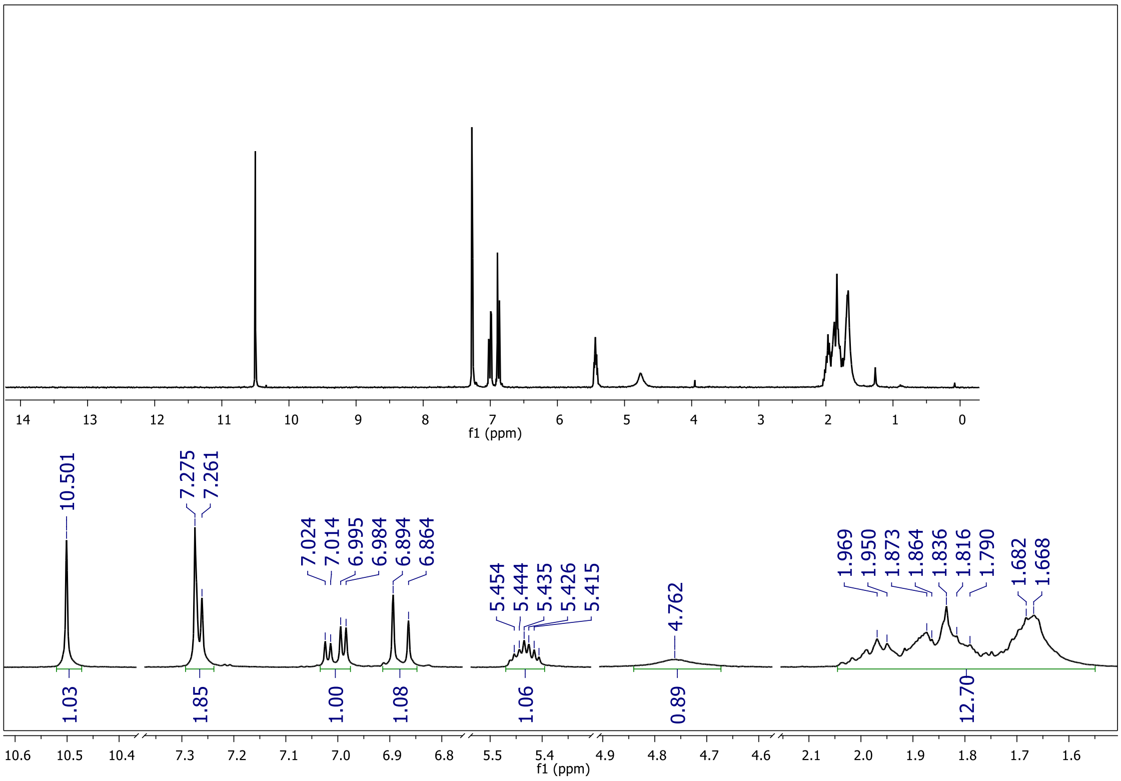

Figure S22. II) ^{13}C NMR spectrum of compound **22** (150 MHz – $CDCl_3$)

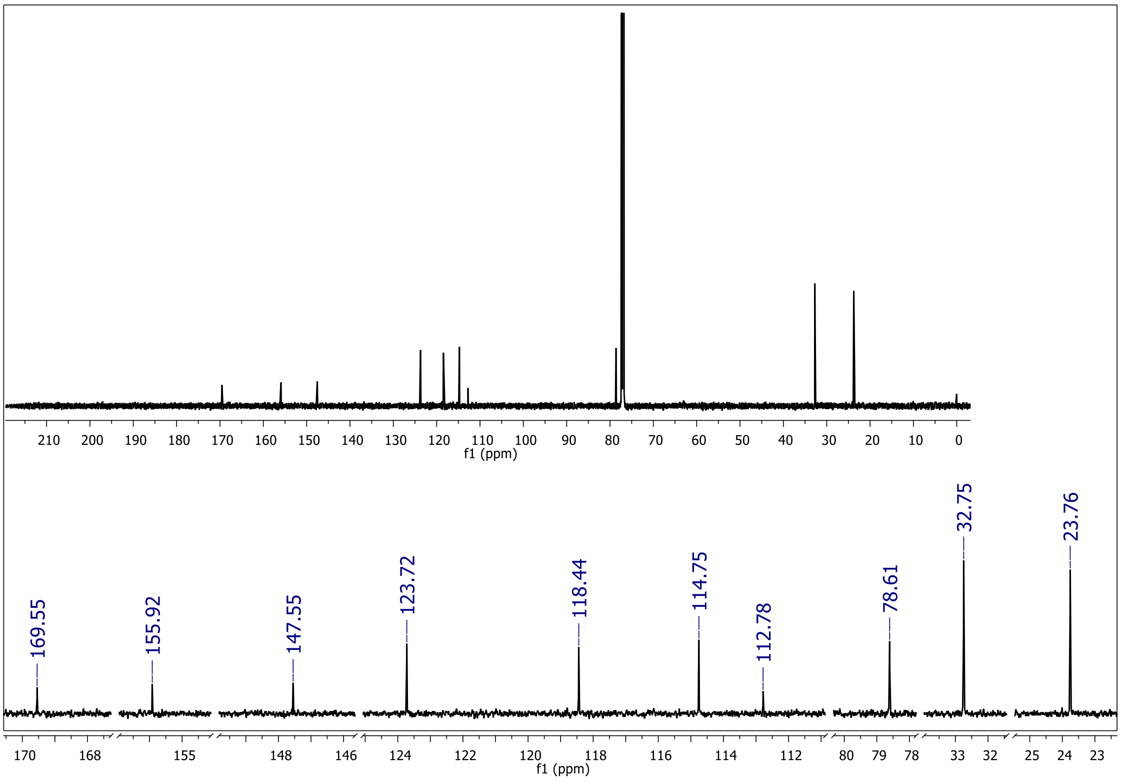

Figure S22. III) HPLC chromatogram of compound **22**

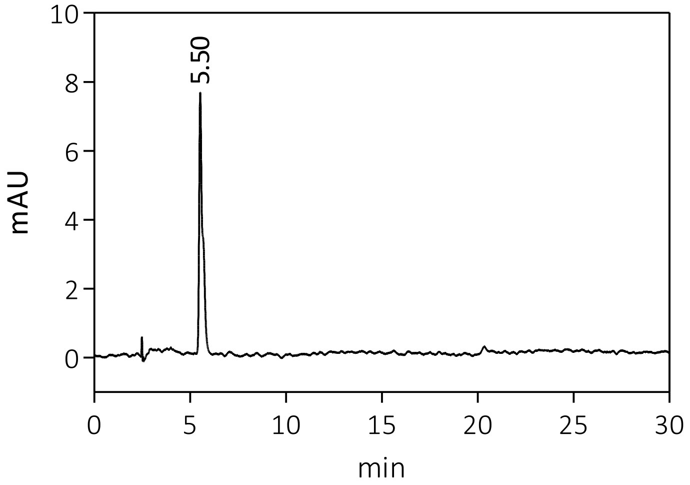

Figure S23. I) ^{1}H NMR spectrum of compound **23** (300 MHz – $CDCl_3$)

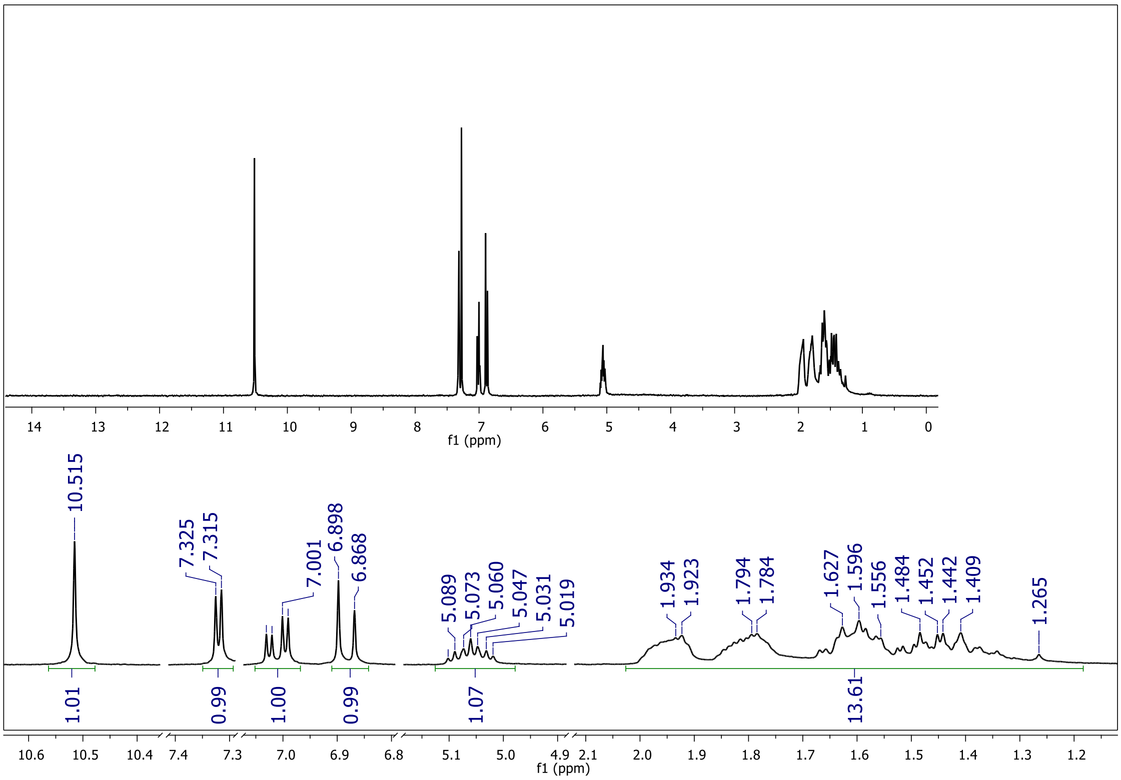

Figure S23. II) ^{13}C NMR spectrum of compound **23** (150 MHz – $CDCl_3$)

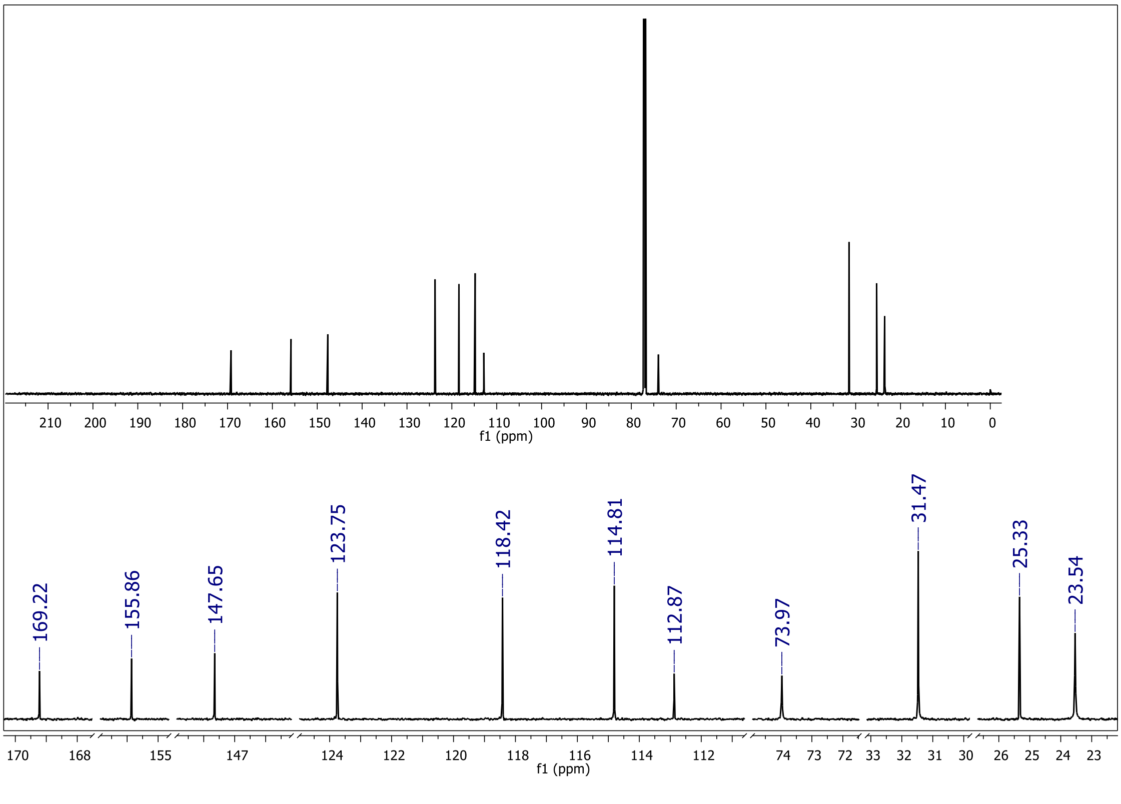

Figure S23. III) HPLC chromatogram of compound **23**

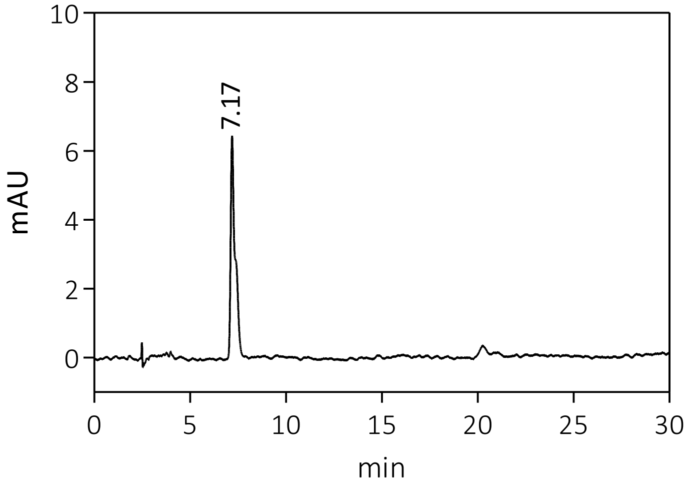

Figure S24. I) ^{1}H NMR spectrum of compound **24** (400 MHz – $CDCl_3$)

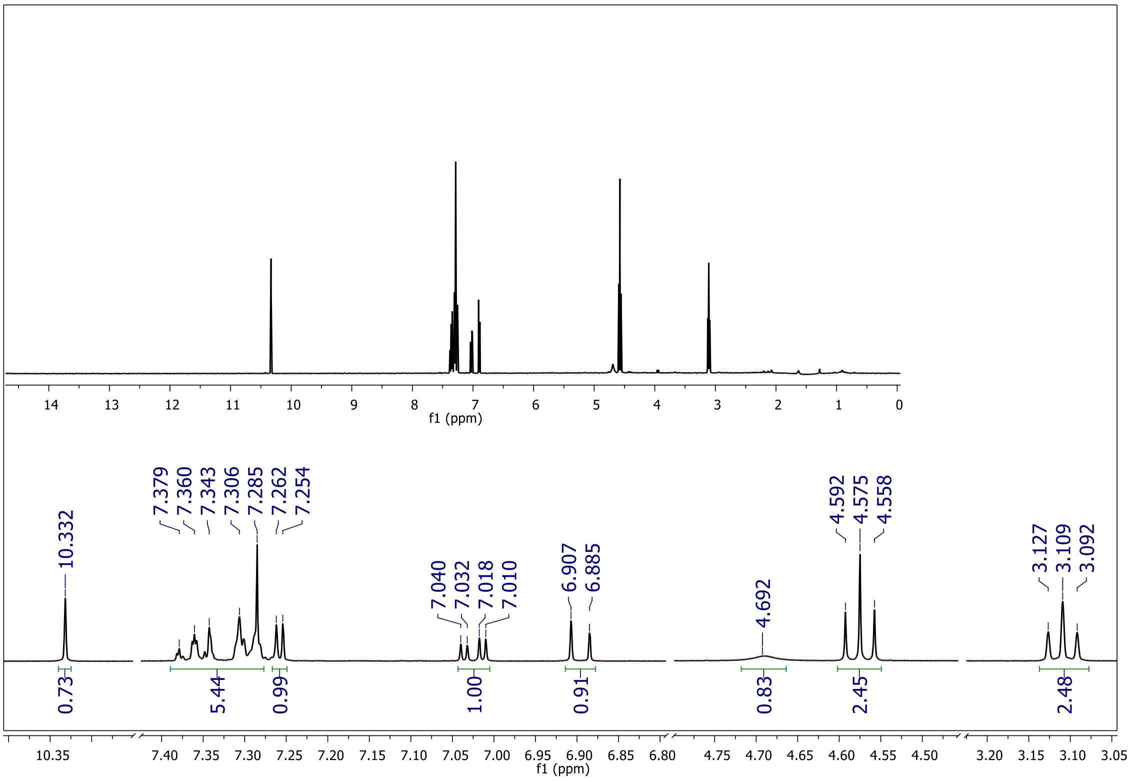

Figure S24. II) ^{13}C NMR spectrum of compound **24** (150 MHz – $CDCl_3$)

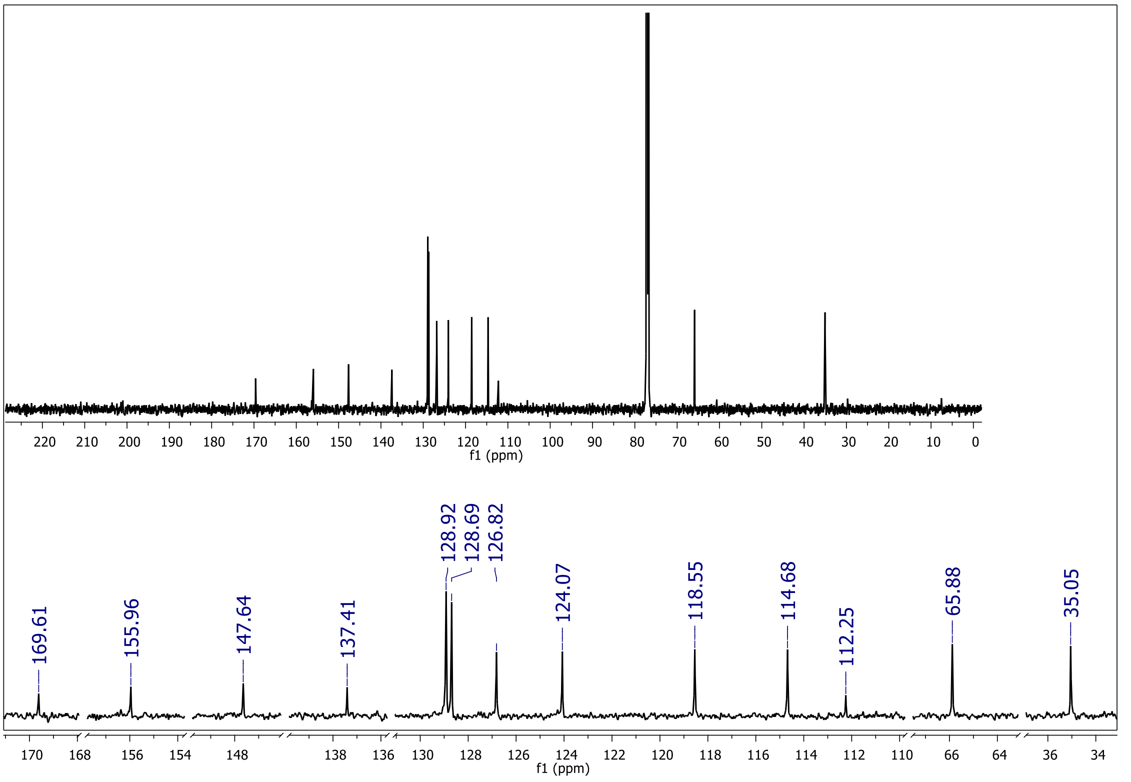

Figure S24. III) HPLC chromatogram of compound **24**

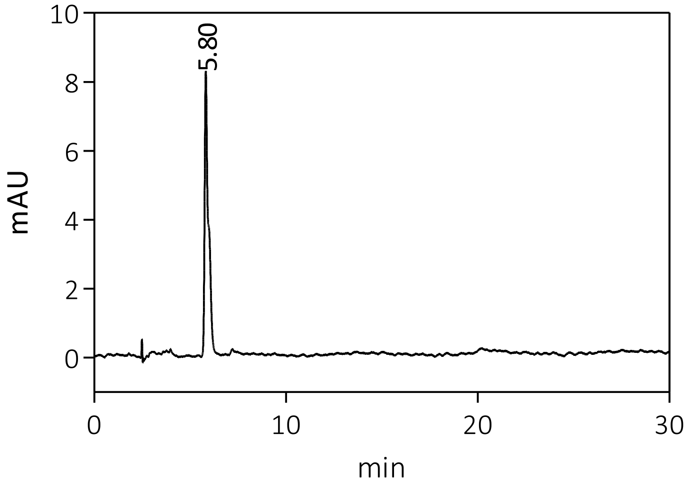

Figure S25. I) ^{1}H NMR spectrum of compound **25** (400 MHz – DMSO-d_6)

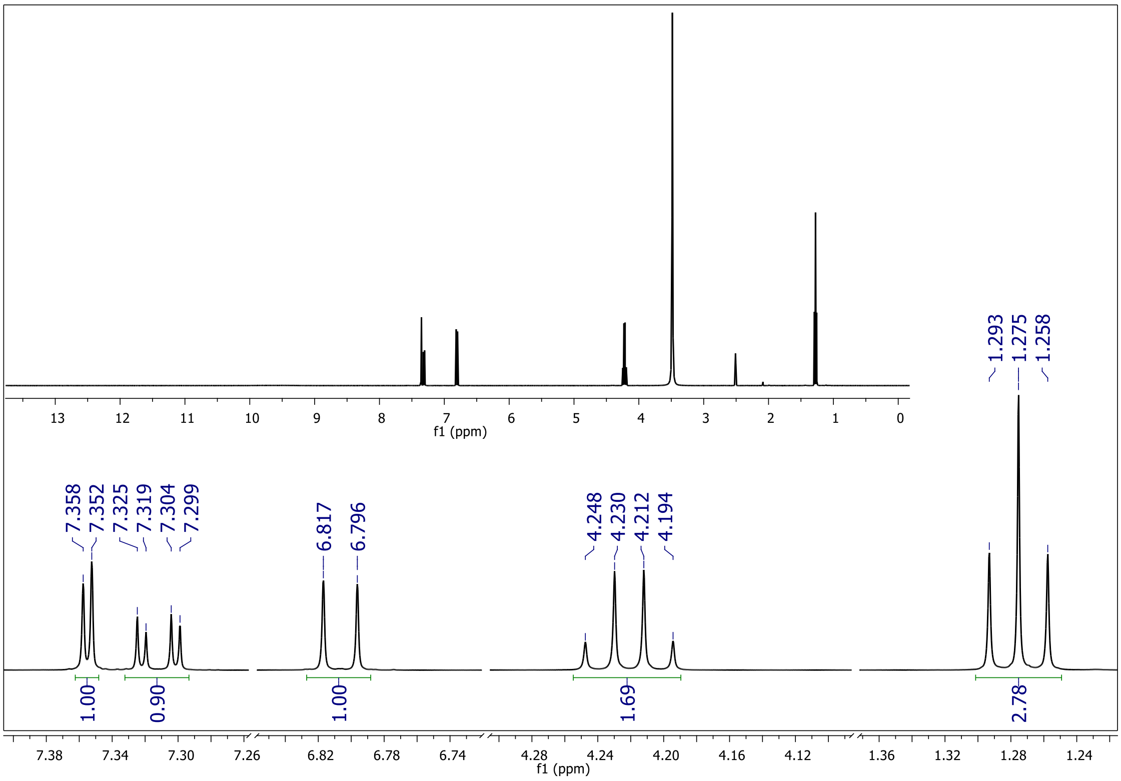

Figure S25. II) ^{13}C NMR spectrum of compound **25** (100 MHz – DMSO-d_6)

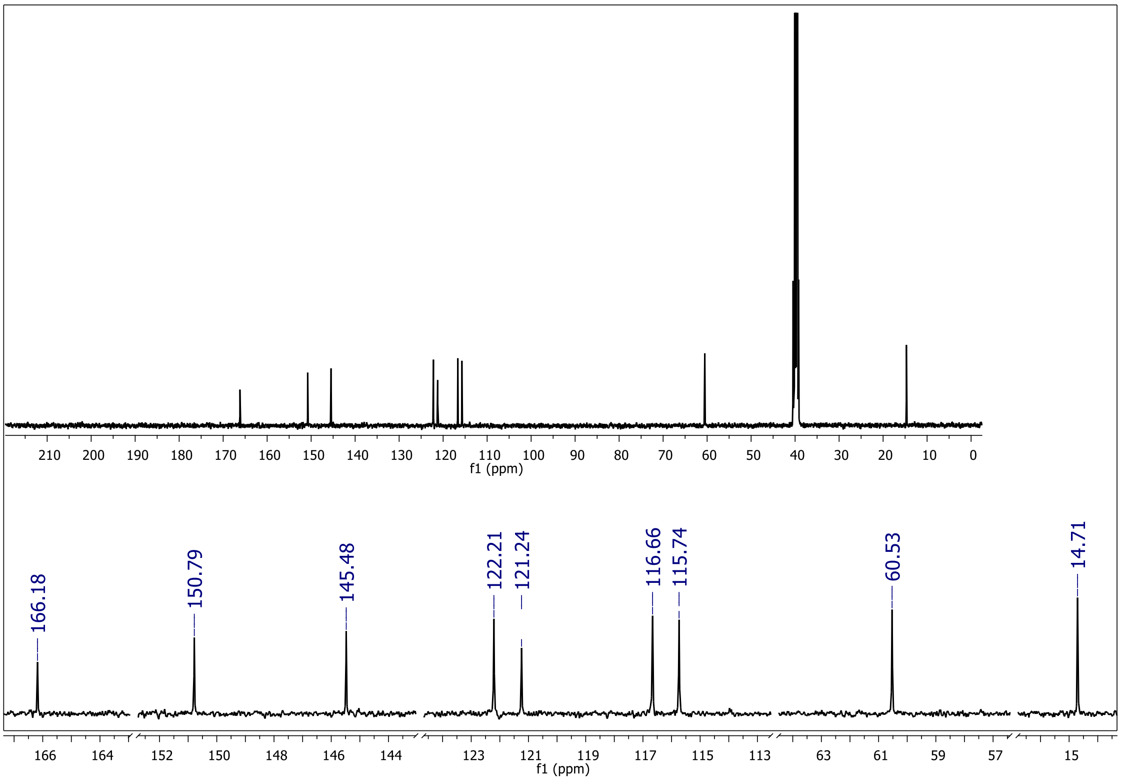

Figure S25. III) HPLC chromatogram of compound **25**

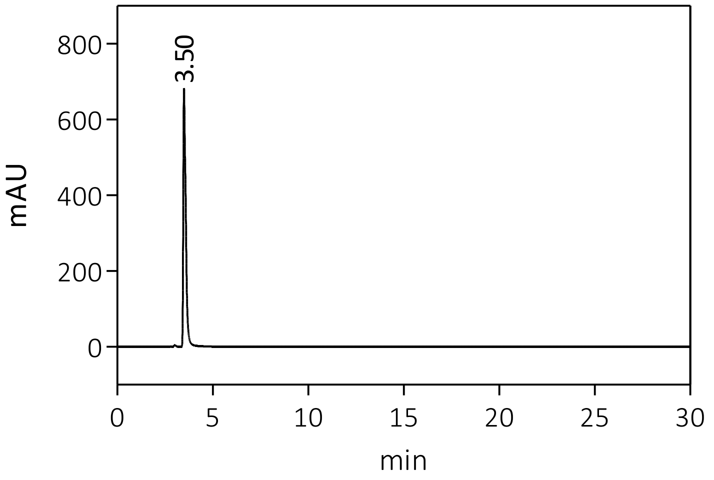

Figure S26. I) ^{1}H NMR spectrum of compound **26** (400 MHz – $CDCl_3$)

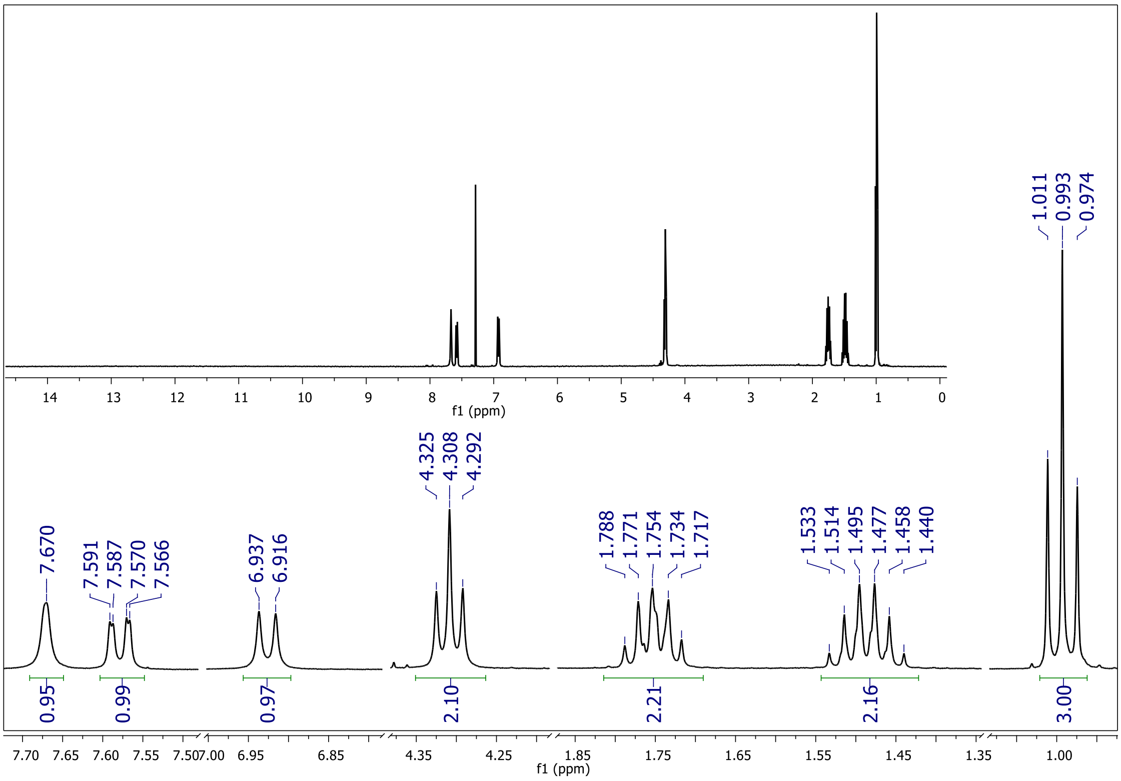

Figure S26. II) ^{13}C NMR spectrum of compound **26** (100 MHz – $CDCl_3$)

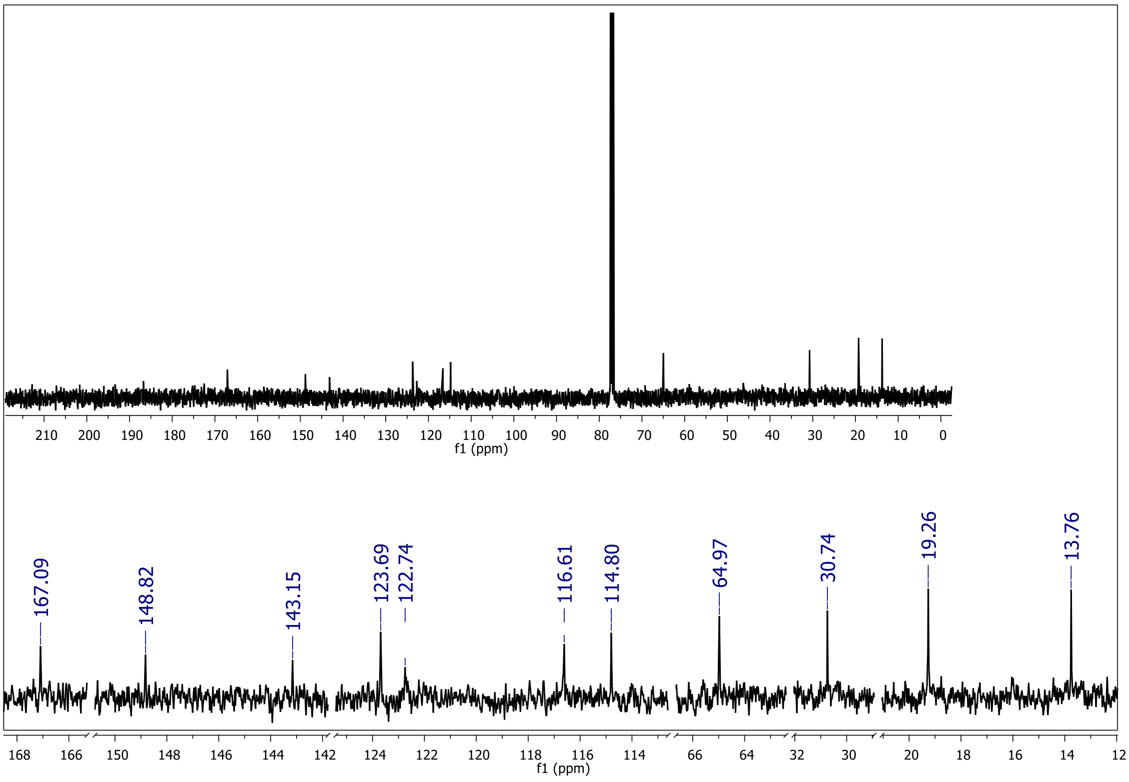

Figure S26. III) HPLC chromatogram of compound **26**

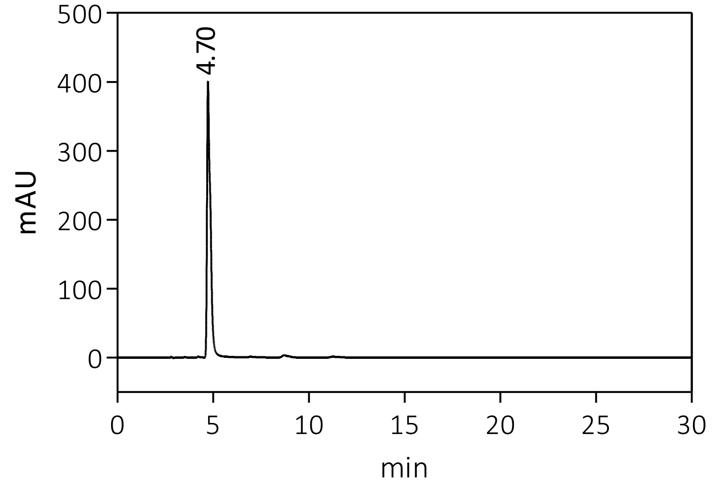

Figure S27. I) ^{1}H NMR spectrum of compound **27** (400 MHz – DMSO-d_6)

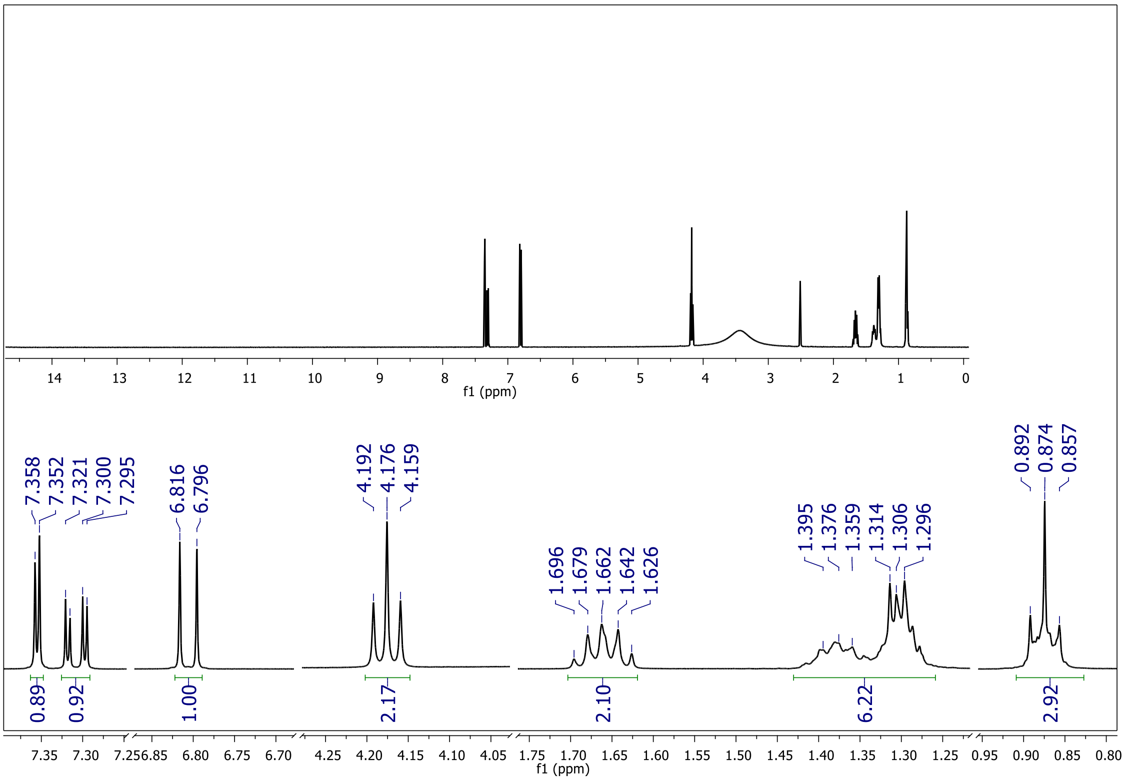

Figure S27. II) ^{13}C NMR spectrum of compound **27** (150 MHz – DMSO-d_6)

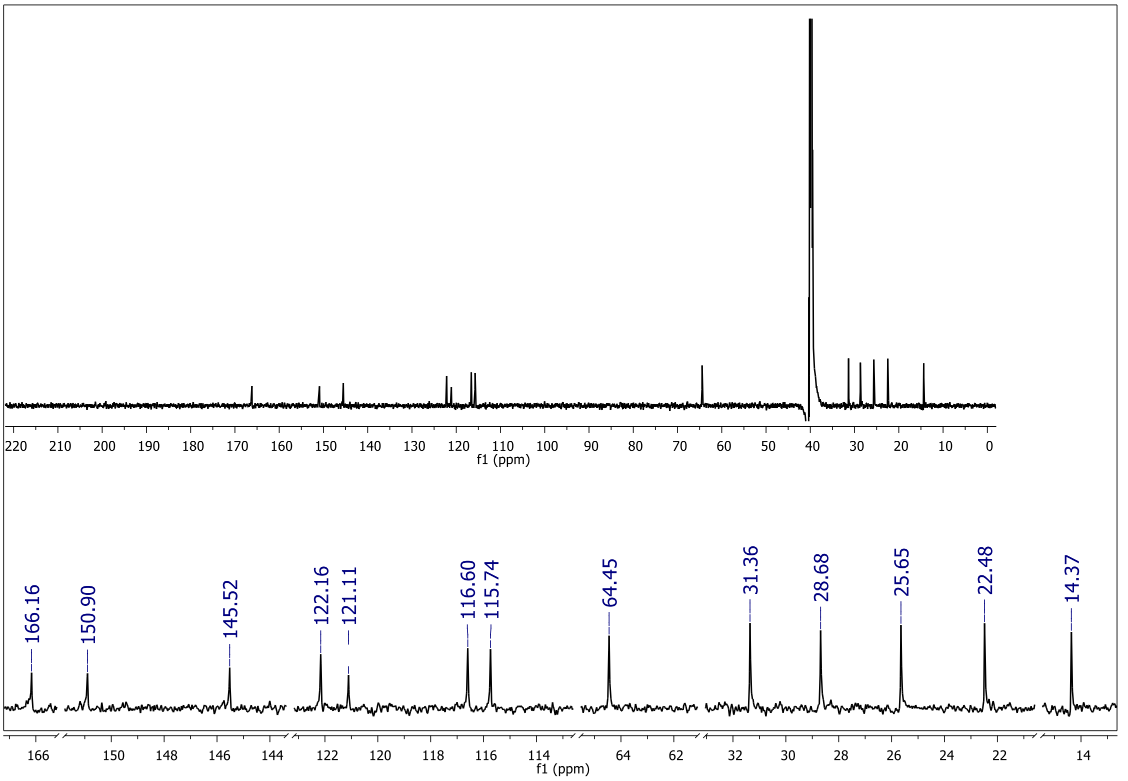

Figure S27. III) HPLC chromatogram of compound **27**

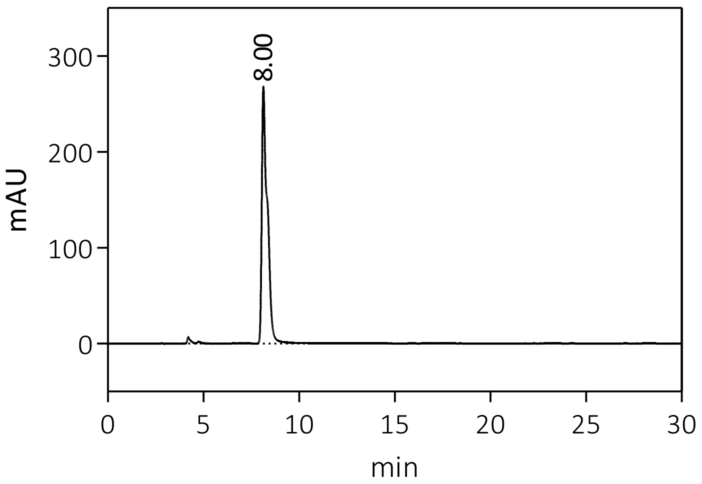

Figure S28. I) ^{1}H NMR spectrum of compound **28** (600 MHz – DMSO-d_6)

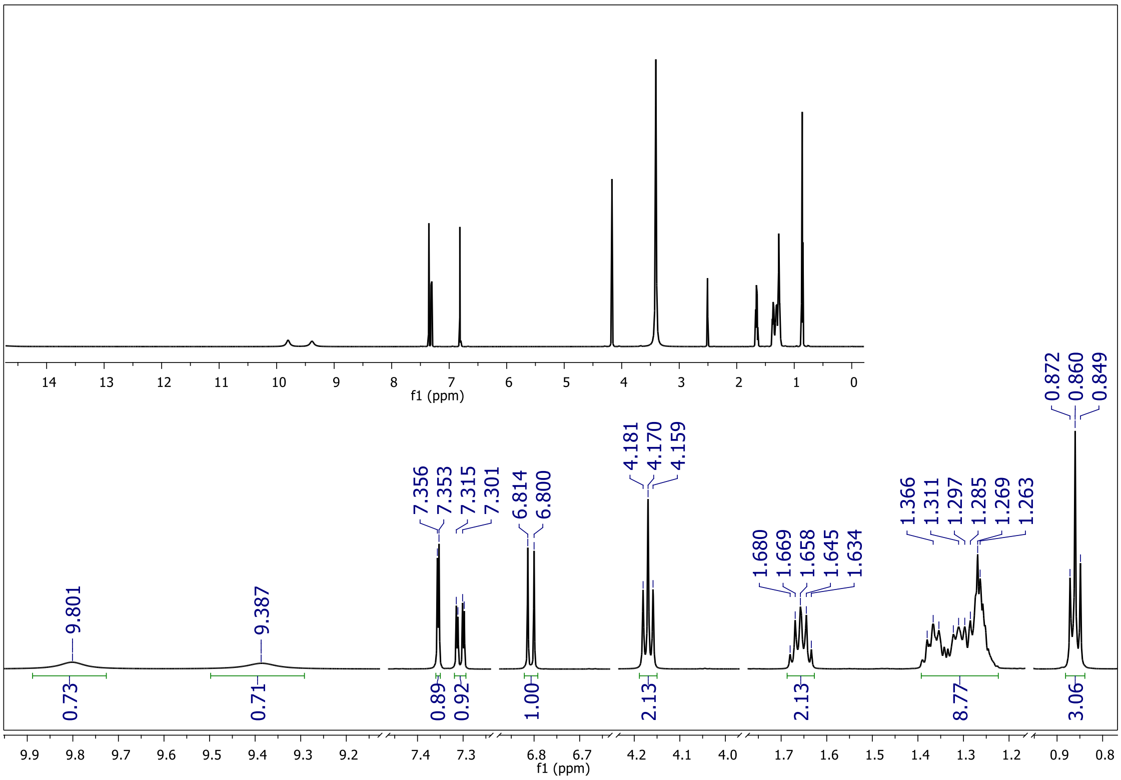

Figure S28. II) ^{13}C NMR spectrum of compound **28** (150 MHz – DMSO-d_6)

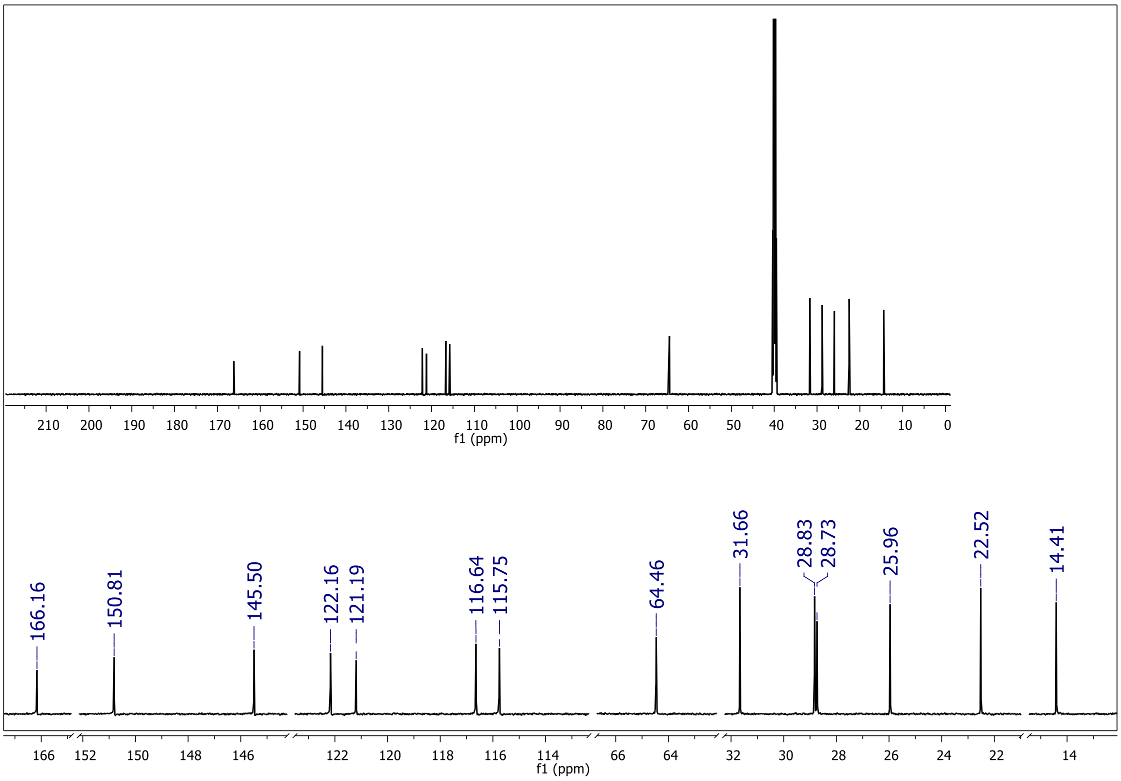

Figure S28. III) HPLC chromatogram of compound **28**

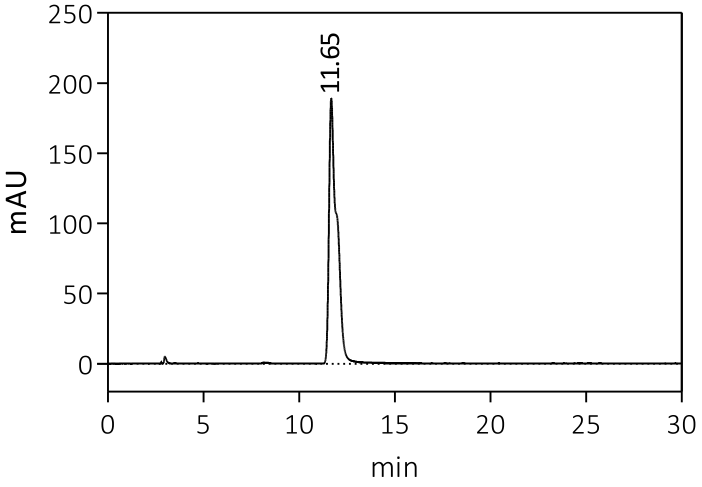

Figure S29. I) ^{1}H NMR spectrum of compound **29** (400 MHz – $CDCl_3$)

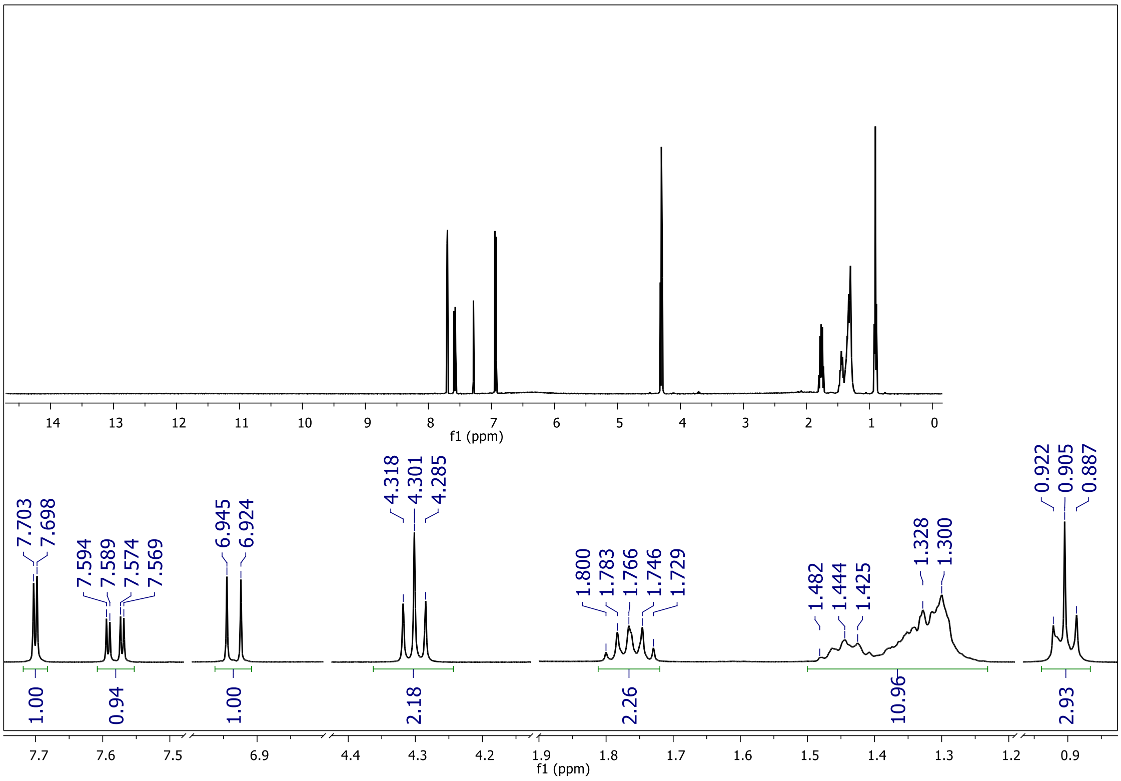

Figure S29. II) ^{13}C NMR spectrum of compound **29** (100 MHz – $CDCl_3$)

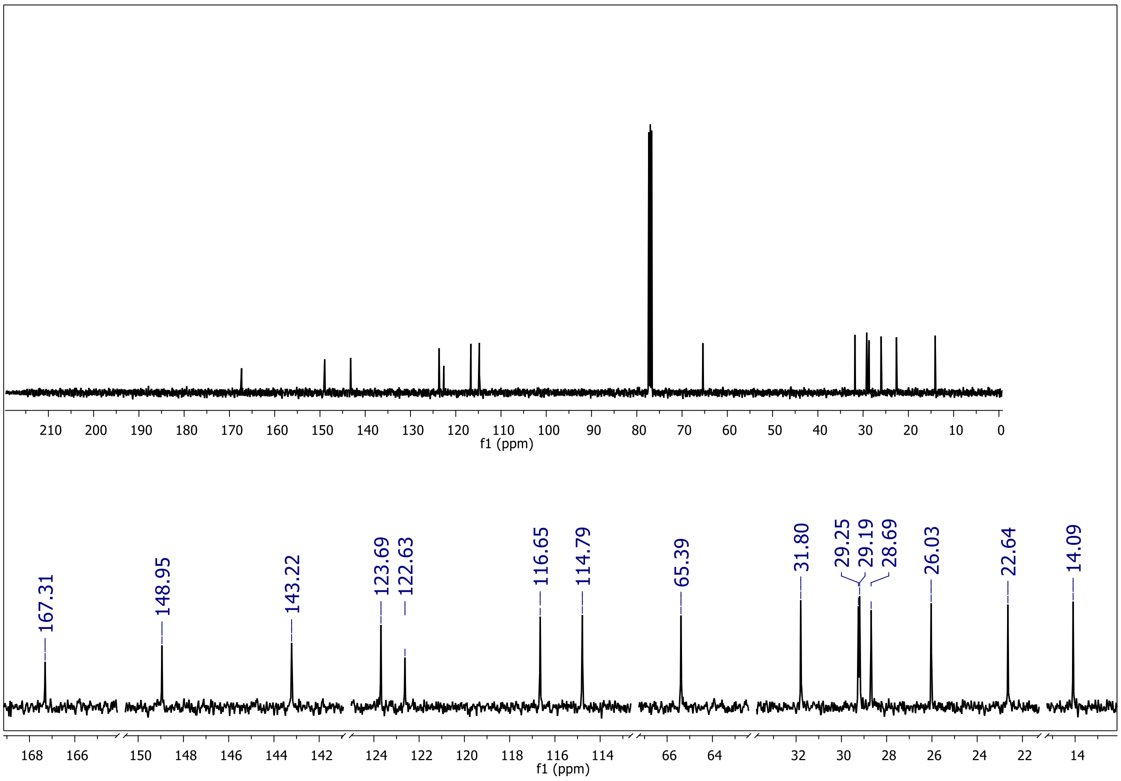

Figure S29. III) HPLC chromatogram of compound **29**

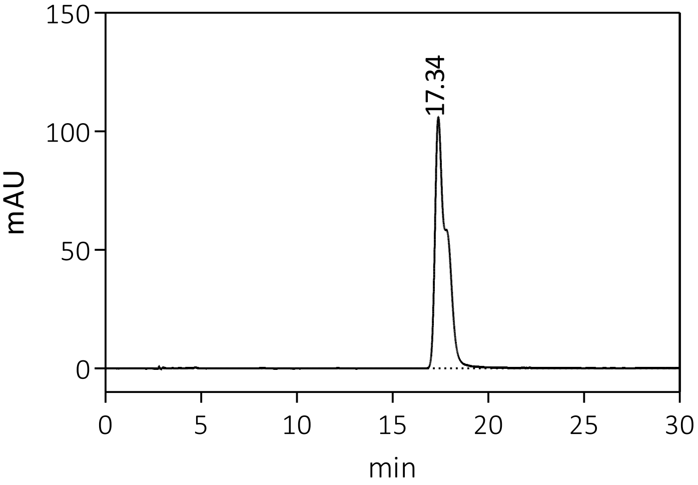

Figure S30. I) ^{1}H NMR spectrum of compound **30** (300 MHz – $CDCl_3$)

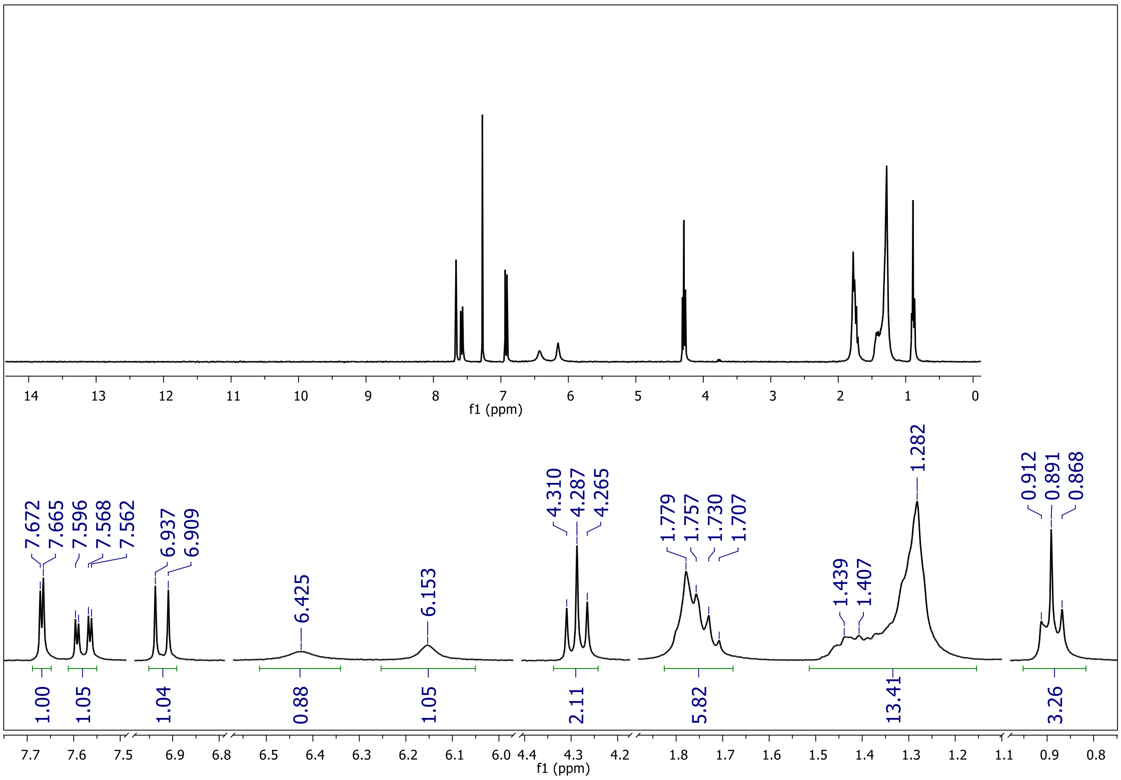

Figure S30. II) ^{13}C NMR spectrum of compound **30** (150 MHz – DMSO-d_6)

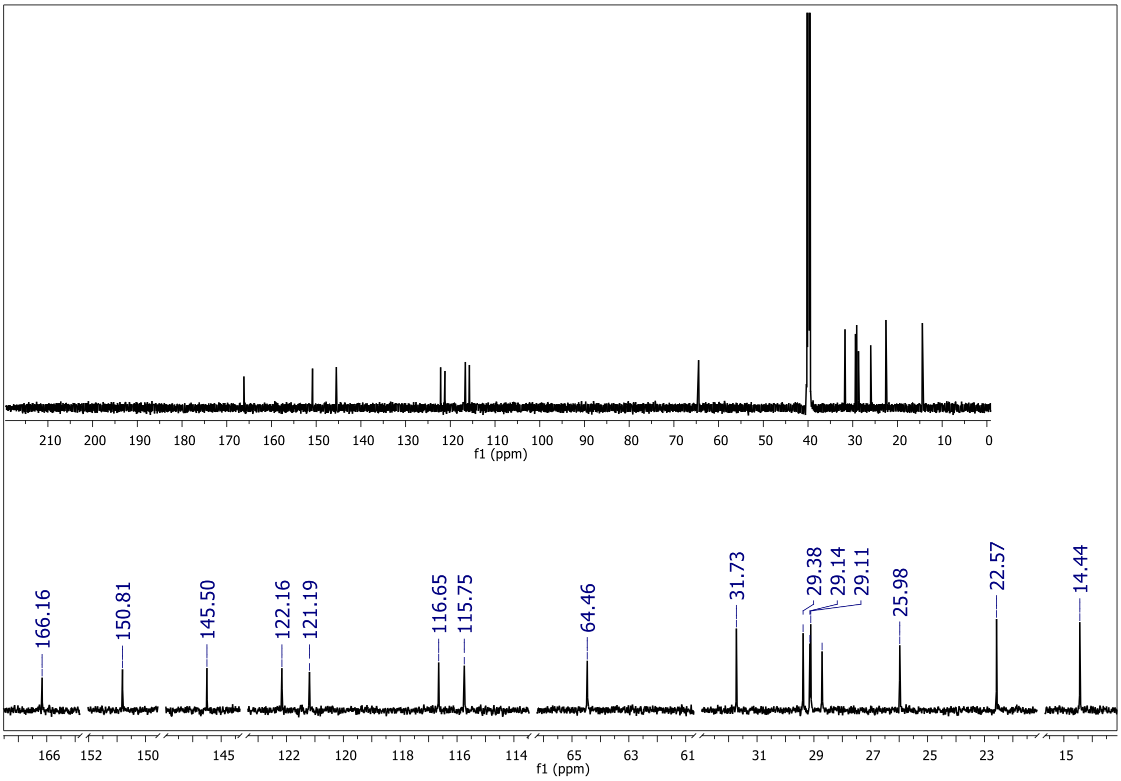

Figure S30. III) HPLC chromatogram of compound **30**

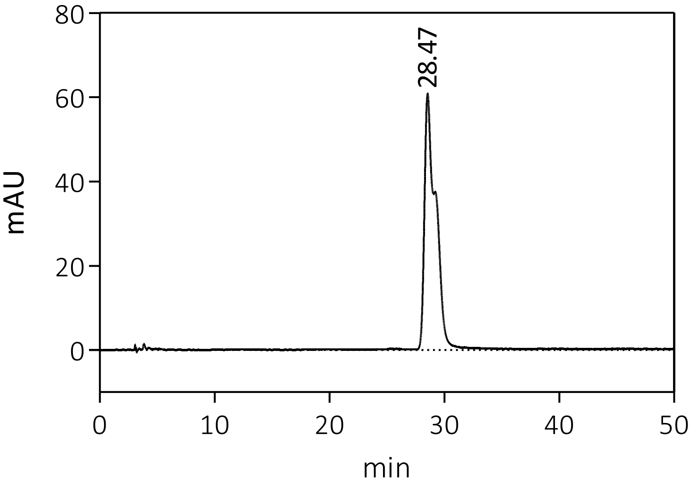

Figure S31. I) ^{1}H NMR spectrum of compound **31** (400 MHz – $CDCl_3$)

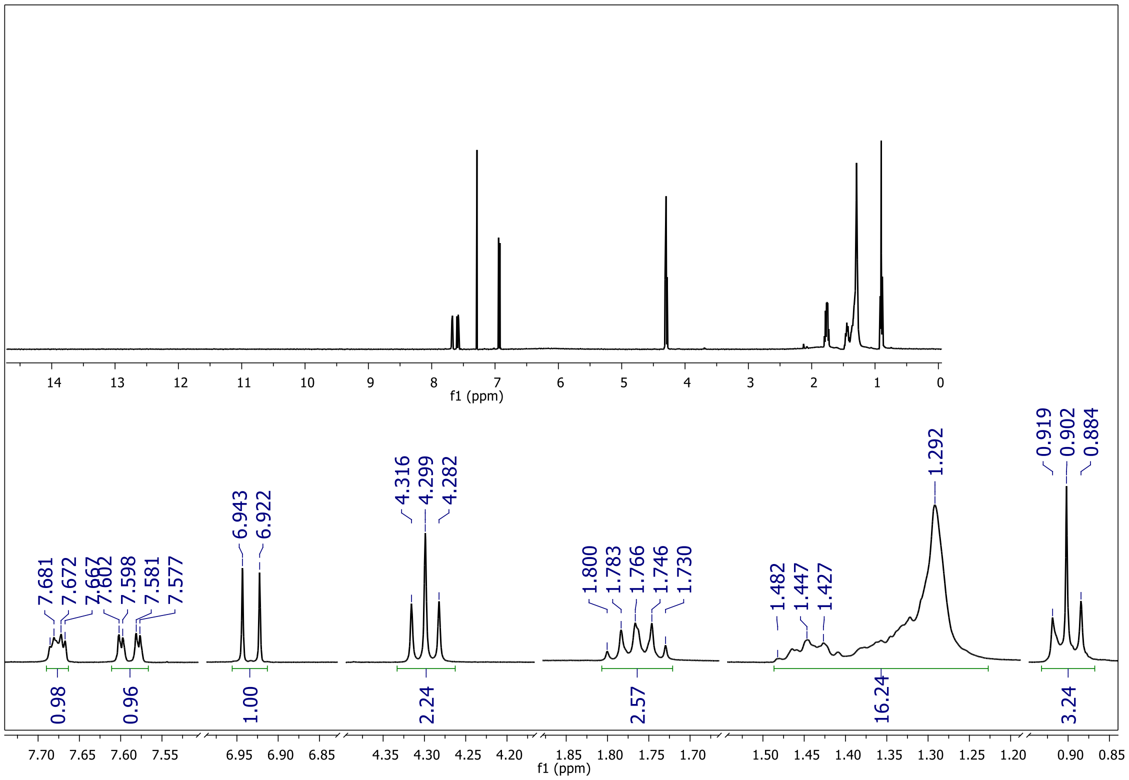

Figure S31. II) ^{13}C NMR spectrum of compound **31** (100 MHz – $CDCl_3$)

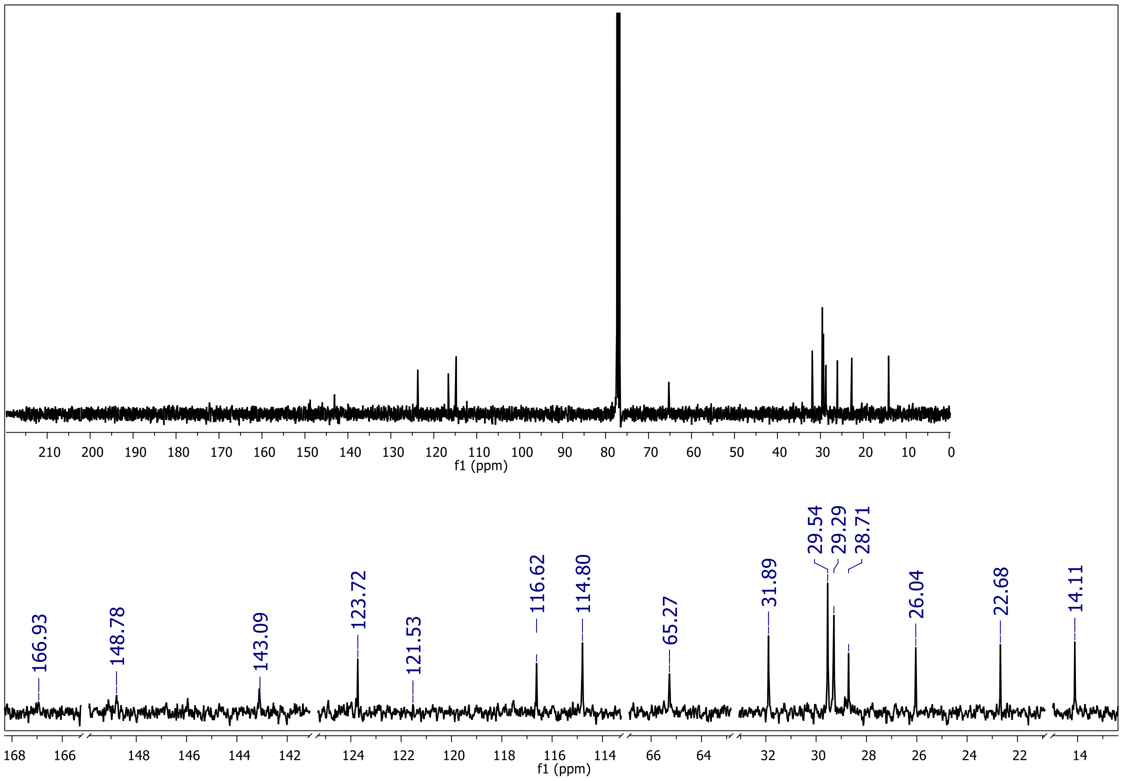

Figure S31. III) HPLC chromatogram of compound **31**

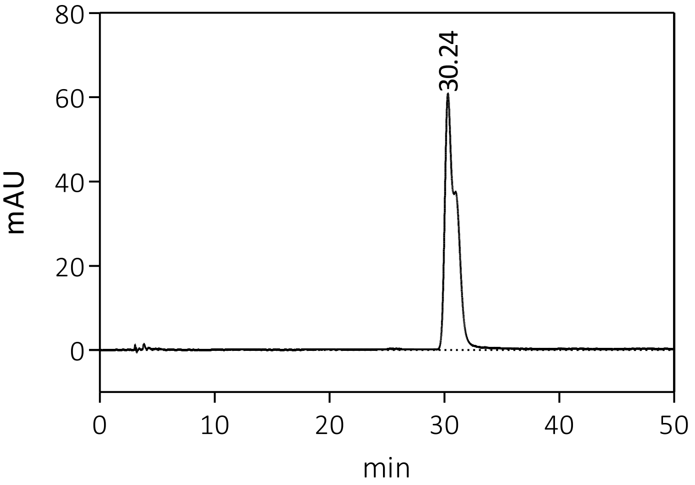

Figure S32. I) ^{1}H NMR spectrum of compound **32** (300 MHz – $CDCl_3$)

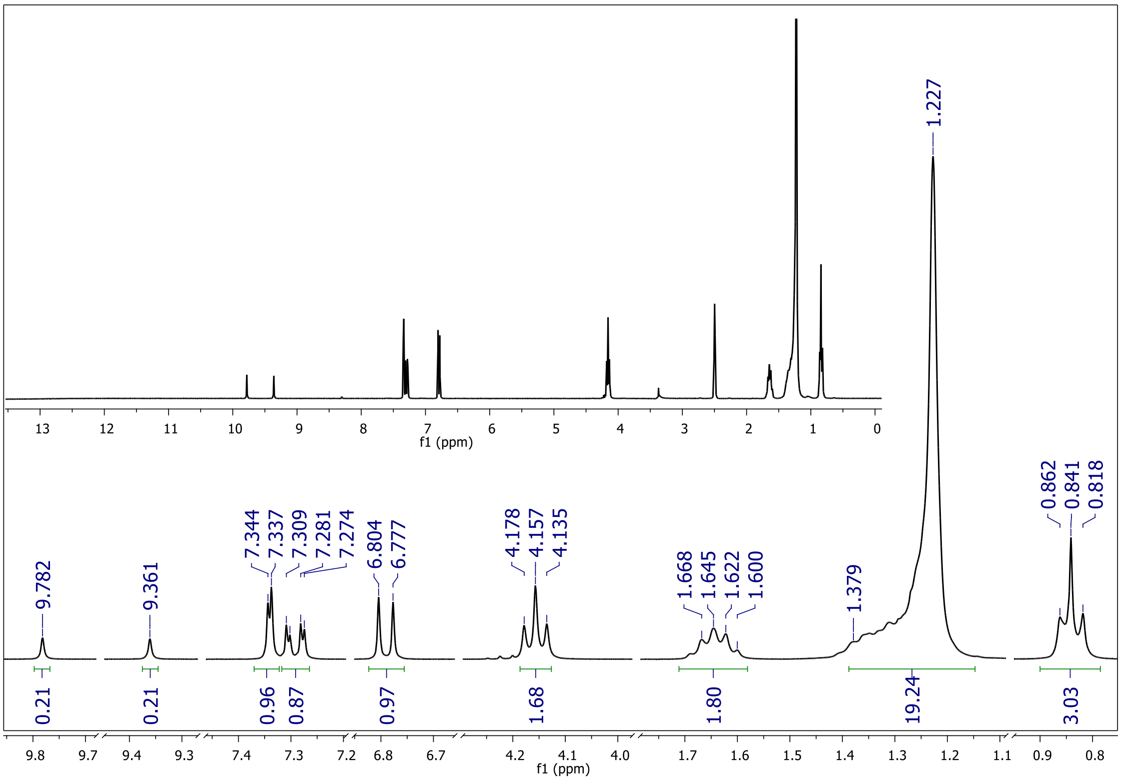

Figure S32. II) ^{13}C NMR spectrum of compound **32** (100 MHz – $CDCl_3$)

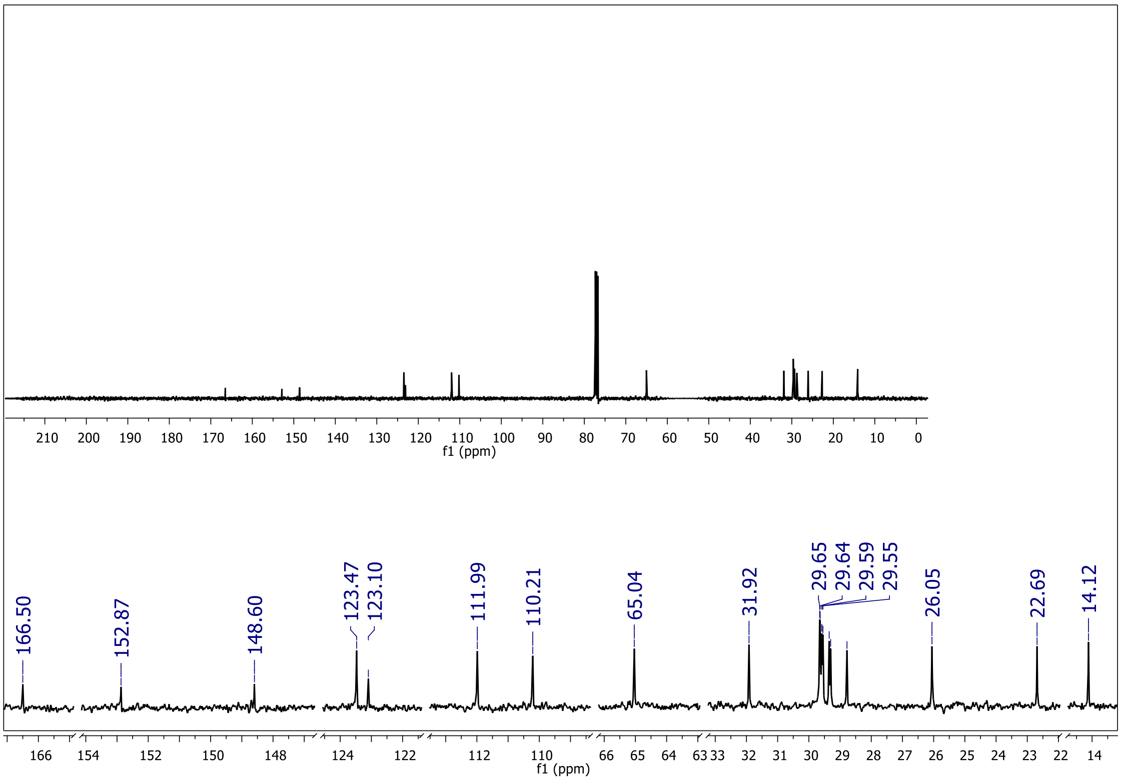

Figure S32. III) HPLC chromatogram of compound **32**

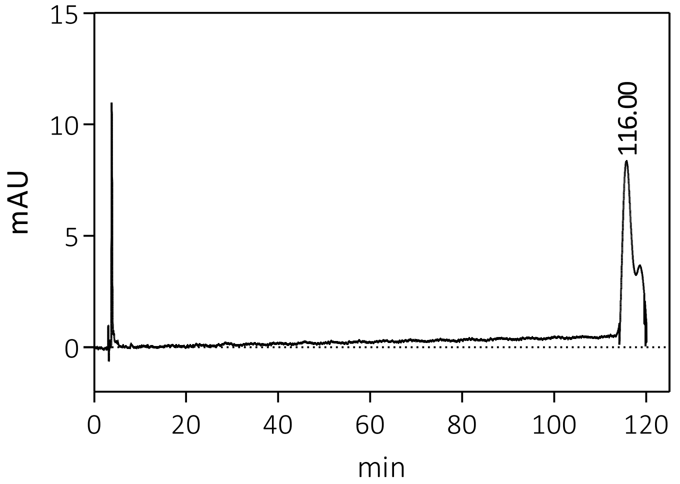

Figure S33. I) ^{1}H NMR spectrum of compound **33** (300 MHz – DMSO-d_6)

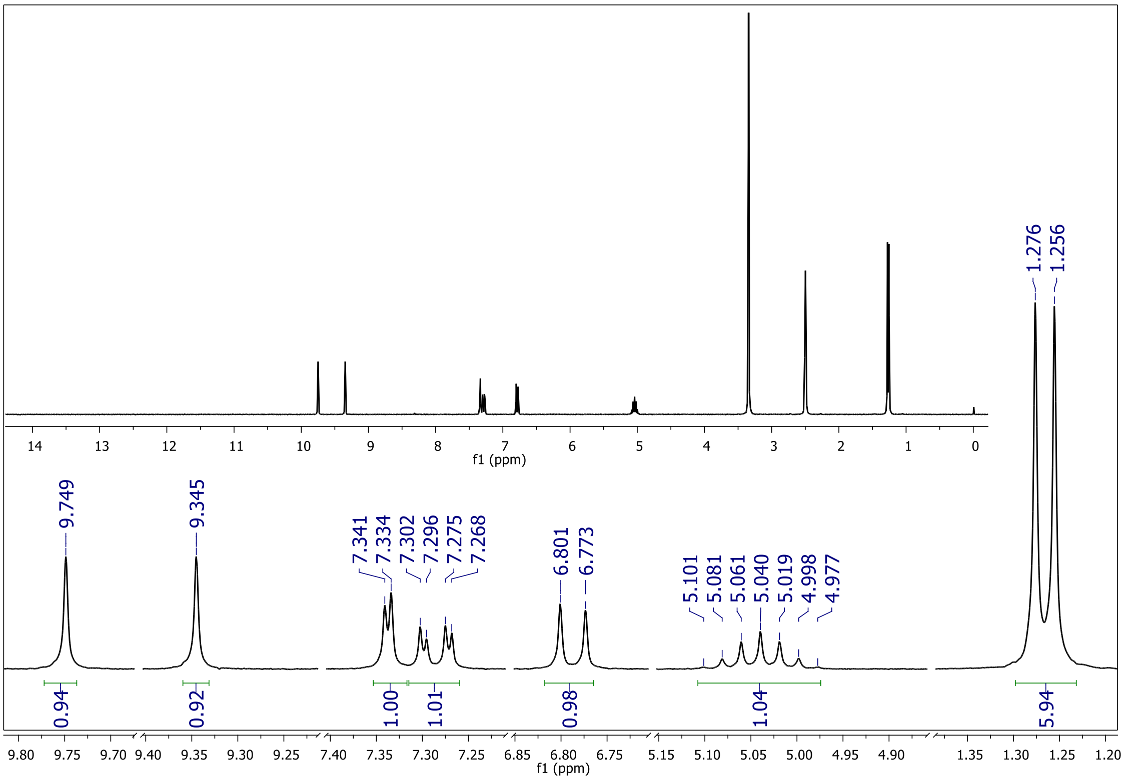

Figure S33. II) ^{13}C NMR spectrum of compound **33** (150 MHz – DMSO-d_6)

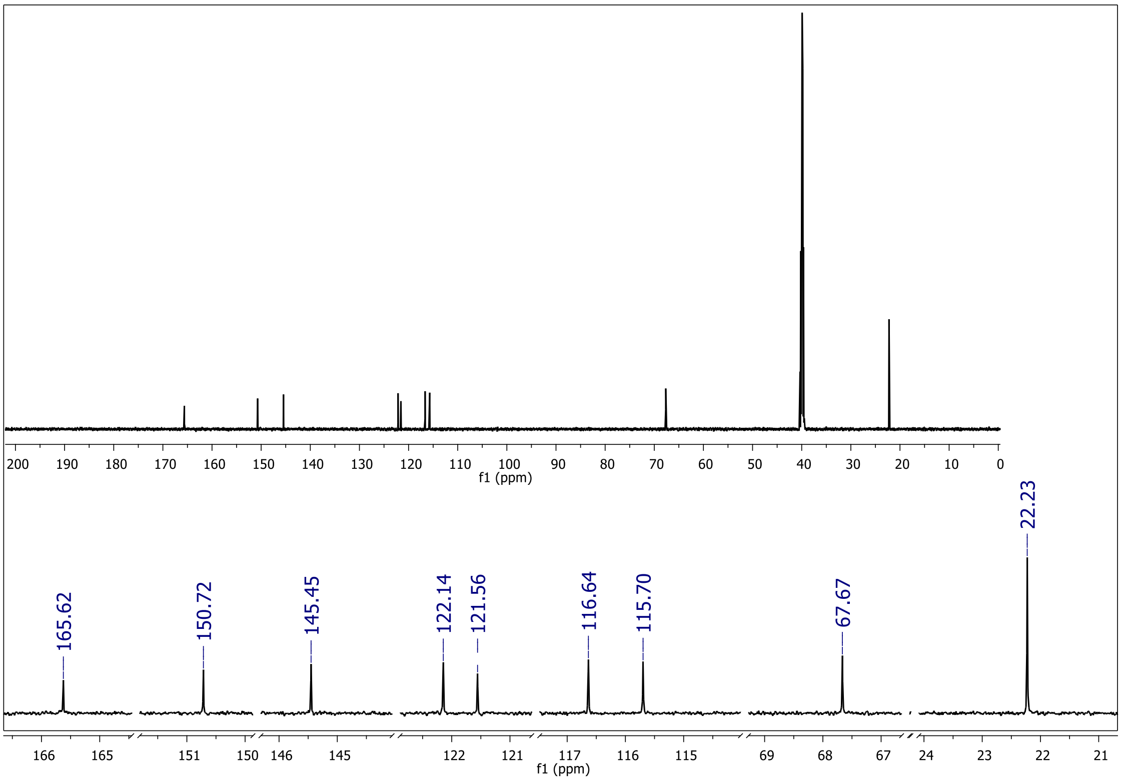

Figure S33. III) HPLC chromatogram of compound **33**

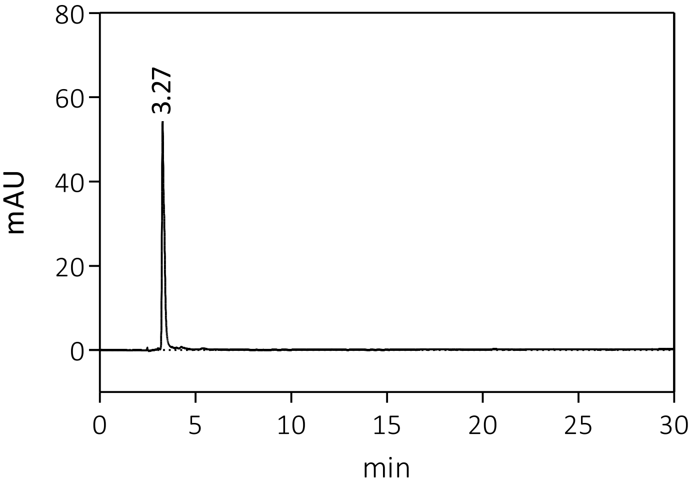

Figure S34. I) ^{1}H NMR spectrum of compound **34** (300 MHz – DMSO-d_6)

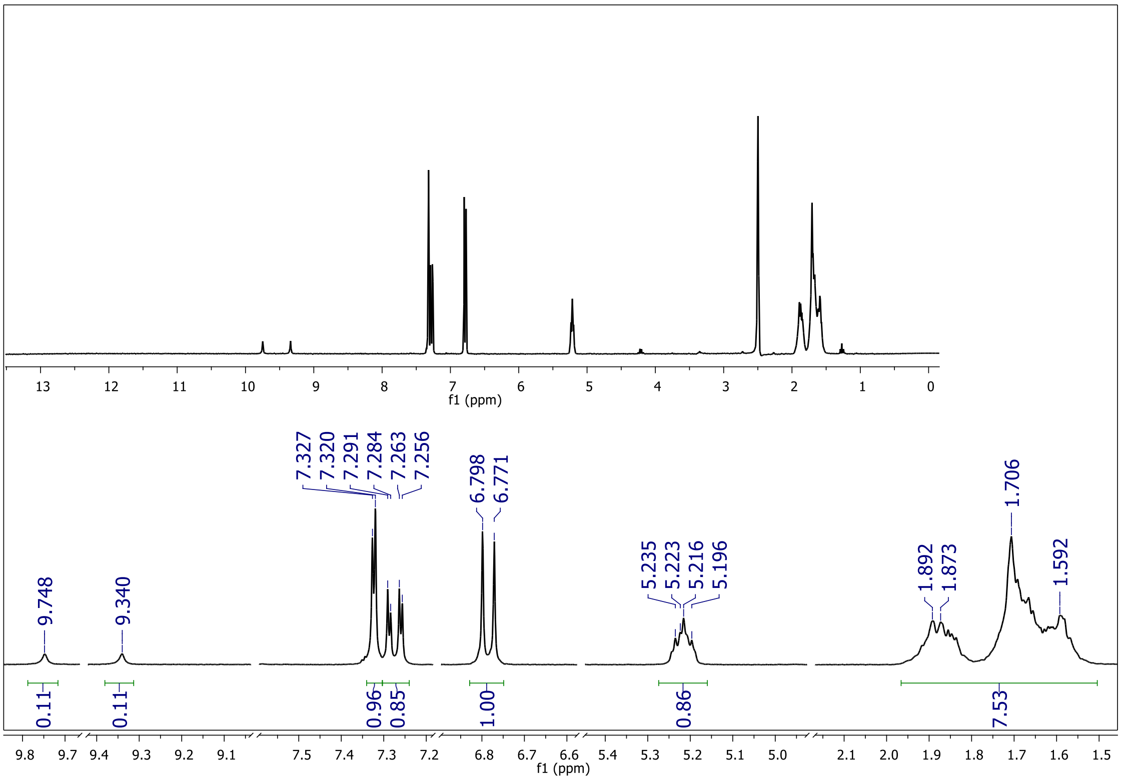

Figure S34. II) ^{13}C NMR spectrum of compound **34** (150 MHz – DMSO-d_6)

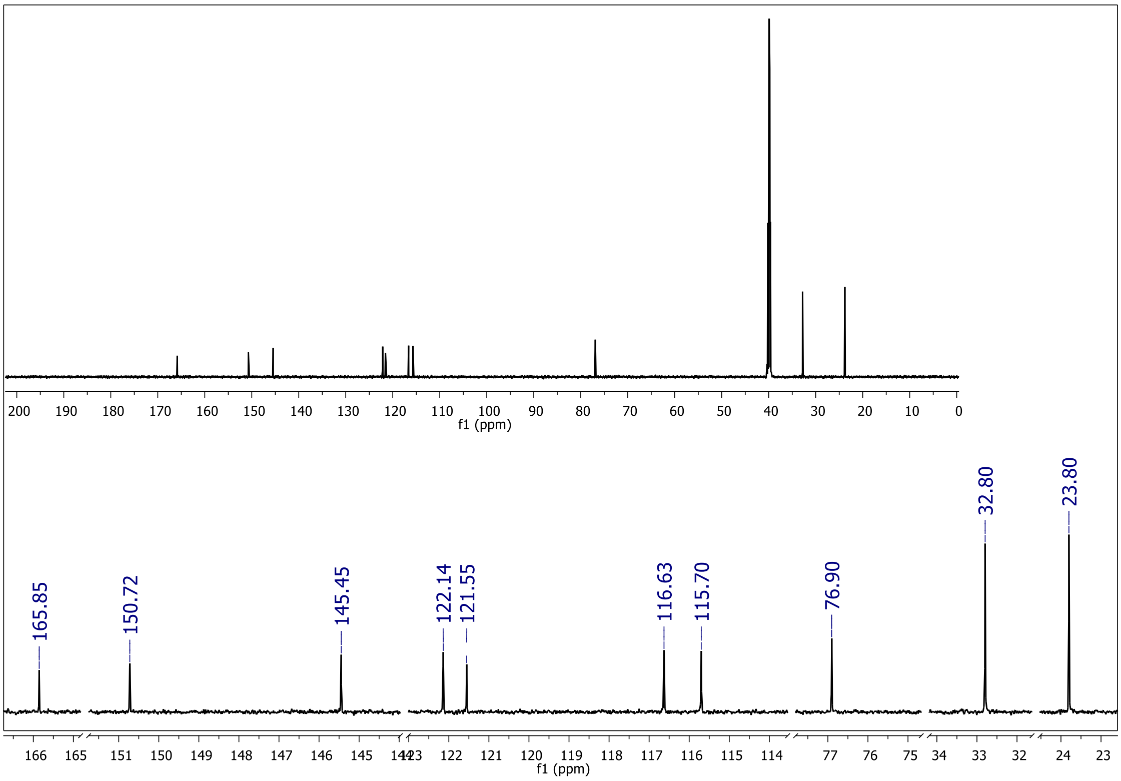

Figure S34. III) HPLC chromatogram of compound **34**

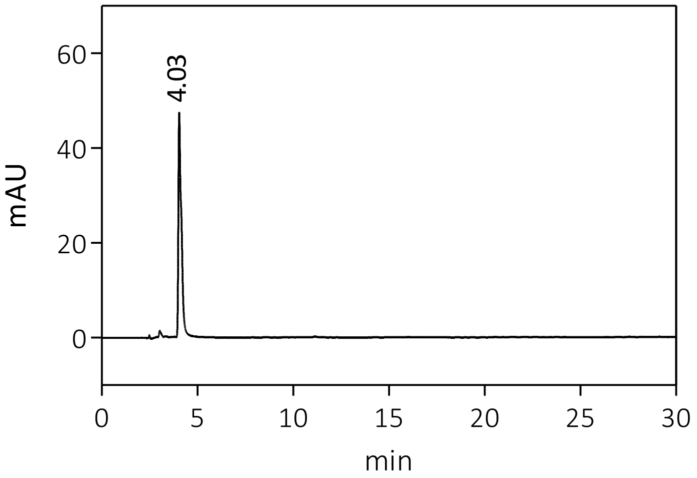

Figure S35. I) ^{1}H NMR spectrum of compound **35** (600 MHz – DMSO-d_6)

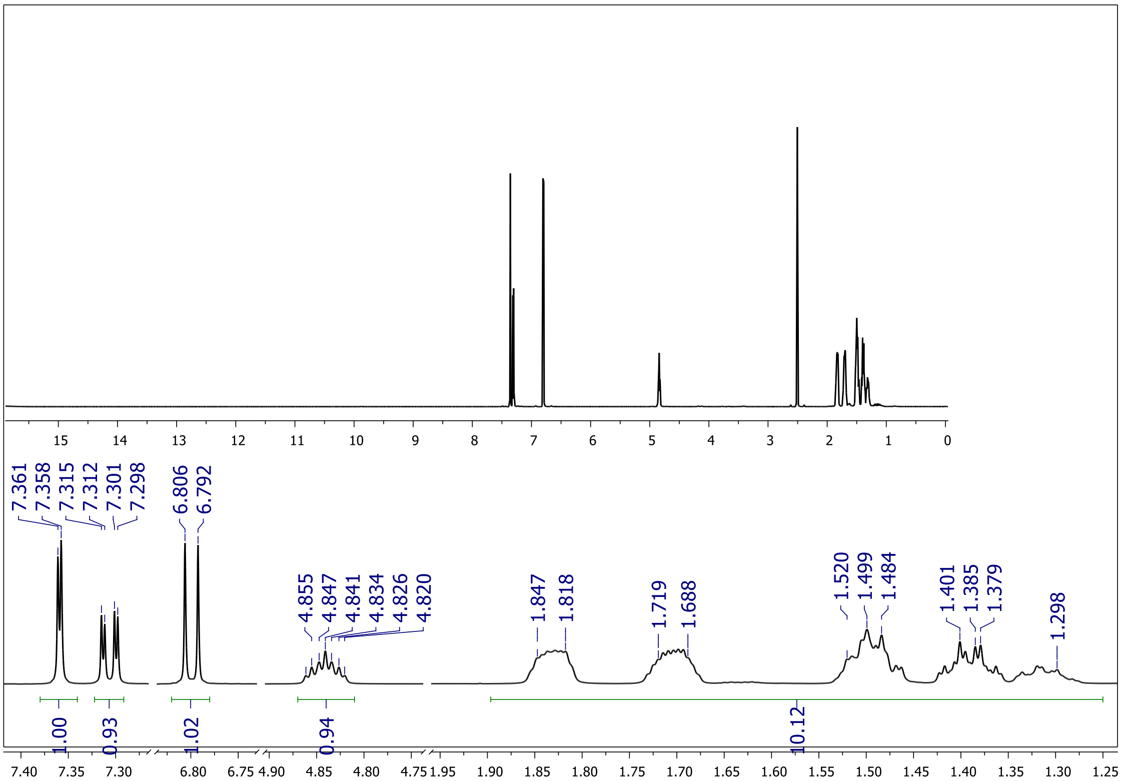

Figure S35. II) ^{13}C NMR spectrum of compound **35** (150 MHz – DMSO-d_6)

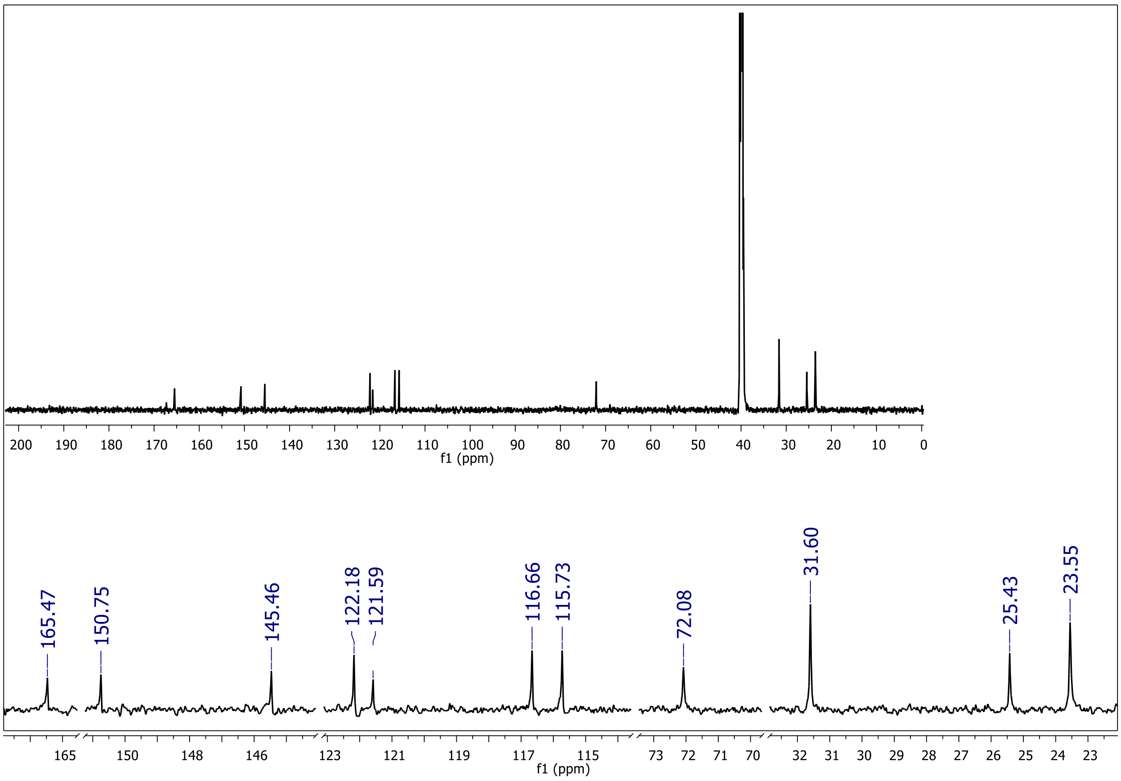

Figure S35. III) HPLC chromatogram of compound **35**

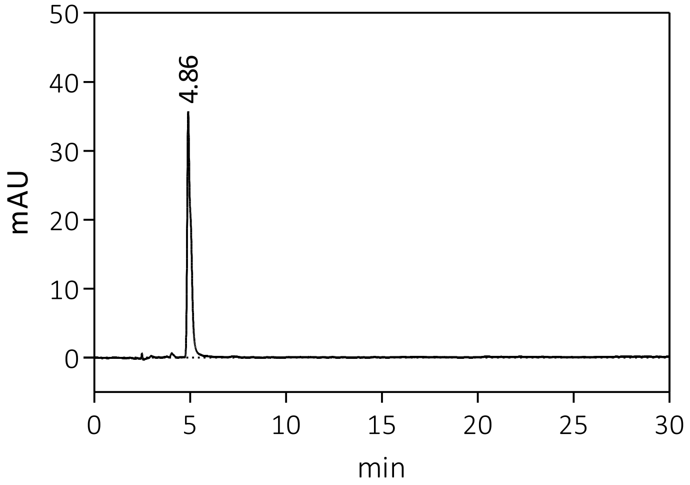

Figure S36. I) ^{1}H NMR spectrum of compound **36** (300 MHz – DMSO-d_6)

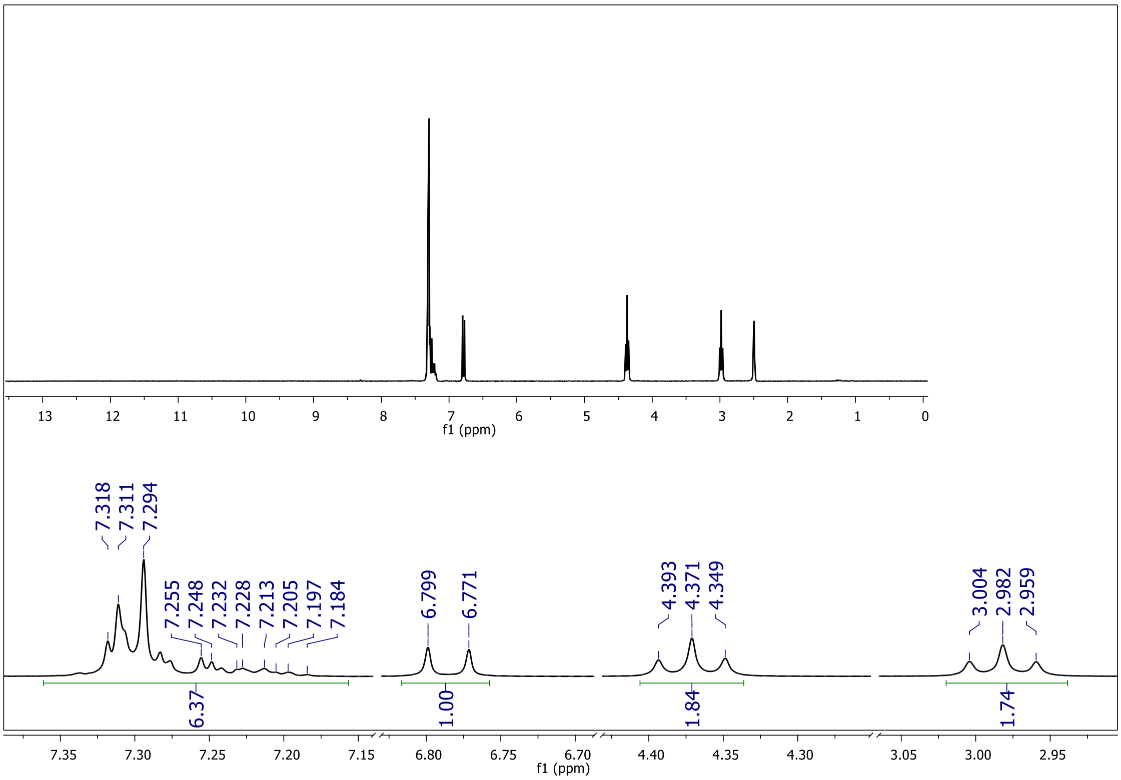

Figure S36. II) ^{13}C NMR spectrum of compound **36** (150 MHz – DMSO-d_6)

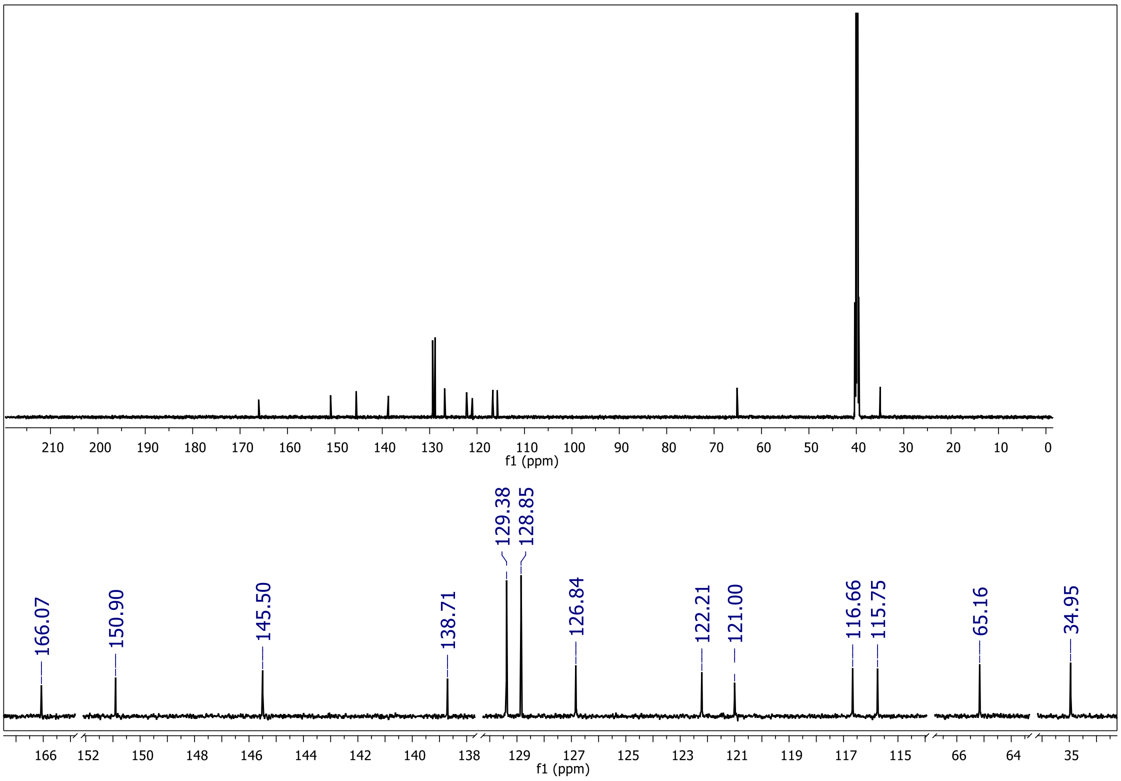

Figure S36. III) HPLC chromatogram of compound **36**

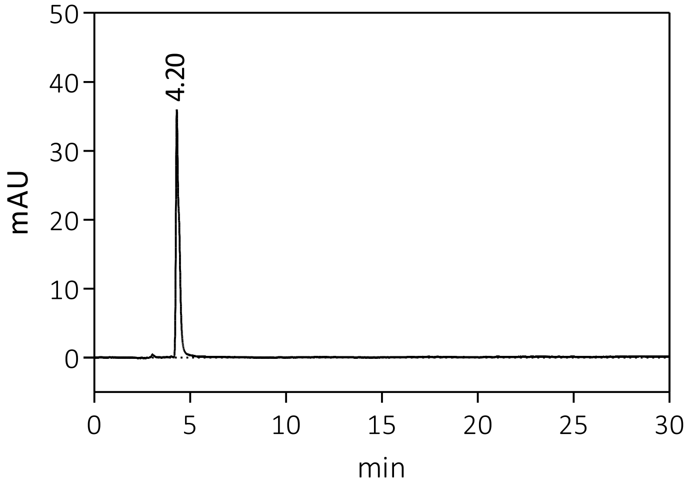

Supplement: Supplementary file 1 [file ijms-19-03050-s001.pdf]
